# Enantiomers Self-Sort into Separate Counter-Twisted Ribbons of the *Fddd* Liquid Crystal—Antiferrochirality and Parachirality

**DOI:** 10.1021/jacs.3c06164

**Published:** 2023-07-31

**Authors:** Yan Wang, Ya-Xin Li, Liliana Cseh, Yong-Xuan Chen, Shu-Gui Yang, Xiangbing Zeng, Feng Liu, Wenbing Hu, Goran Ungar

**Affiliations:** † Shaanxi International Research Centre for Soft Matter, State Key Laboratory for Mechanical Behaviour of Materials, Xi’an Jiaotong University, Xi’an 710049, China; ‡ School of Chemistry and Chemical Engineering, Henan University of Technology, Zhengzhou 450001, China; § Romanian Academy, Coriolan Dragulescu Institute of Chemistry, Timisoara 300223, Romania; ∥ State Key Laboratory of Coordinate Chemistry, School of Chemistry and Chemical Engineering, Nanjing University, Nanjing 210093, China; ⊥ Department of Materials Science and Engineering, University of Sheffield, Sheffield S1 3JD, U.K.

## Abstract

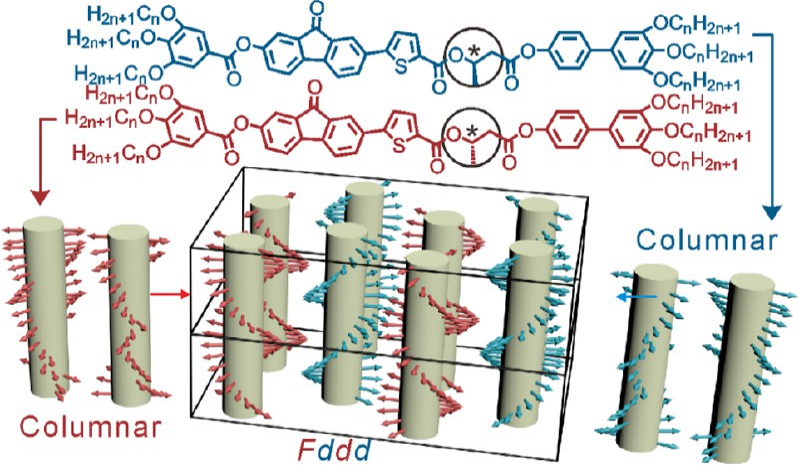

The recently discovered orthorhombic liquid crystal (LC) phase of symmetry *Fddd* is proving to be widespread. In this work, a chiral hydroxybutyrate linkage is inserted into the molecular core of hexacatenar rodlike compounds, containing a thienylfluorenone fluorophore. In addition to more usual tools, the methods used include grazing-incidence X-ray scattering, modulated differential scanning calorimetry (DSC), flash DSC with rates up to 6000 K/s, and chiro-optical spectroscopies using Mueller matrix method, plus conformational mapping. Although pure *R* and *S* enantiomers form only a strongly chiral hexagonal columnar LC phase (Col_h_*), the racemic mixture forms a highly ordered *Fddd* phase with 4 right- and 4 left-handed twisted ribbon-like columns traversing its large unit cell. In that structure, the two enantiomers locally deracemize and self-sort into the columns of their preferred chirality. The twisted ribbons in *Fddd*, with a 7.54 nm pitch, consist of stacked rafts, each containing ∼2 side-by-side molecules, the successive rafts rotated by 17°. In contrast, an analogous achiral compound forms only the columnar phase. The multiple methods used gave a comprehensive picture and helped in-depth understanding not only of the *Fddd* phase but also of the “parachiral” Col_h_* in pure enantiomers with irregular helicity, whose chirality is compared to the magnetization of a paramagnet in a field. Unusual short-range ordering effects are also described. An explanation of these phenomena is proposed based on conformational analysis. Surprisingly, the isotropic–columnar transition is extremely fast, completing within ∼20 ms. A clear effect of phase on UV–vis absorption and emission is observed.

## 1 Introduction

**Figure 1:**
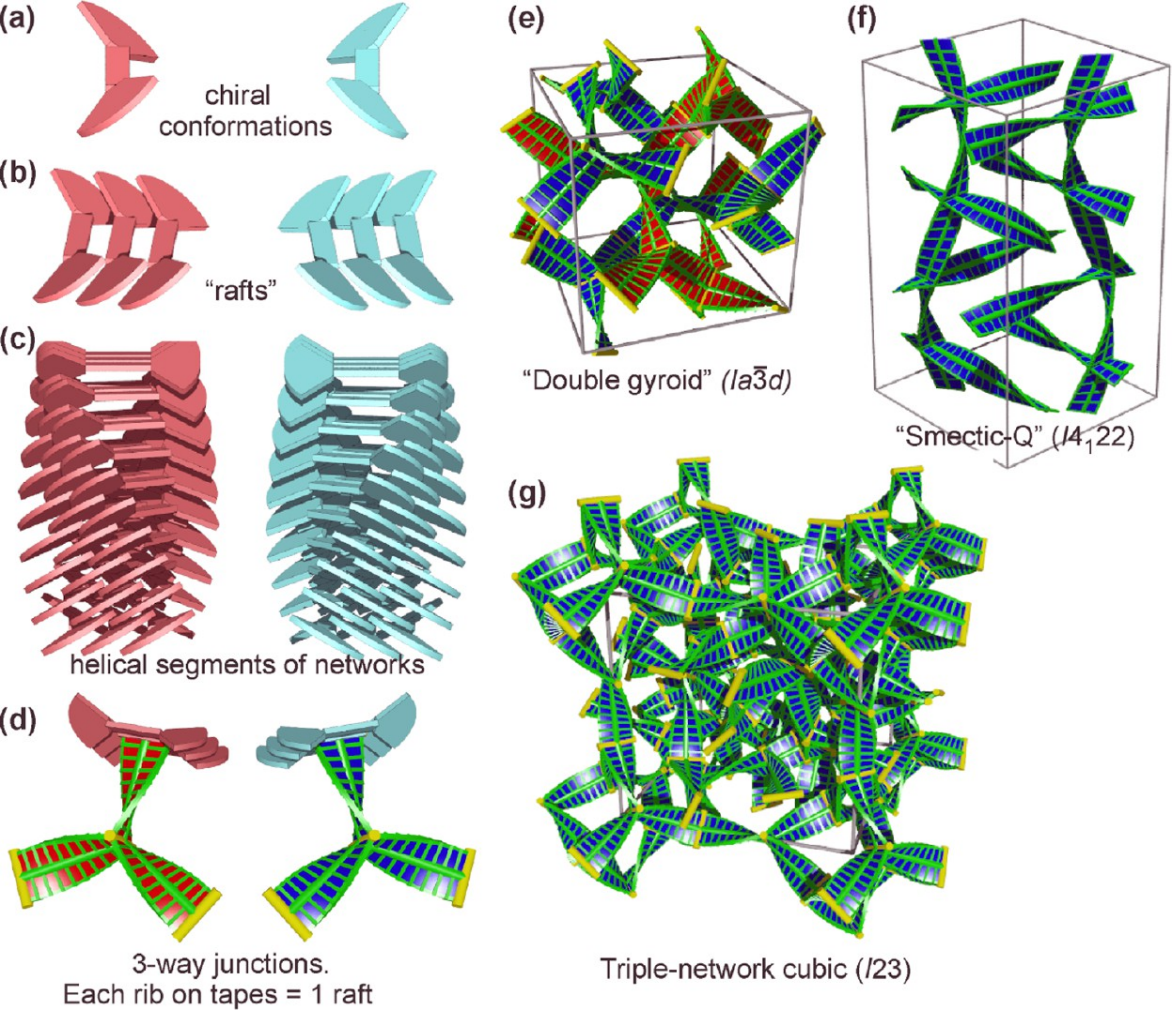
Schematic models of the self-assembly of achiral polycatenar molecules into 3D-ordered thermotropic bicontinuous phases. (a) Two molecules in antichiral conformations. Middle rod = rigid, usually aromatic, core; fans = multiple (2 or 3) flexible end chains attached, e.g., to a gallate ring. (b) “Rafts” of typically 2–4 molecules that stack into helical columnar segments (c). (d) Junctions connecting 3 helical segments that are represented as twisted ribbons.^18^ One raft is shown for illustration. (e–g) Ribbon models of the three bicontinuous phases in thermotropic LCs: (e) “Double-gyroid” cubic (achiral),^14^ (f) double-network tetragonal “Smectic-Q” (chiral),^16^ and (g) triple-network cubic (chiral).^18^ Space group symbols are indicated in parentheses.

Rodlike molecules, typically aromatic, with a flexible chain attached to one or both ends form liquid crystals (LC), displaying well-studied nematic and smectic mesophases. When two or more chains are attached at one or both ends (schematic Figure 1a), these so-called polycatenar compounds often exhibit either a two-dimensional (2D)-ordered columnar phase or a three-dimensional (3D)-ordered phase (straight core^1−7^ or bent core^8−13^). In polycatenar compounds, there are three confirmed “bicontinuous” 3D LC phases, the nonchiral “double-gyroid” cubic (spacegroup *Ia*3̅*d*) (Figure 1e),^14^ the chiral tetragonal “Smectic-Q” phase (*I*4_1_22) (Figure 1f),^15,16^ and the chiral triple-network cubic (*I*23) (Figure 1g).^17,18^ The branched networks in these 3D phases, as well as the infinite parallel columns in the columnar phase of polycatenars, are made up of 2–3 parallel or antiparallel molecules lying side-by-side forming “rafts” (see Figure 1b). These, in turn, stack on top of each other. Following the relatively recent discovery that the triple-network cubic is always chiral^19^ and that the chiral “Smectic-Q” is also a bicontinuous network phase,^16^ it has been realized that what gives chirality to these phases occurring in achiral compounds is the same-handed twist between successive rafts (see Figure 1c).^18,19,16^ The relatively small twist (8–10°) between rafts is the result of a balance between attractive forces between aromatic rods, favoring parallel stacking, and the repulsion between the bulky end chains favoring the twist.^20,21^ Moreover, to explain the long-range propagation of persistent helical sense leading to macroscopic chirality, it was proposed that efficient space-filling at network junctions dictates that all 3 or 4 columnar segments joined at the junction must have the same twist sense (Figure 1d). In analogy with spontaneously ordered ferromagnets and ferroelectrics, such ordering in LCs can be considered as ferrochirality.,^20,22^ In the case of the double gyroid, its lack of net chirality is attributed to the cancellation of the two antichiral constituent networks, hence to its antiferrochirality.

Regarding columnar phases, they appear in many different systems with widely varying molecular architecture. Originally they were discovered in disklike aromatic molecules,^23^ but their type ranges from self-assembled biomimetic virus-like assemblies^24^ to apparently unlikely LC-forming candidates such as polyethylene and Teflon.^25−27^ Columnar phases of many π-conjugated compounds are one-dimensional (1D) semiconductors.^28−32^ They can be used, e.g., in light harvesting and light emission, sensors, ionic conductors, etc.^33^ Combining these features with chirality could enhance their versatility as functional materials, e.g., as emitters of circularly polarized light.^34^

Unlike the cubic and related 3D network phases, the columnar phase has no junctions. Accordingly, no long-range homochirality has been reported in columnar LCs of achiral polycatenars. One may argue that this is consistent with the general physical principle that no long-range order (LRO) can be maintained in isolated 1D chains,^35,36^ be they chains of spins or of twisted molecular rafts. There have been some reports of helical columnar phases in achiral compounds with molecules that were not rodlike but were disk- or bowl-like in shape, but their macroscopic chirality is unconfirmed.^37^ Even more of such reports have actually described soft crystals rather than liquid crystals.^38,39^ Since in crystals there is direct pairwise interaction between molecules with defined periodically repeating positions on neighboring columns, homochirality propagation is understandable. Another aspect of chirality propagation and amplification is the full or partial deracemization of a mixture of enantiomers. Its recognition in “hard” crystals goes back to Pasteur.^40^ In “soft” or “condis” crystals, where not all atoms of the molecule have a fixed position on the lattice (e.g., crystals containing “molten” flexible pendant chains), intermolecular potential between columns, although smeared, is still periodic as at least the centers of gravity of the molecules are on a 3D lattice. There have indeed been a number of reports of deracemization in soft columnar crystals,^41,42^ although it is not clear whether the enantiomers were separated in different crystals or in different columns of the same crystal. In true LCs, the positions of individual molecules are not defined. Especially for columns of a nearly circular cross section, it is not clear how the twist sense would be communicated between two straight cylindrical columns isolated from each other by a sheath of molten chains. They are expected to act as smooth cylinders, and their chirality is not expected to be transferred from column to column. Helix reversal defects are inevitable, and the induction of long-range chirality in achiral compounds forming cylindrical LC columns is not expected.

An additional complication obstructing the detection of chirality in columnar LCs is that optical activity and circular dichroism (CD), the two chiro-optical effects usually relied on, are overwhelmingly dominated by linear dichroism and birefringence of these phases. This problem does not arise in the cubic LCs described above since they are optically isotropic. Consequently, the optical activity of columnar phases is, on the whole unexplored. Another difficulty in establishing if a columnar LC phase is helical and if the helix is regular is the fact that diffuse X-ray scattering features, sometimes invoked as evidence of helicity, give ambiguous information. Thus, fiber X-ray diffraction patterns containing distinct layer lines have been cited as indications of regular helicity of columns, even though it has been demonstrated that chain-like structures with irregular twist or even no twist can easily produce such layer lines.^43^

A type of columnar phase where one would expect the column cross section to be noncircular is that in polycatenar compounds with columns of stacked rafts of linear molecules (see Figure 1b). Due to their elliptical or even dumbbell cross section, they could be regarded as ribbons. It is conceivable that close packing of twisted ribbons could possibly transfer chirality information between columns even without molecular crystalline order. Nevertheless, no long-range homochirality has been reported in columnar LCs of linear polycatenars, in spite of the fact that columnar LC phases have been known in these compounds since the 1980s.^1−3^ However, it was found only very recently that at a lower temperature, these compounds can form another, rather complex orthorhombic LC phase with *Fddd* symmetry.^20^ Although consisting of twisted ribbons made up of stacked rafts, there was no net chirality because the number of right- and left-twisted ribbons was equal. This phase was found not only in compounds with straight rods but also in those with a “banana-shaped” core containing a 120° bend in the middle. Three such molecules, packed back-to-back, formed a 3-arm self-assembled star. Like the rafts, these stars also pack in twisted stacks, as in dense packing of left- and right-handed screws. The appearance of the highly ordered yet noncrystalline *Fddd* phase confirmed that the chirality of close-packed twisted *ridged* (i.e., noncircular) columns, without any junctions, can indeed be maintained over a long range. Subsequently, the *Fddd* phase was also found in a compound with an **I**-shape core, one-ring wide in the middle and two-ring wide at each end.^44^ Our preliminary studies also suggest that this phase and its variants may be widespread in polycatenars, including those formed by hydrogen-bonded half-rods and in side-chain LC polymers. It would also not be surprising if it was found in compounds with 3-arm star molecules of *C*_*3*_ symmetry that also form a columnar phase at higher temperatures.^45,46^ As the *Fddd* phase is becoming a major LC type, we engaged in a more systematic study of its features. Furthermore, we undertook to find if rodlike polycatenars with a strong chiral center could produce an ordered chiral LC phase of homochiral ribbons.

In this work, we prepared compounds with a long rodlike core containing a donor–acceptor (D–A)-type thienylfluorenone (TFO) chromophore with 6 tethered alkyl end chains. A chiral center is introduced into the core through a 3-hydroxybutyrate linkage. An analogous nonchiral compound with a 3-hydroxypropionate linkage is also studied, for comparison. The materials are investigated by small- and wide-angle X-ray scattering (SAXS, WAXS), grazing-incidence SAXS and WAXS (GISAXS, GIWAXS), differential scanning calorimetry (DSC) including modulated (MDSC) and flash DSC (FDSC), polarized optical microscopy (POM), UV–vis and fluorescence spectroscopy, Mueller matrix chiro-optical spectroscopy, and conformational analysis. We find that pure *R* and *S* enantiomers form an optically active hexagonal columnar phase (Col_h_*) with only short-range *Fddd*-like clusters. However, in an *R*–*S* racemic mixture, a highly ordered *Fddd* phase forms. The enantiomers were found to partially preorder already in the columnar precursor and fully separate into left- and right-handed helical columns in the *Fddd*. Interestingly, the equivalent nonchiral compound stays Col_h_ and does not form the *Fddd* at all. An explanation of the observed phenomena is proposed based on conformational analysis. Other findings include the remarkable ability of the Col_h_ phase to form at cooling rates as high as 15,000 K/min and of deracemization into separate columns at >50 K/min. A distinction is discussed between the spontaneous long-range ordered chirality induced by the 3D lattice (“ferro- and antiferrochirality) and the irregular helicity of twisted columns where the bias toward one twist sense is imposed by the presence of chiral groups (“parachirality”).

## 2 Materials

**Figure 2:**
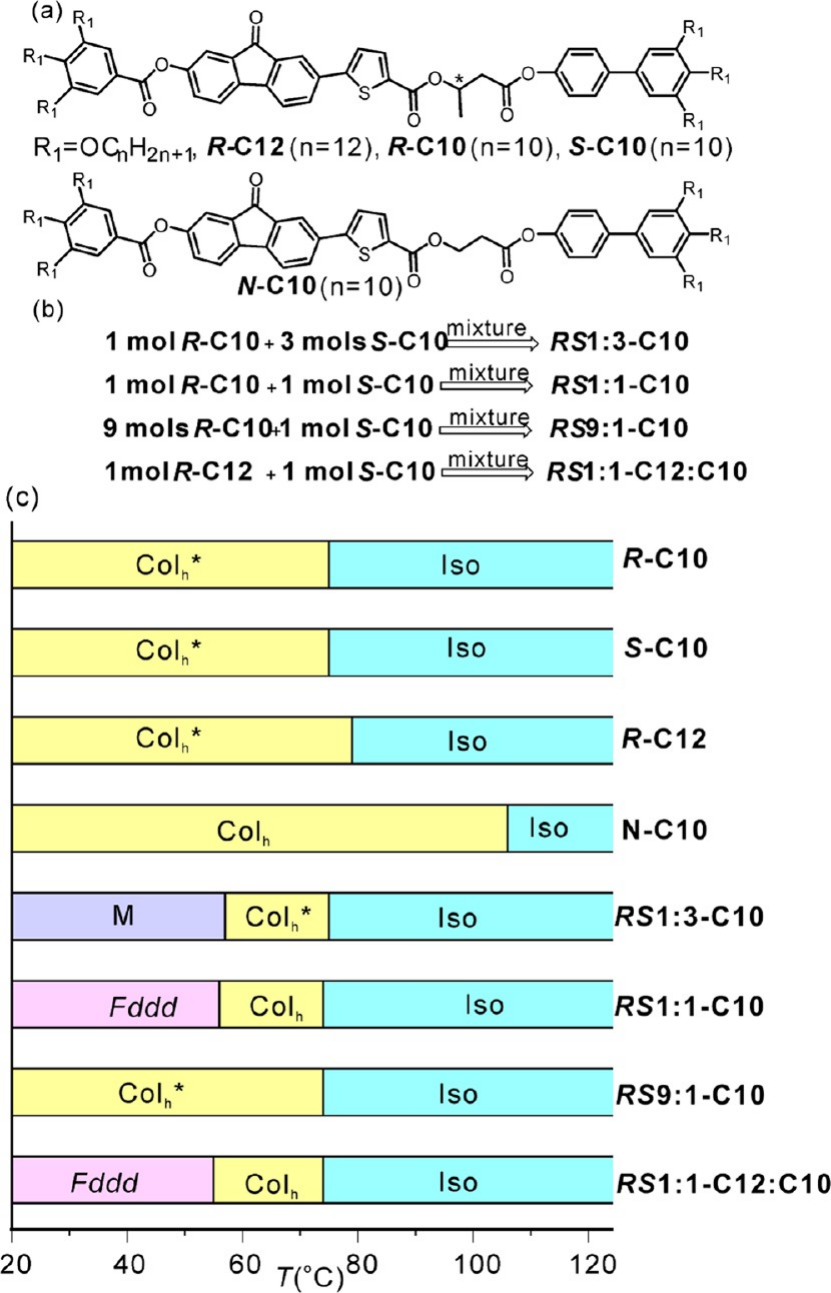
(a) Chemical structure of the compounds. (b) Definition of names of mixtures. (c) Bar chart of phase transition temperatures of the compounds and their mixtures determined on cooling.

The compounds and their mixtures are listed in Figure 2a. The compounds are labeled ***R*****-C*n***, ***S*****-C10**, and **N–C10** where R, S, and N refer to the linking group between the thienylfluorenone and the biphenyl parts of the mesogen, i.e., to 3-*R*-hydroxybutanoate, 3-*S*-hydroxybutanoate, and the nonchiral 3-hydroxypropionate, respectively. *n* = 10 and 12 are the numbers of carbons in the six terminal alkyl chains. The mixtures are labeled ***RS*****p:q-C10**, where p:q is the ratio of the two enantiomers. Figure 2b shows the mesophase temperature ranges of all of the compounds and their mixtures.

The reason for choosing an asymmetric core instead of a synthetically more symmetric one is to lower the crystal melting point. Namely, while in the crystal the molecular orientation would be defined, in the LC they would orient randomly along their long axis, raising the entropy of the LC by *R* ln2 and thus lowering the melting point from Δ*H*_m_/Δ*S*_m_ to Δ*H*_m_/(Δ*S*_m_ + *R* ln2). Here, Δ*H*_m_ and Δ*S*_m_ are the melting enthalpy and entropy, respectively.

**Scheme 1:**
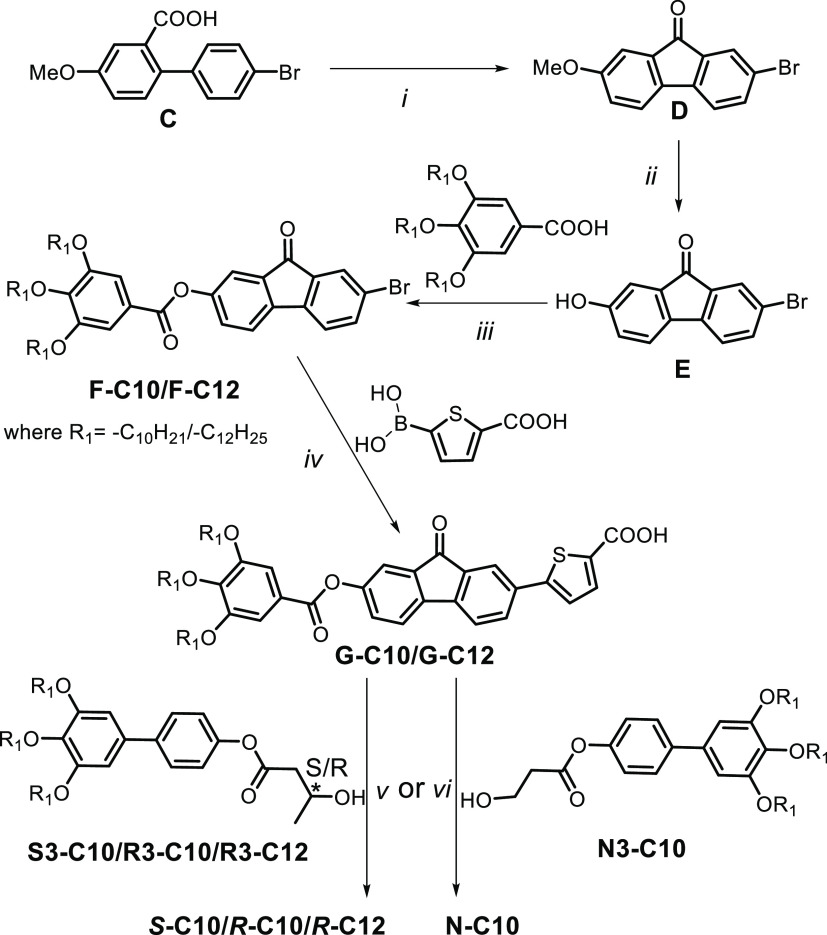
Synthesis of Compounds ***S*****-C10**, ***R*****-C*n*** (*n* = 10, 12), and **N–C10** Reagents and conditions: (i) SOCl_2_, AlCl_3_, DCM, r.t.; (ii) 48% HBr, AcOH, 120 °C; (iii) EDC·HCl, DMAP, DCM. r.t; (iv) Pd(PPh_3_)_2_Cl_2_, THF, 2 M Na_2_CO_3_, 80 °C; (v, vi) SOCl_2_, pyridine, DCM, r.t.

The ***S*****-C10**, ***R*****-C*n*** (*n* = 10 and 12), and **N–C10** compounds were achieved as described in Scheme 1. The first step is a Friedel–Crafts reaction of ring closure of 4′-bromo-4-methoxy-[1,1′-biphenyl]-2-carboxylic acid **C** to give the 2-bromo-7-methoxy-9*H*-fluoren-9-one **D**. The second step is a demethylation reaction under strongly acidic conditions with the formation of compound 2-bromo-7-hydroxy-9*H*-fluoren-9-one **E**. The compounds **F** were obtained by an esterification reaction between **E** and the corresponding tris(alkoxy)benzoic acid in mild condition. Then, by a Suzuki coupling reaction, **F** and 2-carboxythiophene-5-boronic acid were reacted to give 5-(9-oxo-7-((3,4,5-tris(alkoxy)benzoyl)oxy)-9*H*-fluoren-2-yl)thiophene-2-carboxylic acid **G-C10/C12**.

The intermediates 3′,4′,5′-tris(alkoxy)-[1,1′-biphenyl]-4-yl (*S*/*R*)-3-hydroxybutanoate **S3–C10/R3-C*n*** (*n* = 10 or 12) and 3′,4′,5′-tris(decyloxy)-[1,1′-biphenyl]-4-yl 3-hydroxypropanoate **N3** were obtained by the etherification reaction of [1,1′-biphenyl]-4-ol and commercial (***S***)/(***R***)-3-hydroxybutanoic acid or 3-hydroxypropanoic acid. To get the targeted compounds, the G compounds were transformed in acid chlorides and these by an esterification reaction with **S3–C10, R3-C*n*** (*n* = 10 or 12), or **N3–C10**, respectively, lead to the ***S*****-C10**, ***R*****-C*n*** (*n* = 10 and 12) and **N–C10** compounds.

All final products were purified by silica gel column chromatography and characterized by 1D and 2D NMR, mass spectrometry, and elemental analysis. Details of the syntheses and characterization of the compounds can be found in the Supporting Information.

## 3 Results and Discussion

### 3.1 Basic Phase Behavior and Structure

**Figure 3:**
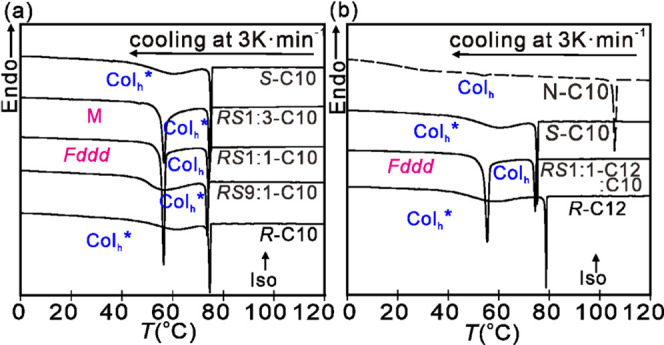
(a, b) Cooling DSC thermograms of compounds and mixtures: (a) ***R*****-C10**, ***RS*****9:1-C10**, ***RS*****1:1-C10**, ***RS*****1:3-C10**, and ***S*****-C10**; (b) ***R*****-C12**, ***RS*****1:1-C12:C10**, ***S*****-C10**, and ***N*****-C10**. The cooling rate in both panels (a) and (b) is 3 K min^–1^.

Conventional cooling DSC thermograms of all compounds and their mixtures listed in Figure 2 are shown in Figure 3. First and second heating thermograms are shown in Figure S1 in the SI, and the transition temperatures and enthalpies are listed in Table 1. The thermograms of pure enantiomers ***R*****-C10** and ***S-*****C10** are seen in Figure 2a to contain one sharp exotherm and a broad exothermic hump at lower temperatures, the reverse occurring on heating (Figure S1). The typical POM texture of the hexagonal columnar phase (Col_h_) is observed both at 70 °C and at 30 °C with no visible change, as shown in Figure 3. Remarkably, in contrast to pure enantiomers, their racemic mixture shows a second sharp exotherm below the Iso-Col_h_ transition (Iso = isotropic), with a relatively high transition enthalpy of 8 J/g on cooling and 10 J/g on heating; see Table 1. Not only the 1:1 mixture but even a 1:3 mixture of enantiomers displays the sharp transition to a low-T phase (Figure 3a), with a transition enthalpy close to that of the racemate (Table 1). The sharp transition is lost when the fraction of the counter-enantiomer drops to 10%, leaving only a broad heat capacity maximum as in the pure enantiomers.

**Table 1:** Transition Temperatures, Lattice Parameters, and Number of Molecules Per Column Raft

compd/mixture	*T*/°C [Δ*H*/J g^–1^]^b^	lattice parameters (nm)^a^
***R*****-C10**	H^1st^: Cr 78 [22.0] Iso	*a*_h_ = 4.56
	C: Iso 75 [2.6] Col_h_*	
	H^2nd^: Col_h_* 76 [2.3] Iso	
***S*****-C10**	H^1st^: Cr78 [20.5] Iso	*a*_h_ = 4.53
	C: Iso 75 [2.6] Col_h_*	
	H^2nd^: Col_h_* 77 [2.3] Iso	
***R*****-C12**	H^1st^: Cr 78 [19.6] Iso	*a*_h_ = 4.76
	C: Iso 79 [2.2] Col_h_*	
	H^2nd^: Col_h_* 80 [2.1] Iso	
**N–C10**	H^1st^: Cr 63 [13.1] Col_h_108[2.3]Iso	*a*_h_ = 4.60
	C: Iso 106 [2.2] Col_h_	
	H^2nd^: Col_h_ 108 [2.2] Iso	
***RS***1:3**-C10**	H^1st^: M 62 [8.1] Col_h_* 77 [2.3] Iso	*a*_h_ = 4.54
	C: Iso 75 [2.4] Col_h_* 57 [5.4] M	
***RS***1:1**-C10**	H^1st^: *Fddd* 64 [10.3] Col_h_ 77 [2.3] Iso	*a*_h_ = 4.54
		*a*_orth_ = 16.04
		*b*_orth_ = 9.26
	C: Iso 74 [2.2] Col_h_ 56 [7.6] *Fddd*	*c*_orth_ = 3.77
***RS***9:1**-C10**	H^1st^: Cr 76 [16.7] Iso	*a*_h_ = 4.55
	C: Iso 74 [2.5] Col_h_*	
	H^2nd^: Col_h_* 76 [2.3] Iso	
***RS***1:1**-C12:C10**	H^1st^: *Fddd* 63 [10.1] Col_h_ 77 [2.0] Iso	*a*_h_ = 4.70
		*a*_orth_ = 16.52
	C: Iso 74 [2.1] Col_h_ 55 [7.7] *Fddd*	*b*_orth_ = 9.54
		*c*_orth_ = 3.78

a Lattice parameters of hexagonal *a*_h_ and orthorhombic *Fddd* phase *a*_orth_, *b*_orth_, and *c*_orth_ (see Supplementary Tables 1–6).

b Peak DSC transition temperatures and enthalpies at 3 K·min^–1^ for all samples. Cr = crystal, Col_h_ = hexagonal columnar, Iso = isotropic. melt, H = heating, C = cooling. For more DSC data, see Figures 3 and S1.

The enantiomer with somewhat longer alkyl chains, ***R*****-C12**, is seen in Figure 3b to behave in a very similar way to that of its C10 homologue. Furthermore, the thermogram of a 1:1 mixture of ***R*****-C12** and ***S*****-C10** is very similar to that of the racemate of the two C10 enantiomers; even the transition enthalpy is undiminished, showing that the low-T phase is tolerant of variation in both molecular size and ratio of the enantiomers as long as they are mixed and not pure.

The absence of the low-temperature phase and a phase transition in pure enantiomers and their presence in mixtures are unusual and may be counterintuitive. Ordered low-*T* phases are usually seen in pure compounds and their absence is more likely in mixtures. The opposite behavior observed here will be discussed in detail.

**Figure 4:**
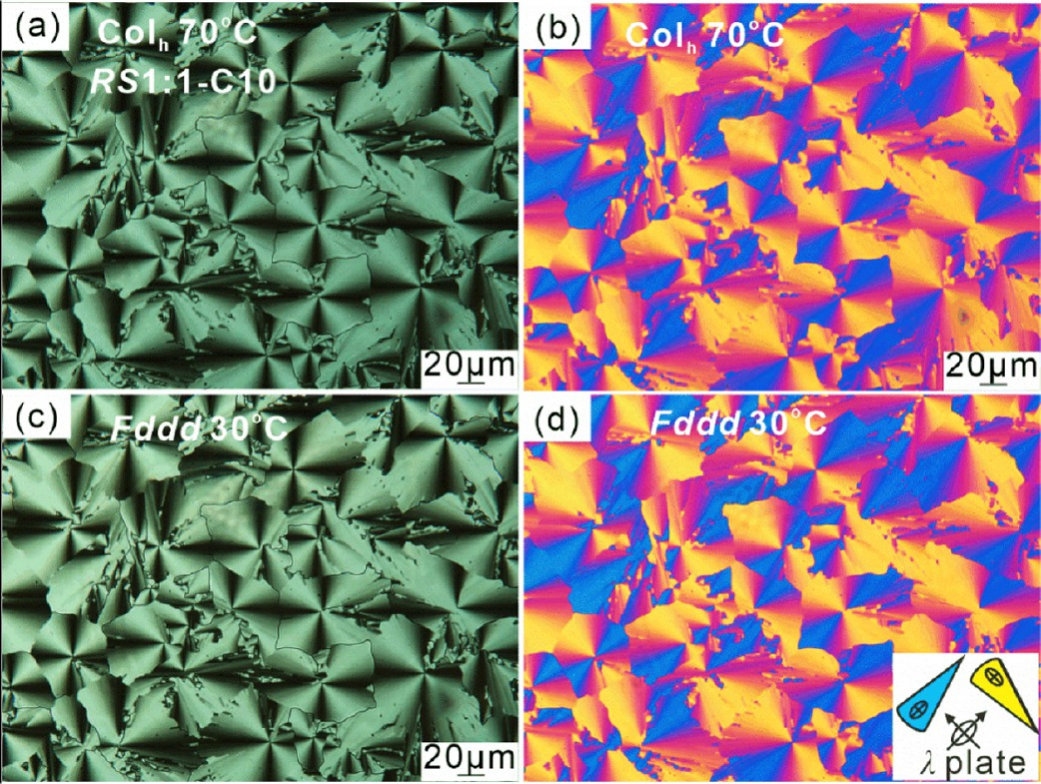
Polarized optical micrographs of ***RS*****1:1-C10**. (a, b) Col_h_ phase at 70 °C; (c, d) *Fddd* phase at 30 °C; (b) and (d) are recorded with a full-wave (λ) plate. The scale bar in (b) applies to all panels. The inset in (d) shows the orientation of indications of the λ-plate and of the colored fans. See more textures in Figures S3–S5.

The circular or fan-like birefringent regions in POM images in Figure 4a,c suggest that these are the cylindrical developable domains,^47^ often referred to as “spherulitic” patterns, characteristic of Col_h_. The images taken with the λ-plate (Figure 4b,d) show that the high-index axis is radial in the developable domains, and since the columns are normally tangential, the high-index direction is perpendicular to the column axis. This is consistent with the columns being negatively birefringent with the average orientation of the π-conjugated rods normal to the column axis. Notably, in the racemic mixture the image of the low-T phase (Figure 4c) is somewhat brighter than that of the high-T phase, indicating that the low-T phase is slightly more negatively birefringent (see also Figure 7a and the discussion below).

**Figure 5:**
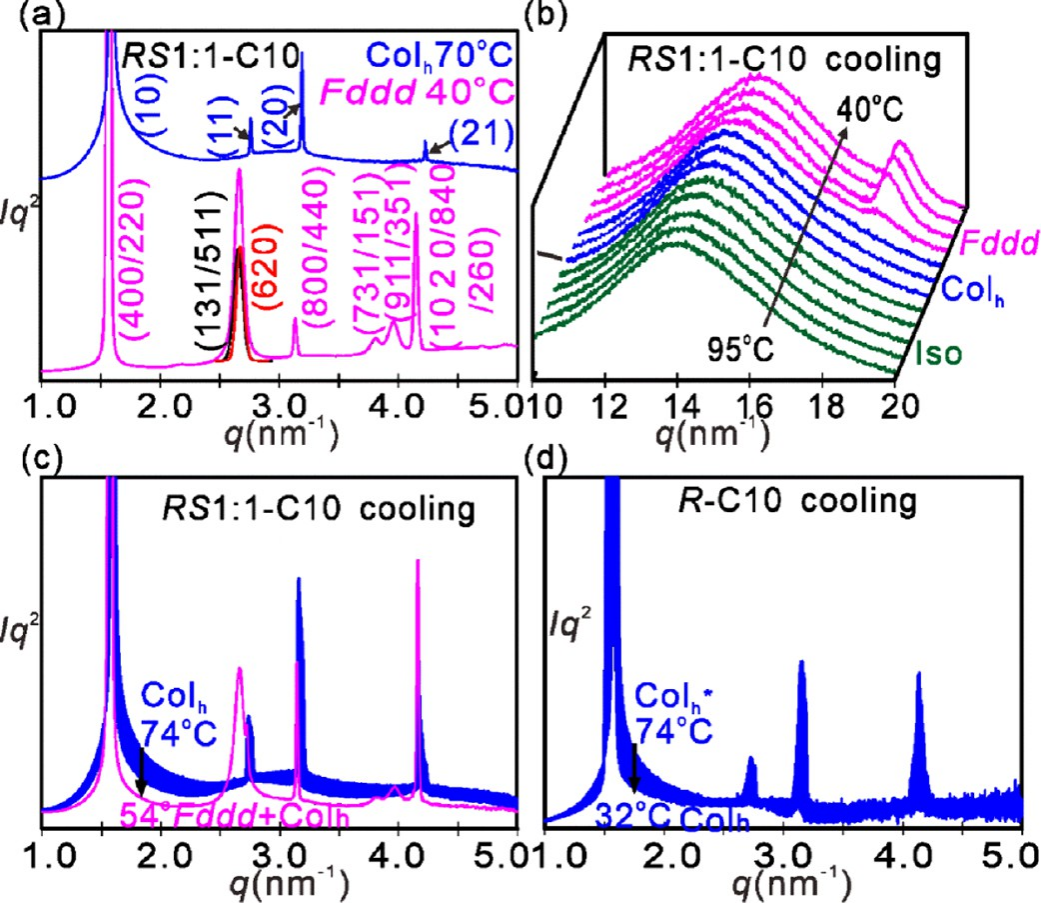
(a) Transmission powder SAXS curve of the high-T Col_h_ phase (75 °C, blue) and the low-T *Fddd* phase (45 °C, magenta) of the ***RS*****1:1-C10** mixture. The red and black peaks in the *Fddd* diffractogram are overlapping components separated on the basis of corrected intensities of separate GISAXS spots. (b) Sequence of WAXS profiles recorded during cooling through the Iso → Col_h_ → *Fddd* phase sequence of ***RS*****1:1-C10**. (c, d) Evolution of the SAXS curve on cooling through the *T*-range of the Col_h_ phase of (c) ***RS*****1:1-C10** mixture and (d) ***R*****-C10** enantiomer. Note the pretransitional diffuse scattering around *q* = 2.7–2.8 nm^–1^ increasing on approach the *Fddd*.

**Figure 6:**
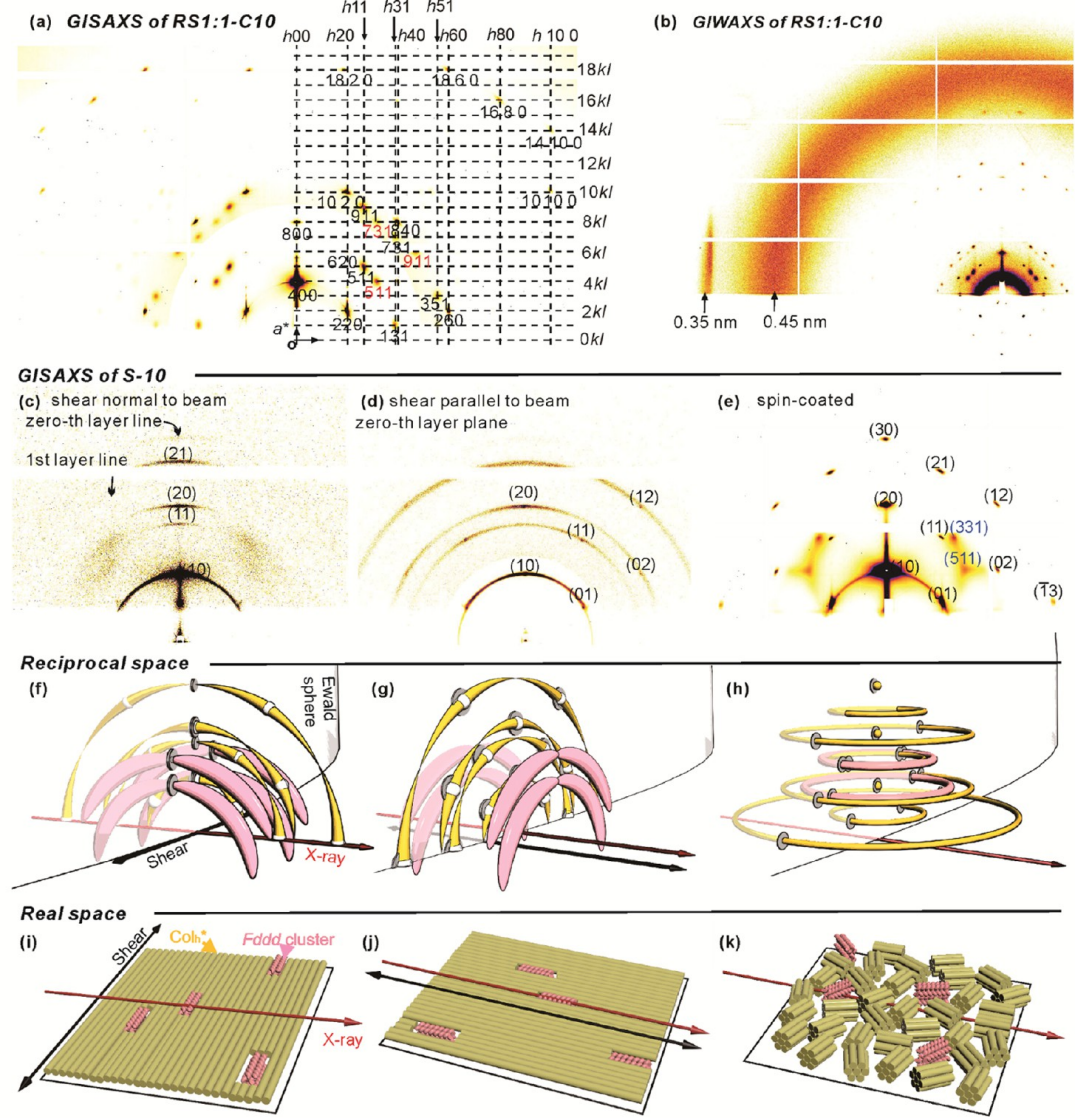
(a) GISAXS and (b) GIWAXS patterns of a thin horizontal film of the ***RS*****1:1-C10** mixture in the *Fddd* phase recorded at 50 °C. A reciprocal net for the main fiber-like orientation randomized around the vertical [100] axis is superimposed on the right in (a). Spots with Miller indices in red are from a minority [110] orientation. Note the 0.35 nm vertical streak in (b) due to π–π stacking along the (horizontal) columns. (c, e) GISAXS of ***S*****-C10** recorded at 25 °C. (c, d) A sheared film on Si substrate; shear direction horizontal, in (c) perpendicular and in (d) parallel to the beam. (e) Spin-coated film on Si. (*hk*) indices mark Bragg reflections of Col_h_ phase, and (*hkl*) indices mark the broad spots of small *Fddd* clusters. For intensity profiles, see Figure S7. (f–h) Reciprocal space representations of diffraction geometry in (c–e). The yellow arcs in (f) and (g) are generated from ideal *hk*0 points on the zeroth-layer plane of the reciprocal lattice (small white spheres) by smearing them azimuthally about the shear axis due to imperfect orientation. The pink arcs are equally smeared diffuse points on the first-layer plane (*hk*1) coming from small *Fddd* clusters in the enantiomer, giving rise to two diffuse blobs on each side of the central vertical line in (c). Diffraction maxima are seen wherever a reciprocal lattice point or arc touches the Ewald sphere since only these satisfy the Bragg equation. (h) Reciprocal lattice points are fully randomized about the normal to the Si substrate in the spin-coated sample, each point describing a full circle, with the Ewald sphere crossings indicated by a small white circle. Panels (f)–(h) should be compared to diffraction patterns in (c–e) and to the real-space models of the columns in (i–k). *Fddd* clusters in (i–k) are colored pink. In (k), columns are randomly oriented in the film plane.

Powder SAXS diffractograms of the high- and low-T phases of the ***RS*****1:1-C10** mixture are shown in Figure 5a. The diffractogram of the high-T Col_h_ phase is easily recognized by the Bragg peaks with *q*-values in the ratio 1:√3:2:√7··· With the help of the GISAXS pattern on oriented thin film in Figure 6a, the Bragg peaks of the low-T phase in Figure 5a could be indexed accurately on a 3D orthorhombic lattice with spacegroup *Fddd*. The list of observed and calculated *d*-spacings is presented in Table S2. Although diffractograms of this phase have already been reported,^20^ their characteristic features may not be recognized immediately since the aspect ratio of the unit cell can vary substantially. Temperature variation of the wide-angle diffractogram is shown in Figure 5b. Note the 0.35 nm peak (*q* = 18 nm^–1^) in the *Fddd* phase.

The evolution of the powder SAXS profile of the ***RS*****1:1-C10** mixture and the ***R*****-C10** enantiomer on cooling through the Col_h_ phase is shown in Figure 5c,d. They will be discussed further below. Observed and calculated spacings, diffraction intensities, and structure factor phases of the LC phases in different compounds and mixtures are listed in Tables S1–S6 in the SI.

Figure 6a is the GISAXS pattern of a thin spin-coated and annealed film of the ***RS*****1:1-C10** racemate. The *Fddd* phase in the film is highly aligned with its *x*-axis (*a*-axis) normal to the film plane (vertical), while the *y*- and *z*-axes are randomized in-plane (compare with Figure 6k). Superimposed on the GISAXS pattern is the reciprocal lattice net. In fact, the pattern is a supposition of two orientations; the net is shown only for the dominant orientation in which the LC sits with its (100) plane, one of its densely packed plane, on the Si substrate. Bragg reflections belonging to the alternative (110) orientation are labeled in red. The (110) plane is another densely packed plane of columns. The systematic extinctions observed define the *Fddd* spacegroup unambiguously.

For an explanation of the geometry of diffraction, showing schematically the reciprocal space and Ewald sphere, see Figure 6f–k and the accompanying figure caption.

Figure 6b shows the GIWAXS pattern of the *Fddd* phase. Note the vertical streak on the left at 2π/*q*_*xy*_ = 0.35 nm, indicating close π–π stacking along the (horizontal) column axis of the TFO plates; for a more detailed discussion, see Section 3.5. The 0.35 nm peak disappears in the Col_h_ phase (Figure 5b), and it is not present in either the pure enantiomers or in **N*****-*****C10** (Figure S13).

In contrast to the mixture, the only sharp Bragg reflections in the GISAXS patterns of the pure enantiomer (Figure 6c–e), either above or below the broad *C*_*p*_ hump, are those of the 2D hexagonal lattice. Similarly, powder SAXS of all enantiomers, i.e., **R-C10**, **S–C10**, and **R-C12**, also shows only Bragg reflections of the Col_h_ phase (Figures 5d, S6, and S9). There are however diffuse scattering features to be discussed in Section 3.2.

**Figure 7:**
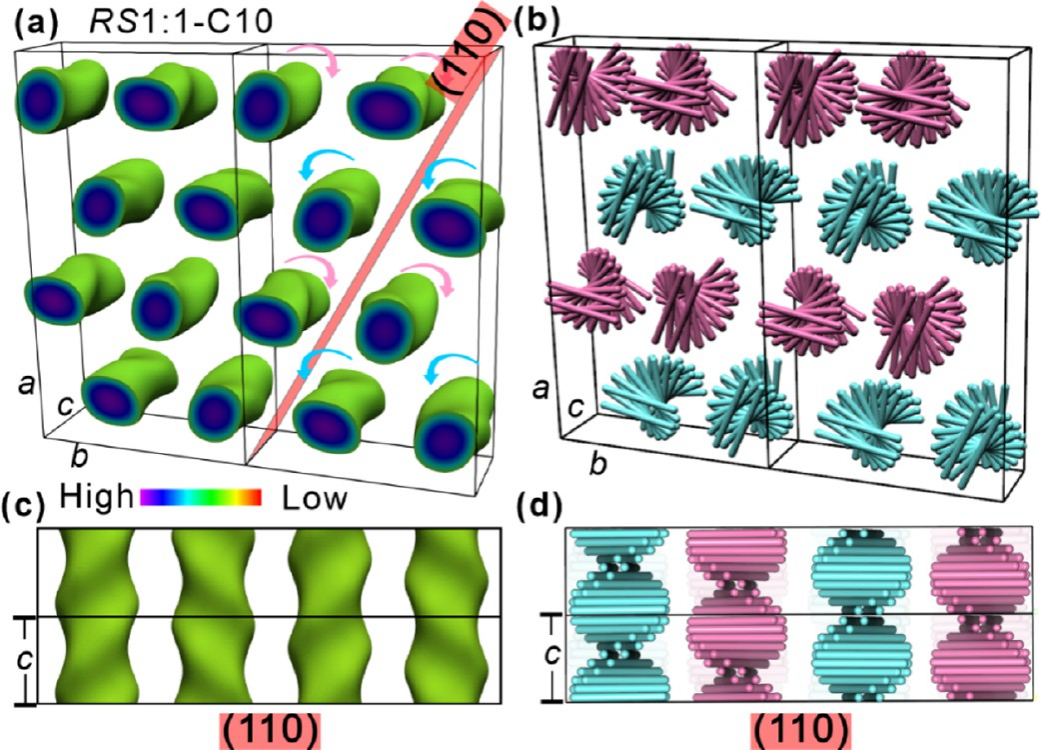
(a–d) ED maps and stylized models of the *Fddd* phase of ***RS*****1:1-C10**. (a, c) 3D electron density maps with high-ED regions (aromatic) enclosed within the isoelectron surface. (b, d) Schematic models of winding rodlike molecular cores. Blue and pink colors represent right- and left-handed columns, respectively.

The quality of the diffraction data allowed reconstruction of the electron density (ED) map of the *Fddd* phase of the ***RS*****1:1-C10** blend. To obtain the required reliable diffraction intensities from the powder SAXS pattern the overlapping reflections (131/511) and (620) were resolved numerically, with their relative intensity contributions determined from the GISAXS pattern. The map showing the high-ED aromatic regions is shown in Figure 7a,c. In agreement with the previous model,^20^ one can see 8 columns passing through a unit cell, their axes disposed on a hexagonal net. However, the symmetry is orthorhombic due to the regular twist of the ribbon-like columns. As seen from the map, of the 8 columns, 4 are right-handed and 4 are left-handed. Along the two cell diagonals (110 planes), the alternating columns are antichiral (see Figure 7c), but along the (100) planes, they are homochiral. In addition to having different twist senses, the different columns are also shifted relative to each other along their long axis.

Consistent with their negative birefringence (Figure 4b,d), the 0.35 nm intracolumnar periodicity, the unit cell volume, and the density (Table S7), the molecules are arranged in rafts normal to column axis, with 2.3 molecules on average lying side-by-side in a raft. The steric clash between the out-fanning alkyl end chains causes the rafts to twist relative to their neighbors by an angle φ. Since the half-pitch of the twisted ribbons, i.e., a 180° turn, is equal to *c* = 3.77 nm, there are 11 rafts in a half-turn with φ = 17°. This is similar to φ in linear hexacatenar compounds in ref (20), but larger than in the hexacatenar bent-core compounds where the stratum of the column was not raft-like but star-like, with three bent cores packing back-to-back forming a 3-arm star.

The *Fddd* structure of the current racemic blend, with molecules represented by rods, is schematically shown in Figure 7b,d, where left-handed ribbons are colored pink and right-handed blue. The key difference between the current *Fddd* phase and that reported previously^20,44^ is that by all evidence right- and left-handed columns contain different molecules. In ***RS*****1:1-C10** the two column types contain the two opposite enantiomers of the same basic compound. In the ***RS*****1:1-C12:C10** blend the two components differ also in alkyl chain length; for diffraction data on that blend, see Figures S10– S12 and Tables 1 and S4. Strictly, in the ***RS*****1:1-C12:C10** blend the *Fddd* symmetry should therefore be broken, but SAXS did not show any extra reflections forbidden by that spacegroup. In contrast, in the ***RS*****1:3-C10** mixture, while the diffractogram appears similar to that of *Fddd*, there are some subtle differences, still to be investigated. For this reason, in Figures 2 and 3 and in Table 1, this phase is labeled M for “mesophase”.

We emphasize that *Fddd* is not a conglomerate. It is a distinct phase, thermodynamically stable at low temperatures and unstable at higher temperatures, and in the present case, it needs both enantiomers within the same unit cell to make it stable. The two antichiral ribbons are part of the structure, arranged in a crystallographically ordered fashion. It can be compared to intermetallic compounds present at certain stoichiometrically defined compositions in binary metal alloys, having an ordered arrangement of the two types of atoms.

### 3.2 Continuous Changes with Temperature and Results of Superslow and Superfast DSC

**Figure 8:**
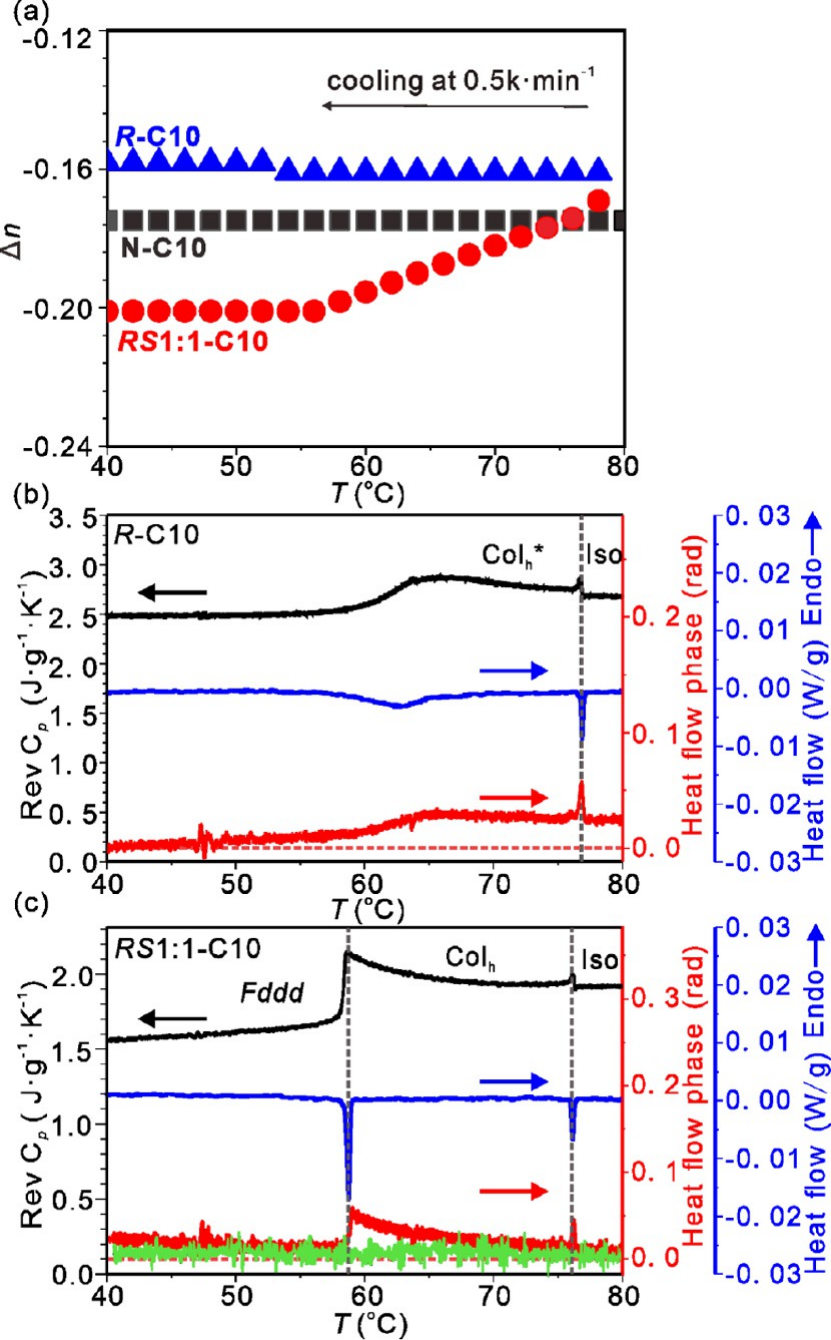
(a) Linear birefringence of compounds ***R*****-C10**, **N–C10**, and the ***RS*****1:1-C10** mixture. (b, c) MDSC traces of (b) ***R*****-C10** and (c) ***RS*****1:1-C10**. In (b, c), the top (black) curve is the reversing heat capacity, the middle (blue) curve is the total heat flow, and the bottom (red) is the phase lag angle of the complex heat capacity. In MDSC experiments, the cooling rate was 0.04 K/min, the modulation amplitude was ±0.07 K, and the modulation period was 20 s. The green trace in (c) was recorded with a modulation period of 60 s. Arrows above the curves point to a relevant scale.

Results of birefringence (Δ*n*) measurements as a function of temperature are shown in Figure 8a. As hinted at already by POM in Figure 4, the negative birefringence of the Col_h_ phase in ***RS*****1:1-C10** becomes progressively even more negative on cooling until around the Col_h_-*Fddd* transition at 59 °C, where it stabilizes. Within experimental error, the pure enantiomer ***R*****-C10** and the achiral **N*****-*****C10** start with the same Δ*n* as ***RS*****1:1-C10** at the top of the columnar *T*-range, but their Δ*n* remains constant on cooling. The most likely cause of the increasingly negative Δ*n* in the racemate is decreasing the tilt of the TFO plates, as they become closer to perpendicular to the column axis. However, as the off-equatorial position of the intensity maximum along the 0.35 nm streak in GIWAXS shows, even in *Fddd*, the TFO plates are still tilted. The tilt in *Fddd* is estimated as arctan(*q*_*xy*_^max^/*q*_*z*_^max^) = arctan(2.4 × 0.35/2π) = 8° (see Figures 6b and S8). The implication from birefringence results is that in the Col_h_ phase, the tilt is relatively high, higher than 8°, and in pure ***R-*****C10** and **N–C10**, it remains so down to room temperature.

The continuous pretransitional changes in the Col_h_ phase of the racemate are indicated even more clearly by the high heat capacity of the columnar phase as measured by modulated DSC (see Figure 8c, black curve). In MDSC a sinusoidal modulation is superimposed on the linear temperature increase/decrease. The black curve shows the reversing part of the *C*_*p*_, In the Col_h_ phase it exceeds that of both the Iso liquid and, particularly, the *Fddd*. We attribute the high and increasing *C*_*p*_ on approaching the first-order Col_h_-*Fddd* transition to the gradual buildup of local *Fddd*-like clusters already in the Col_h_ phase. Note that the blue curve shows the total exothermic heat flow, while the red curve shows the phase angle of the complex heat capacity Δφ, i.e., the lag in heat flow behind the imposed oscillating temperature. In spite of the very slow cooling rate applied (0.04 K/min), the small oscillation amplitude (0.07 K), and the relatively long oscillation period (20 s), the red curve still shows a small but finite phase delay of about 0.04 rad at its peak. However, once the same experiment is repeated with a period of 60 s (green curve), the Δφ maximum disappears completely, showing that on the 1 min scale, the process of building and unbuilding of local *Fddd* clusters is completely reversible.

The buildup of short-range *Fddd*-like clusters in the Col_h_ phase is also evident from powder SAXS where, in Figure 5c, we can see a continuous increase in diffuse scatter around *q* = 2.8 nm^–1^ as temperature decreases toward the Col_h_-*Fddd* transition. At the transition, a large peak appears in the same *q*-range, being a superposition of (131), (511), and (620) Bragg reflections of the *Fddd* phase, cf. Figure 5a. At the same time, the diffuse scatter around the strongest columnar (10) peak at 1.5 nm^–1^ is seen to decrease continuously, indicating a continuous decrease in thermal fluctuations in column position. This diffuse scatter decreases sharply upon the Col_h_-*Fddd* transition but does not disappear completely, *Fddd* being still a liquid crystal.

Interestingly, both conventional and modulated DSC (Figures 3 and 7b) of the pure enantiomer also show a broad *C*_*p*_ maximum at temperatures comparable to that of the high *C*_*p*_ in the racemate. However, the enantiomers by themselves do not show the first-order Col_h_-*Fddd* transition or the associated sharp drop in *C*_*p*_. That is consistent with powder SAXS and GISAXS of the enantiomer, neither of which show evidence of a clear *Fddd* phase (see Figure 5d,c–e). However, consistent with the broad *C*_*p*_ hump around 60–65 °C, a diffuse scattering maximum is seen to develop in powder SAXS of ***R*****-C10** around *q* = 2.7 nm^–1^ (Figure 5d), similar but weaker than that in the racemate. The diffuse X-ray scatter from *Fddd*-like fluctuations in pure enantiomer is clearer in GISAXS. In Figure 6c, ***S*****-C10** had been sheared in the horizontal direction normal to the beam, aligning the columns from left to right. Accordingly, all hexagonal Bragg reflections are on a vertical equatorial line, as explained by the reciprocal space model in Figure 6f. In addition, however, two diffuse blobs are seen on the right and left between the levels of the (10) and (11) columnar peaks. Their *q*_*z*_ position corresponds roughly to the *l* = 1 layer line of the *Fddd* phase, i.e., to the half-pitch of its helical ribbons. In Figure 6d, the shear direction is parallel to the X-ray beam, so only the hexagonal lattice Bragg reflections on the 0-th reciprocal lattice plane are seen as only they touch the Ewald sphere (Figure 6g). But in the fiber-like GISAXS pattern of an unsheared spin-coated film in Figure 6e, where the columns have a random in-plane orientation (Figure 6k), emerging from the diffuse “clouds” on the left and right are weak and broad but distinct spots indexable as (331) and (511) diffraction peaks (pink rings in Figure 6h). We note that these are the strongest reflections of the *Fddd* phase among those that did not emerge from the Col_h_ reflections. A coherence length of *L*_331_ = 33 nm and *L*_511_ = 52 nm can be obtained from the width of these peaks, corresponding to the size of the cybotactic *Fddd*-like clusters in the Col_h_ phase of ***S*****-C10** just below the temperature of the *C*_*p*_ hump. A possible explanation of the reluctant but still noticeable appearance of *Fddd*-like clusters in pure enantiomers is proposed in Section 3.4.

**Figure 9:**
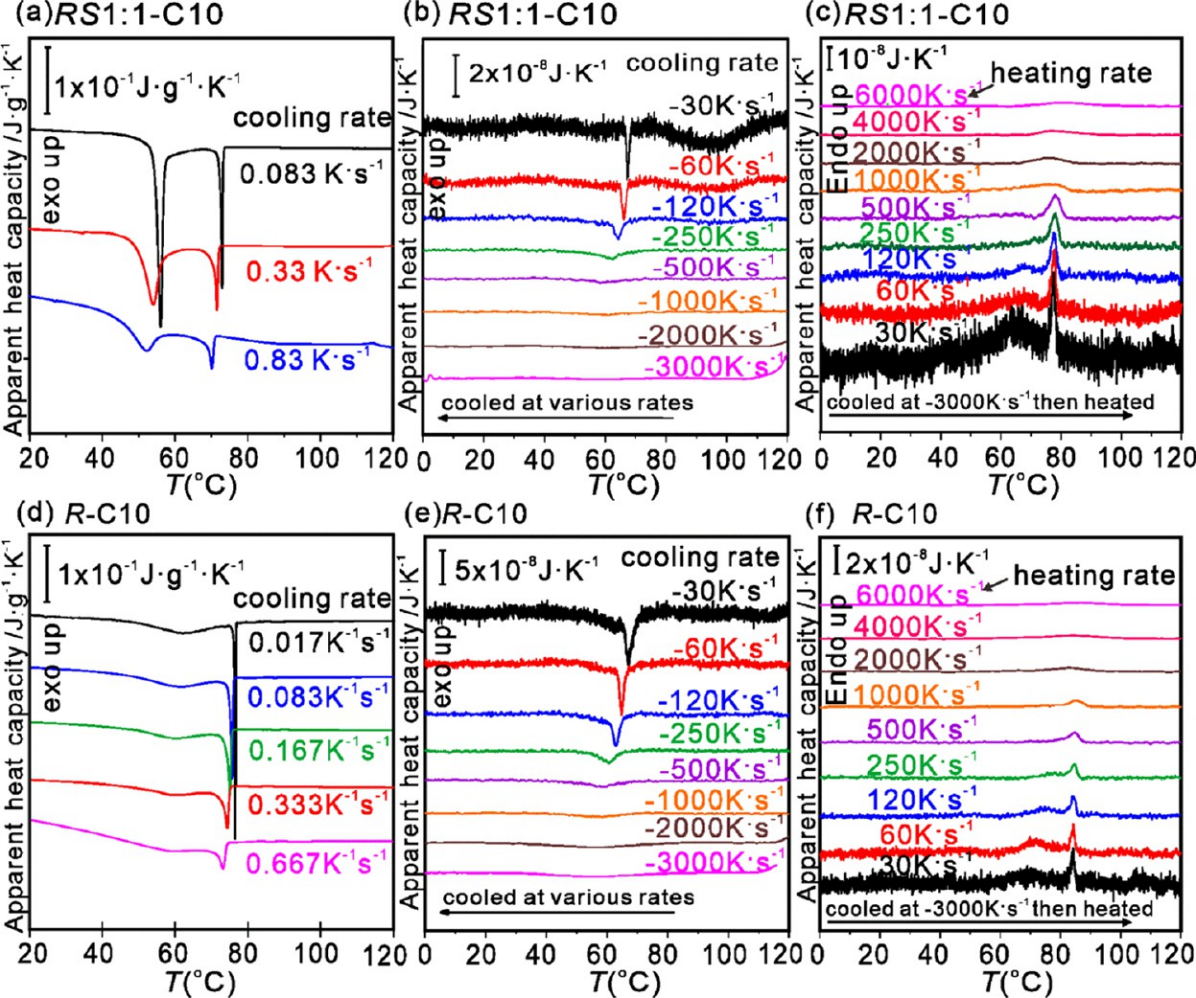
High-speed DSC of (a–c) ***RS*****1:1-C10** racemate and (d–f) ***R*****-C10** enantiomer. (a, d) Conventional DSC cooling scans at increasing cooling rates from Iso liquid: 1 K/min (0.017 K/s), 5 K/min (0.83 K/s), 20 K/min (0.33 K/s), and 50 K/min (0.83 K/s). (b, e) Flash DSC cooling curves at rates from 30 to 3000 K/s. (c, f) FDSC heating curves at rates from 30 to 6000 K/s after cooling at 3000 K/s. Within each panel, heat flow was normalized by dividing by scan rate. (Please do not compress to less than 1.5 column width.)

Although the slow MDSC scans with relatively long modulation periods give us information on equilibrium and the minute time scale dynamics of reaching it, at the other extreme flash DSC tells us about the dynamics down to the ms time scale. Figure 9a shows the effect of a moderate increase in the cooling rate, from 5 to 50 K/min, on the thermogram of the ***RS*****1:1-C10** racemic blend using conventional DSC. Although the Iso-Col_h_ transition remains sharp, the Col_h_-*F*ddd exotherm broadens as the cooling rate increases. Increasing the rate to 1800 K/min or 30 K/s in FDSC (Figure 9b) seems to completely eliminate the Col_h_-*F*ddd exotherm and the *Fddd* phase. However, the Iso-Col_h_ exotherm remains sharp even at 120 K/s (7200 K/min) cooling rate. Its width can be estimated as about 2 K, meaning that the entire transition is nearly complete within 20 ms. This completion time is an upper limit since instrumental broadening was not corrected for. A similar impressive speed of Col_h_ phase formation is seen also in the pure enantiomer (Figure 9e). However, beyond the cooling rate of 1000 K/s the Iso-Col_h_ exotherm is barely noticeable. For comparison, there is a report in the literature about the fast development of a columnar soft-crystal phase, forming at heating/cooling rates up to 1 K/s,^39^ but we are not aware of any reports of columnar phase formation on a ms time scale.

When cooled at 3000 K/s, little or no Col_h_ phase is formed, as evidenced by subsequent heating runs of such a hard-quenched sample shown in Figure 9c,f. Only a small broad endothermic hump is noticeable at reheating rates above 1000–2000 K/min. However, a clear endothermic Col_h_-Iso peak is seen in ***RS*****1:1-C10** at 500 K/s but it seems to be preceded by a small exotherm, suggesting that the Col_h_ phase forms only during the heating run itself. At 120 K/s even a small *Fddd*-Col_h_ endotherm starts appearing, increasing substantially to a broad hump at 30 K/s. Clearly, a poorly ordered *Fddd* phase did form during the heating run at these lower rates. Even though this poorly ordered *Fddd* melts only ∼10 K below the Col_h_-Iso transition, the sharp endotherm of the latter transition is still clear in the lower-rate heating scans in Figure 9c. This implies that the re-racemization, or remixing of the nanosegregated enantiomers to form the Col_h_ phase, took place on a 0.1 s time scale. Even in the pure ***R*****-C10** enantiomer, a smaller broad endothermic hump around 70 °C is visible at the lower heating rates, attributed to melting of the *Fddd*-like clusters formed during the reheating scan (Figure 9f).

It is interesting to compare the kinetics of *Fddd* LC phase formation with that of crystallization of “stereocomplex” in a racemic mixture of polyesters poly-l-lactide and poly-R-lactide. The crystalline stereocomplex contains paired right- and left-handed helical chains. Although it is highly desirable because of its high melting point, the stereocomplex can be grown only very slowly in polymers of acceptably high molecular weight.^48^ Recently its slow growth was explained by an effect named “poisoning by purity”, whereby local nonracemic concentration fluctuations cause the crystal growth surface to be blocked by the rejected surplus enantiomer.^49^ It is conceivable that the relatively slow formation of the *Fddd* phase shown in Figure 9a,b is due to the same diffusion-controlled phenomenon. While the growth of *Fddd* is suppressed at a cooling rate <30 K/s (1800 K/min), PLA stereocomplex stops growing already at 5 K/min. As an aside, we note that the PLA stereocomplex is also not a conglomerate but a thermodynamically stable crystal phase with defined positions of each enantiomer.^50^

### 3.3 Results of Optical and Chiro-Optical Spectroscopy

**Figure 10:**
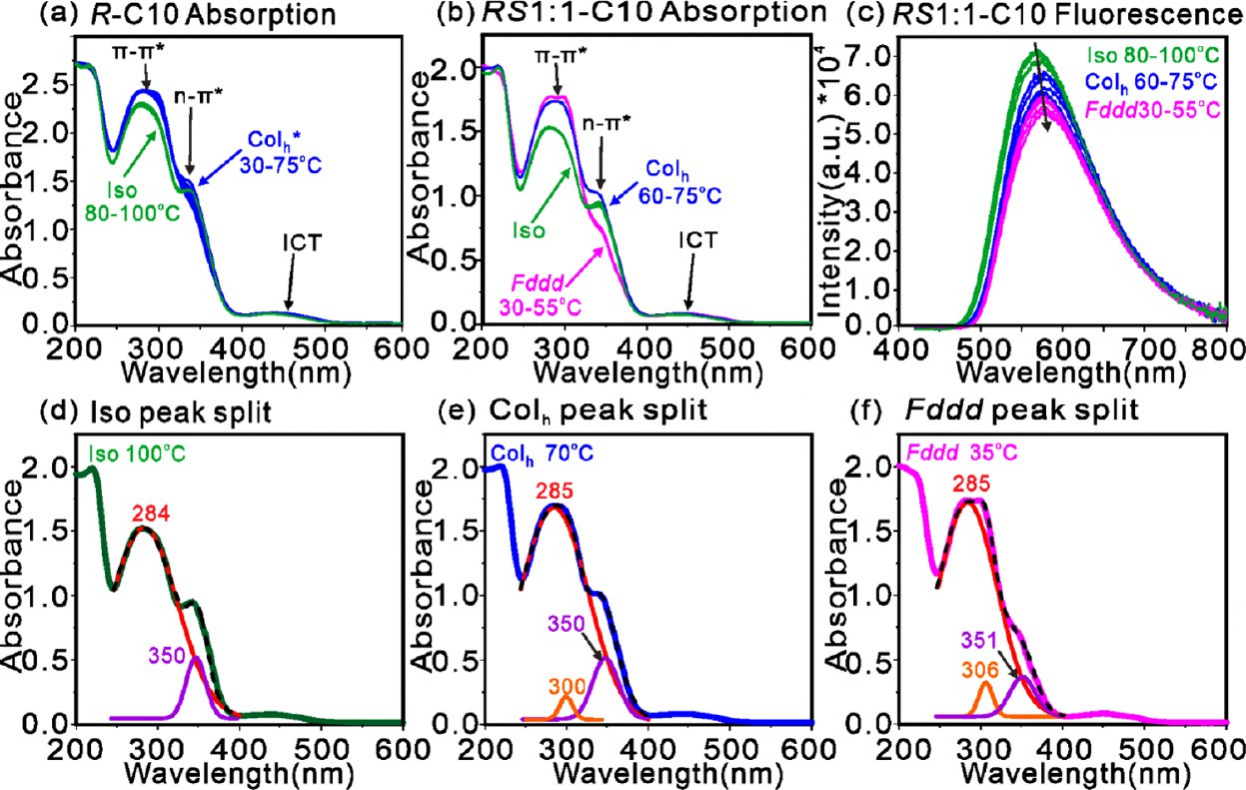
(a, b, d–f) Temperature dependence of UV–vis absorption spectrum of (a) ***R*****-C10** and (b, d–f) ***RS*****1:1-C10**. (c) Temperature dependence of fluorescence emission spectrum of ***RS*****1:1-C10**; excitation 430 nm. (d–f) Peak resolution of the 250–400 nm region of UV–vis spectra of ***RS*****1:1-C10** in Iso, Col_h_, and *Fddd* phases. Wavelengths of the resolved components (red, orange, and purple) are given above the peaks, in nm. The black dashed curve is the sum of the resolved components matching the experimental spectrum.

UV–vis absorption spectra of ***R*****-C10** film vs temperature are displayed in Figure 9a, and those of the ***RS*****1:1-C10** racemate are displayed in Figure 10c. There is consensus that the weak absorption between 400 and 520 nm in mono- and dithienyl fluorenone is due to intramolecular charge transfer (ICT) between the thiophene donor and fluorenone acceptor.^51−53^ In the UV the two most obvious effects of the Iso-Col_h_ and Iso-Col_h_-*Fddd* phase changes are increasing absorbance of the π–π* band around 290 nm and weakening of the n–π* shoulder around 350 nm. The former could be attributed to closer contacts between aromatic groups along the column, facilitating energy transfer and hence depletion of excited states. This is also reflected in the drop in fluorescence emission intensity in Figure 10b. The slight red shift of the fluorescence is also consistent with increasing intermolecular conjugation. The multiplet of absorption bands between 250 and 400 nm in ***RS*****1:1-C10** is curve-resolved in Figure 10d–f. This shows that the apparent near disappearance of the n–π* shoulder near 350 nm on cooling is in part a consequence of moderate weakening of the 350 nm band but is more due to the appearance and strengthening of a band at 303–310 nm. This is probably a spin-off from the 285 nm π–π* band, red-shifted due to improved π–π stacking of primarily the TFO plates, as indicated, e.g., by the appearance of the 0.35 nm diffraction feature (Figures 5b and 6b), the diffuse SAXS around *q* = 2.7 nm^–1^ (Figure 5c,d) and the high heat capacity in the Col_h_ phase (Figures 3 and 8).

**Figure 11:**
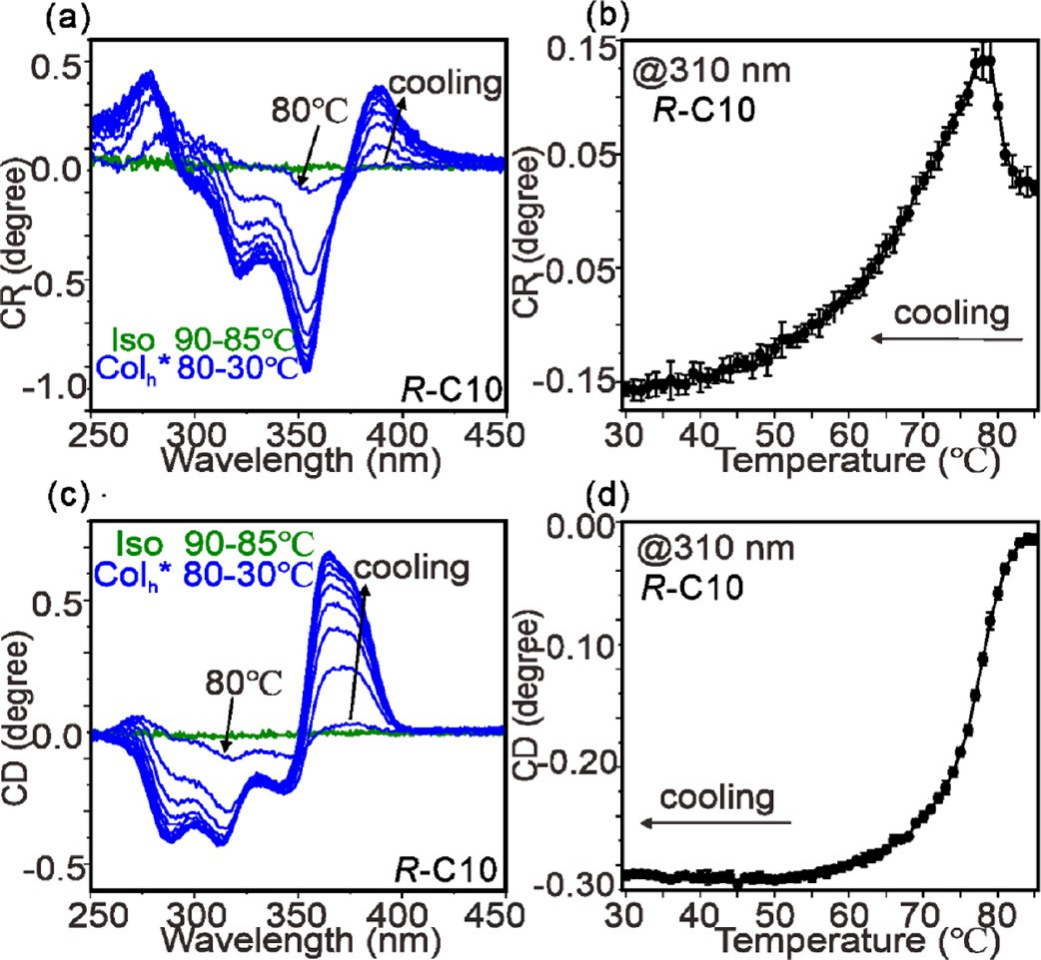
Results of Mueller matrix chiro-optical experiments on ***R*****-C10**. Data were recorded from a film sample during cooling from 90 °C (Iso liquid) to 30 °C through the Col_h_* range. (a) Circular retardance (CR) spectra (proportional to optical rotatory dispersion). (b) CR value at 310 nm vs temperature. (c) Circular dichroism (CD) spectra. (d) CD values at 310 nm vs temperature. In (a) and (c), the spectra were collected isothermally at each temperature, with a 5 K/min cooling rate between the steps. The cooling rate in (b) and (d) was 0.5 K/min.

In order to establish whether self-assembly of chiral enantiomers into Col_h_ phase causes significant chirality amplification,^54^ it would be desirable to measure chiro-optical parameters such as circular dichroism and optical rotation. However, due to the interference of linear birefringence and dichroism of columnar phases, standard techniques cannot be used. However, here we apply the Mueller matrix method^55^ which is capable of separating the effects of linear birefringence and dichroism from those of CD and optical activity. The method uses four photoelastic modulators instead of one as in a standard CD spectrometer, with the ability to measure simultaneously all of the Mueller elements while avoiding errors associated with mechanical or optical rotation.^56^ We applied this technique as implemented on beamline B23 of the Diamond Light Source. The results for ***R*****-C10** enantiomer as a function of temperature on slow cooling are shown in Figure 11. Circular retardance spectra, proportional to the more familiar optical rotatory dispersion, are shown in (a), while the values at 310 nm as a function of *T* are shown in (b). Similarly, circular dichroism spectra are shown in (c) and the values at 310 nm versus *T* in (d). Although there was no detectable CD or optical activity in the Iso liquid, the CD signal developed at the Iso-Col_h_ transition and first increased steeply on cooling through the Col_h_ phase range, then more slowly below 60 °C. Optical activity (CR) jumped up at the Iso-Col_h_ transition, then decreased steeply becoming negative below 70 °C, to continue decreasing more slowly. Such sign reversals are often seen near absorption bands.

As the Col_h_ phase of pure enantiomers is clearly chiral, we may label it Col_h_*. It is noteworthy that the steep increase in chirality, as measured by the above technique, coincides with the temperature region of high heat capacity as measured by MDSC (Figure 8b), as well as with the increased diffuse scattering (Figure 5d) and the appearance of the broad (331) and (511) reflections associated with some local domains of the *Fddd*-like structure (Figure 6e). These comparisons will be discussed in Section 3.5. The shape of the CD spectrum is related to exciton coupling, either positive or negative,^57^ but a detailed analysis is beyond the scope of the current paper.

Due to its large Stokes shift, the TFO fluorophore as well as related fluorenone derivatives are of interest as photoluminescent and electroluminescent emitters. The fact that the columnar phase of pure enantiomers shows strong optical activity means that these materials are also interesting as emitters of circularly polarized light.^58,59^ These are meant to replace the energy-inefficient polarizer/quarter wave plate combination currently used to generate CP light for the removal of reflection from the back plane in displays. On the other hand, preliminary experiments on the *Fddd* phase from current mixtures and related achiral compounds have indicated that the *Fddd* phase provides a long exciton migration path due to its highly ordered close π–π stacked structure, making the materials interesting for photovoltaic applications.^60^

### 3.4 Why Pure Enantiomers and the Nonchiral Compound Do Not Form *Fddd* Phase

**Figure 12:**
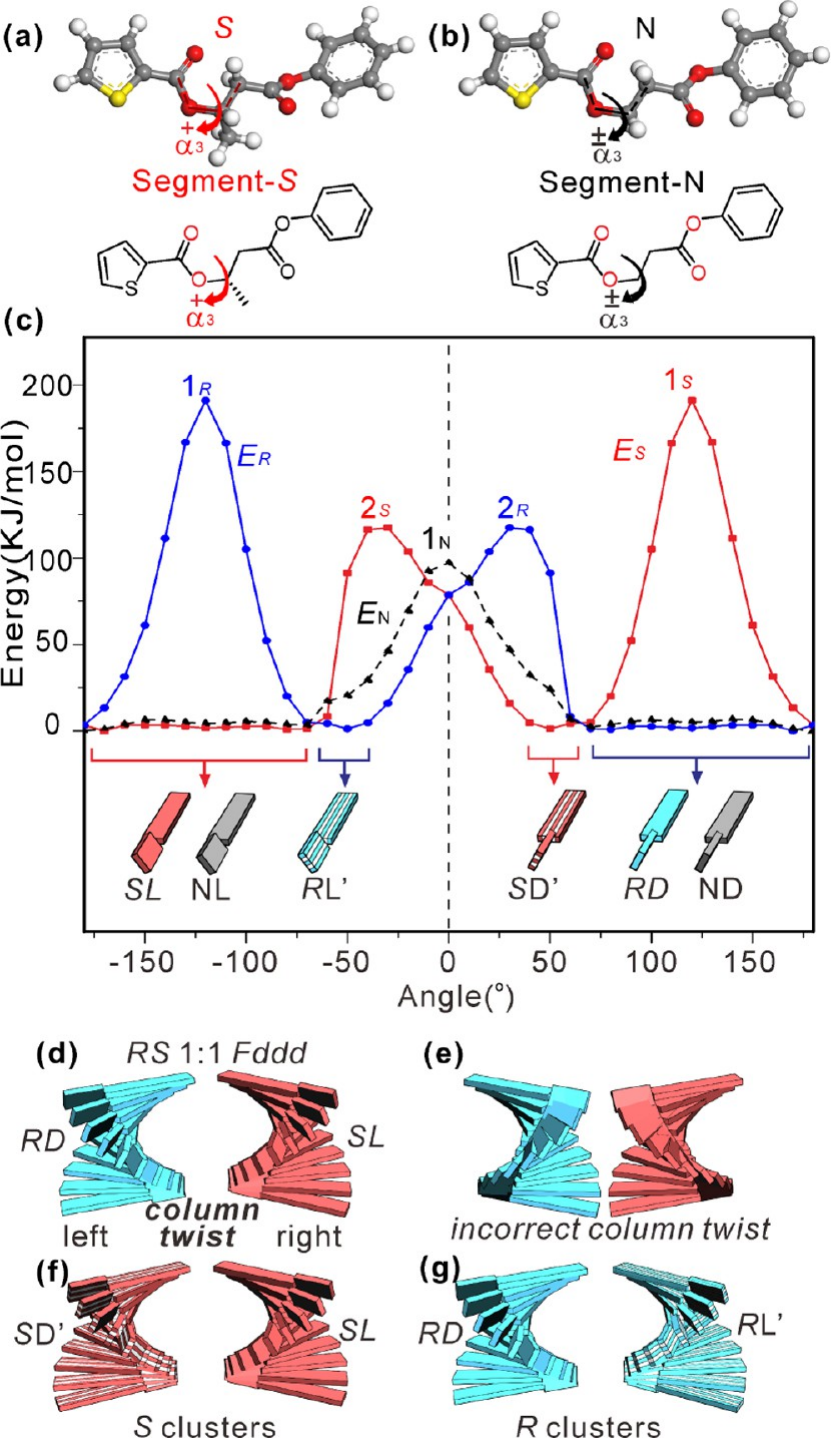
(a, b) Geometry-optimized structure of a molecular segment of (a) ***S*****-C10** and (b) **N*****-*****C10** containing bond 3. Its torsion angle α_3_ was varied in steps of 10°, and potential energy *E*(α_3_) was calculated. (c) *E*(α_3_) for ***R*** (blue), ***S*** (red), and **N** (dashed black) compounds. Stylized mesogenic cores twisted around the α_3_ bond are shown at the bottom of (c). *R* and *S* denote the enantiomer (*N* = N–C10), D = right twist, L = left twist, apostrophe indicates unfavored intramolecular twist sense. (d) Two antichiral columns in *Fddd* phase of an *SD*1:1 mixture, each enantiomer with its favored intramolecular twist, helically twisted in the “correct” direction to maximize aromatic ring overlap: left twist for *R*D and right twist for *S*L molecules. (e) “Incorrectly” twisted columns. (f, g) Part of a local cluster in a pure enantiomer: columns are “correctly” helically twisted, but half of them contain unfavorably twisted molecules. (f) *S* enantiomer; (g) *R* enantiomer.

We now consider the reason why only the ***RS*** mixtures form the *Fddd* phase, while neither the pure enantiomers nor the nonchiral **N–C10** form it. In comparison, all compounds reported so far to form the *Fddd* were achiral.^20,44^ For this purpose, we consider the energy barriers for rotation around single bonds within the mesogen core. Three bonds were examined: (1) the C–C bond between the benzene ring and the ester group in the benzoate, i.e., the rotation angle marked α_1_ in Figure S14, (2) the C–O bond between the ester group and the biphenyl, torsion angle α_2_, and (3) the C–O bond of the butanoate (in chiral enantiomers) or propionate (in the nonchiral **N–C10**), angle α_3_, Figure 12a,b. Rotation around any other bond of the mesogen would compromise its linearity and thus be disfavored by intermolecular interactions and space-filling problems. After each 10° increment in α, the rest of the relevant molecular segment was allowed to relax before the energy was calculated in order to find a realistic minimum-energy path. The energy vs α values for α_1_ and α_2_ are plotted in Figure S14. The energy barriers for those, 21 and 46 kJ/mol, respectively, are not a major concern. Incidentally, a DFT calculation of the barrier to rotation around α_1_ in benzoic acid gave 29 kJ/mol.^61^ The important barriers are those for the third bond, shown in Figure 12c for the relevant segments of enantiomer molecules ***R*****-C*n*** and ***S*****-C10** and for the achiral **N–C10**.

In Figure 12c, the zero angle is defined by the conformation of the models in Figure 12a,b, with the carbonyl oxygen to the left of the α_3_ bond wedged between the two hydrogens on the right.^62^ There are two major barriers to α_3_ rotation in the enantiomers, 125 kJ/mol at ±30° marked 2_R_ (2_S_) and an even higher barrier of 192 kJ/mol at ∓120° marked 1_R_ (1_S_). The two low-energy angular regions between these barriers have similar energies but very different widths. For example, for the *R* enantiomer (blue), the wide gap is from 60 to 190°, i.e., 130° wide, while the other one, from −40 to −70°, is only 30° wide, a ratio of gap widths of 4.3:1. The entropy difference between these gaps is therefore Δ*S* = *R* ln4.3 = 12.2 J/mol/K. Each of the models at the bottom of Figure 12c depicts symbolically the mesogenic (rodlike) part of the molecule with the long (TFO-gallate) and the short (biphenyl) parts twisted to the right (D) or left (L), colored blue (*R* enantiomer), red (*S* enantiomer), or gray (nonchiral N molecule). Uniform color denotes the favored conformation, while stripes indicate the unfavored conformation restricted by the narrow energy minimum; the latter are also labeled by apostrophe.

It is likely that in the racemic mixture each enantiomer would enter that helical column in the *Fddd* lattice that better fits its preferred conformation, i.e., that with the wider angular range and higher entropy. Figure 12d,e shows the relation between molecular twist and the twist of the column into which they stack up. The twist sense in (d) enables better π-overlap than that in (e), which, we suggest, is the basis for enantiomer self-sorting in separate columns in the *Fddd* structure. In contrast, in a pure enantiomer (Figure 12f,g) only half of the molecules could occupy their favored column, while the other half would have to adopt the unfavorable low-entropy conformation and cross the high but not unsurmountable barrier 2 of 125 kJ/mol to fit in the unfavored columns. This would explain why a properly ordered *Fddd* phase does not form in pure enantiomers. Nevertheless, the fact that *Fddd*-like clusters on the scale of 40 nm and highly distorted do appear in the enantiomer (3.2 and Figures 6c,i–k and S7) is probably due to the entropically unfavored molecular conformation being balanced by the benefit of closer packing of antichiral helices. Although the interhelical interaction energy is insufficient to trigger a phase transition and establish LRO,^36^ a degree of cooperativity in the formation of the clusters does exist, as indicated by the broad *C*_*p*_ maximum in Figure 8b.

**Figure 13:**
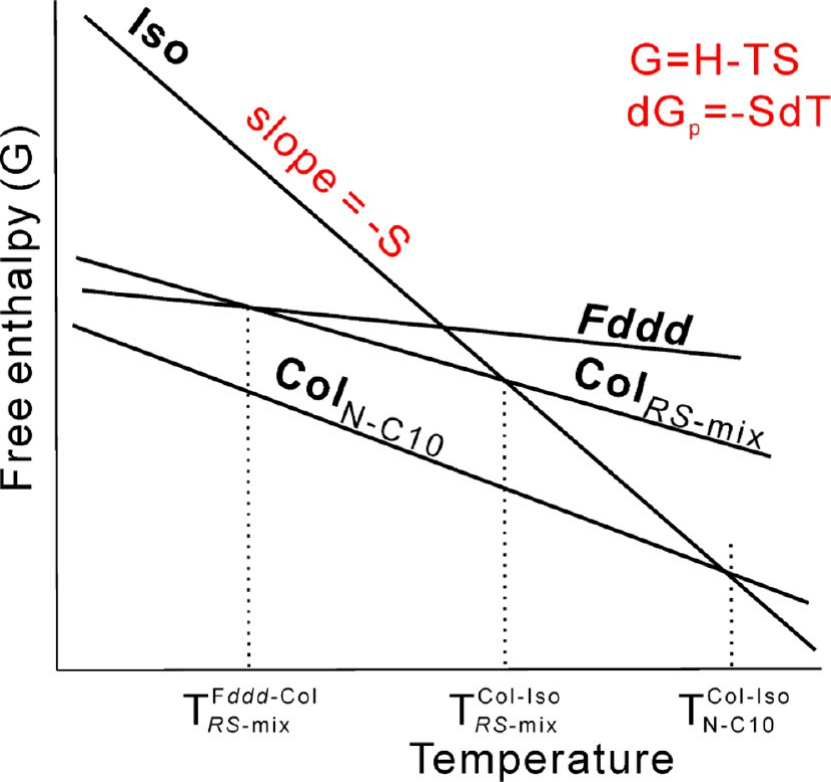
Schematic diagram of free enthalpy vs temperature for the different phases in the ***RS*****1:1-C10** mixture and in nonchiral **N–C10**.

Regarding the nonchiral **N–C10** compound, it may be surprising that it does not display the *Fddd* phase, remaining Col_h_ down to room temperature. It does not even form *Fddd*-like clusters, as indicated by the absence of a *C*_*p*_ maximum (Figure 3b) or diffuse SAXS. To explain this, we note that none of the other reported nonchiral compounds that do display the *Fddd* phase had a flexible link within their rodlike mesogen; their aromatic blocks were joined by ester groups. However, **N–C10** has a flexible ethylene (CH_2_)_2_ group breaking the rigidity of the mesogen (Figure 12b). The conformational energy diagram in Figure 12c shows only one peak arising from the clash of the carbonyl oxygen with the methylene hydrogen (peak 1_N_). That means that all of the α_3_ angles except those between +70° and −70°, are accessible at low energy and without a barrier between them. The high entropy that this provides can be realized in the Col_h_ phase but not in the *Fddd* where conformations are limited by stricter intermolecular packing. The energy benefit of *Fddd*-type packing is probably insufficient to offset the entropic gain in Col_h_ for this semiflexible compound. The extra entropy *S* puts the free enthalpy *G* = *H* – *TS* of the columnar **N–C10** phase below that of *Fddd* at all temperatures, as depicted schematically in the simple thermodynamic diagram in Figure 13. The figure compares the *G*’s of *Fddd*, columnar, and Iso (liquid) phases of the *RS* mixture with that of **N–C10**. The extra entropy of **N–C10** not only puts *G* of the columnar below that of *Fddd* but also makes the downward slope of *G*(*T*) of its Col_h_ phase steeper than that in the more rigid molecules, exemplified by the Col_*RS*-mix_ line. Consequently, it crosses the *G*(*T*) of the Iso liquid at a significantly higher temperature compared to that of its more rigid chiral analogs. As a result, while the isotropization temperatures *T*^Col-Iso^ of the enantiomers and their mixtures are all between 74 and 80 °C, that of **N–C10** is 108 °C (Figure 2c and Table 1).

We can thus predict that similarly, the *Fddd* phase may not be found in other LCs with a semiflexible mesogen.

It is also interesting to compare the situation in the *Fddd* that has an equal number of right- and left-handed twisted ribbons, with that of the Smectic-Q, a chiral bicontinuous phase consisting of two infinite networks both of the same twist sense (Figure 1f).^16^ Smectic-Q was found to only form either in chiral compounds with very high enantiomeric purity,^15^ or in completely achiral compounds,^16^ but not in chiral compounds with lower enantiomeric purity. Thus, we see again that achiral molecules are adaptable and able to join columns of either twist sense wherever they are needed, while chiral ones are restricted only to their “suitable” ribbons; if this cannot be satisfied, then the phase does not form. There are, however, cases with molecules of weaker chirality, e.g., with a chiral center in the pendant flexible chain rather than in the core, where the helix can “disregard chirality”.^63^

### 3.5 Refined Models of *Fddd* and Col_h_* Phases—Ferrochirality, Antiferrochirality, and Parachirality

**Figure 14:**
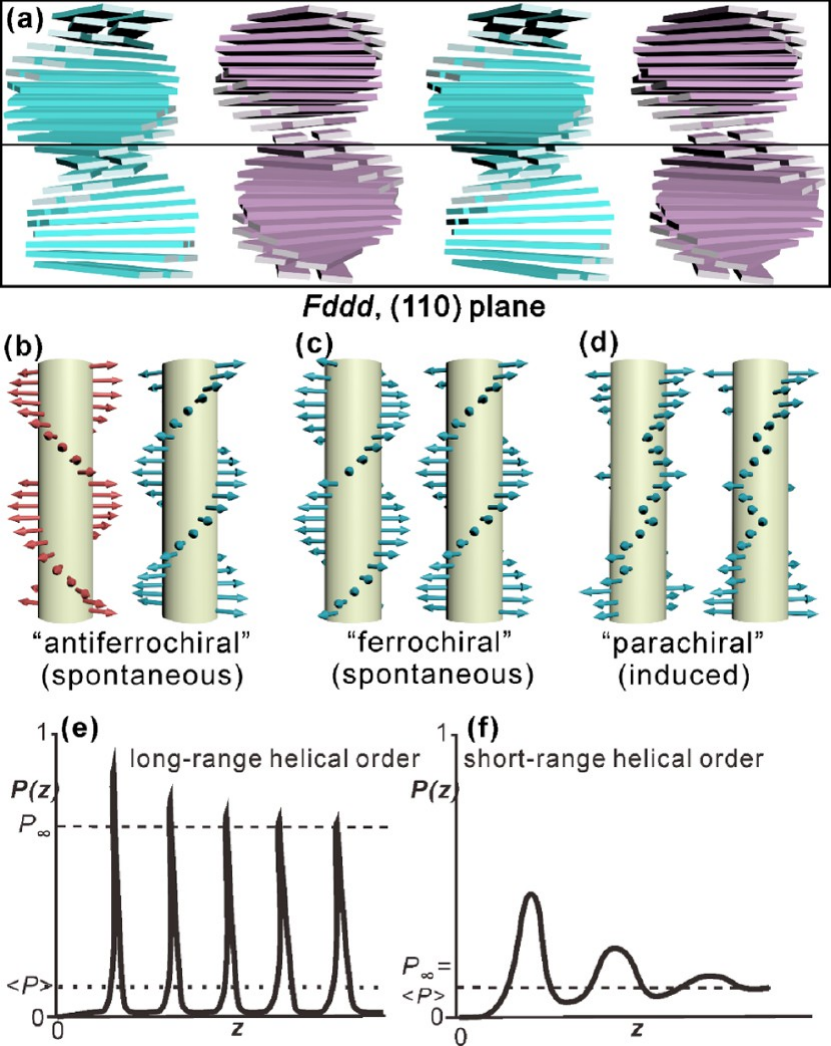
(a) Refined schematic model of two *Fddd* unit cells taking account of the tilt of the mesogen plane (side view of columns in a (110) plane) (cf. Figure 7d). (b–d) Schematic models of two columns in (b) an “antiferrochiral” phase such as *Fddd* with LRO, which exist in ***RS*****1:1-C10**, ***RS*****1:1-C12:C10**, and in compounds in refs (21,44), (c) in a hypothetical “ferrochiral” phase with LRO, and (d) a “parachiral” phase such as Col_h_* with only short-range helical order and frequent helix reversal defects, which exist in ***R*****/*****S*****-C*n*** and ***RS*****9:1-C10**. (b, c) Examples of superstructure-induced chirality, while (d) is an example of “chirality amplification” or “chirality transfer” from molecule to superstructure. (e, f) Difference between long-range (“ferro-”) order (e) and short-range (“para-”) helical order (f). Schematic plots show the probability (*P*) of a molecule being parallel to the starting molecule as a function of their distance *z* along the helix. In (e), *P* oscillates periodically with the peak values decreasing asymptotically to a value *P*_*∞*_ larger than ⟨*P*⟩, where ⟨*P*⟩ is the average *P*. In contrast, in (b), the peaks of the aperiodic oscillation decrease exponentially to ⟨*P*⟩.

The fact that the 0.35 nm streak in GIWAXS of the *Fddd* phase has a maximum away from the (horizontal) meridian (Figures 5b and S9) means that the thiophenylfluorenone plates are tilted relative to the plane normal to the column axis. As stated above the tilt is estimated as 8°. Thus, the schematic model of the phase structure in Figure 7d can be refined, as shown in Figure 14a. Further, considering that by heating above the *Fddd* temperature the negative birefringence in the Col_h_ phase becomes less negative and is also similarly less negative in columnar pure enantiomers and **N–C10** (Figure 8a), means that in the Col_h_ phase, the TFO plane is tilted at an even higher angle.

The fact that the 0.35 nm diffraction feature is a long streak rather than a spot confirms that there are no correlations in molecular position between columns and hence that the *Fddd* phase is a true LC and not a “soft crystal”. In a crude analogy, one can think of the *Fddd* phase as a close-packed array of helical tubes filled with liquid.^64^ Although the lattice of tubes is highly ordered, giving sharp Bragg diffraction at low angles, the position of the liquid molecules is not correlated between the tubes. Thus, on the scale of molecular order, i.e., < 0.5 nm, the scattering is diffuse. The existence of the 0.35 nm streak shows that correlation along the column is somewhat longer than in most liquids, but is not true LRO.

The following four experimental findings (i–iv) tell us more about what happens in the Col_h_ phase with decreasing temperature: (i) high and increasing heat capacity (Figure 8b,c); (ii) increasing birefringence (Figure 8a); (iii) increasing diffuse X-ray scattering around 2.7 nm^–1^ in anticipation of the strong Bragg reflections in the *Fddd* phase (Figure 5c,d); (iv); strong continuous increase in optical activity in the Col_h_* phase of pure enantiomers (Figure 11). All of these point to strong continuous pretransitional ordering in the Col_h_ phase, a continuous buildup of small *Fddd*-like clusters that, on a 1 min time scale, are in complete thermodynamic equilibrium (Figure 8c).

The circular birefringence and dichroism showing strong chirality of the Col_h_* phase in the enantiomer (Figure 11) are evidence of the helical nature of the columns. However, there is an important distinction between helical columns in the *Fddd* and those in the Col_h_* phase of pure enantiomers. The helical order in *Fddd* is long-range, evidenced by the strong first-order transition, resolution-limited Bragg reflections, and high order shown by atomic force microscopy (AFM) images.^20^ This order is supported by pairwise helix–helix interaction, as shown by calculations based on an intercolumnar quadrupolar charge interaction model, each dumbbell-like molecular raft being a shape-quadrupole.^20^ Helical order in the *Fddd* is spontaneous, and since nearest neighbor columns prefer to be antichiral (Figure 14a), it can be compared to antiferromagnetic or antiferroelectric. Hence, we referred to it as “antiferrochiral”,^20^ following the idea of ferrochirality by Baumgarten.^22^ In contrast, there is no long-range order in the helicity of the Col_h_* phase in our enantiomers. It is also unlikely to be there in most columnar phases of chiral compounds reported elsewhere if they were true LCs and not crystals. Noninteracting 1D chains cannot have LRO.^35,36^ The difference between short- and long-range chiral (helical) orders is illustrated schematically in Figure 14e,f. The net helicity of the columns in Col_h_* is imposed by the chiral groups, in the same way that magnetization, but not LRO, can be imposed on a paramagnet by an external field. The existence of only a broad heat capacity maximum within the Col_h_* phase of the pure enantiomers without a phase transition is consistent with the fact that new LRO cannot be established without a phase transition.^35,36^ We can thus refer to the Col_h_* phase as parachiral.

Incidentally, the analogy between molecular chirality and an external magnetic field is not new in liquid crystals. DeGennes used it in his seminal paper on the analogy between superconductivity and the nematic to smectic-A transition.^65^ Renn and Lubensky extended the analogy, stating: “The liquid-crystal analog of an external magnetic field is a field arising from molecular chirality···” The phase that they named “twist grain boundary” (TGB) was predicted to exist between the high-*T* chiral nematic and low*-T* smectic in chiral systems based on the analogy with the Abrikosov phase in type-II superconductors in an external magnetic field intervening between the high-*T* normal metal and the low-*T* Meissner phase.^66^ TGB phase was discovered experimentally 4 years later.^67^ For another example of the difference between a spontaneous and imposed order, see note (68) and ref (69).

Because of clashing end chains, there is a twist between successive molecular rafts in the columns of our compounds, but without the energetically favored interlock of *Fddd* stacking, the twist could go right or left, and there would inevitably be many helix reversals (see Figure 14d). Molecular chirality provides a bias, favoring on the one hand that will prevail on average. In fact, the quadrupolar model calculations showed that close packing of same-hand helical ribbons, as in Figure 14b, is energetically highly disfavored: while the energy of *Fddd*-type packing of antichiral helical ribbons is −45.6 energy units, that of the best packing of equal helical ribbons is +15 units.^20^ There is indeed no evidence of a columnar “ferrochiral” LC phase of homochiral ribbons.

**Figure 15:**
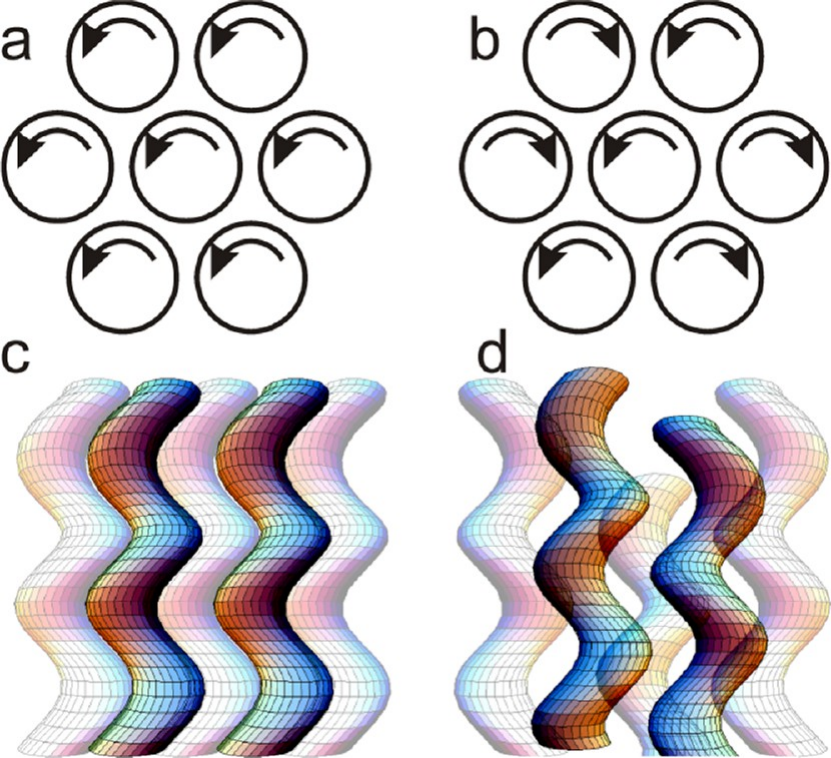
Packing of helically winding nearly cylindrical columns of LC helicene (schematic). (a, c) Ferrochiral arrangement in an enantiopure compound, (b, d) antiferrochiral arrangement in racemate, with enantiomers segregated in separate right and left helical columns. In the top view (a, b) arrows show the sense of helical rotation. In (c, d), the darker helices are in the front row, and the pale ones are behind. The helical amplitude is grossly exaggerated. Reproduced from ref (64)

Besides *Fddd* phase, we are aware of one other example of what could be regarded as “antiferrochiral” columnar LC, seen in a racemic mixture of a helicene derivative (see Figure 15b,d).^64^ There the columns were complex 6-strand helices of approximately cylindrical cross section where the column axis followed a winding helix. While having high 2D order with many *hk*0 reflections, there was only one *hk*1 Bragg reflection, indicating an LC with 3D LRO but with very high thermal disorder and a steeply decaying Debye–Waller factor. Interestingly, a similar situation was seen also in the pure helicene enantiomers, where the *hk*1 reflection was shifted along the *hk*1 layer line, suggesting an ordered packing of identical parallel helically winding columns (Figure 15a,c). This is the only case we know that could be described as a ferrochiral columnar LC. Such a structure is possible with columns with a smooth cylindrical envelope (circular cross section) but is very unlikely for ridged columns, whose cross section departs significantly from circular, as shown by a model calculation for helically twisted ribbons. In contrast, soft crystals consisting of isochiral circular columns are well covered in the literature^42,63,70^

## 4 Conclusions

Hexacatenar compounds with and without a chiral group within the rodlike mesogenic core were synthesized and found to exhibit the recently discovered *Fddd* LC phase, but only in mixtures of ***R*** and ***S*** enantiomers. As the unit cell contains 4 left- and 4 right-handed twisted ribbons, each enantiomer picks its own preferred ribbon type, leading to local deracemization. Meanwhile, pure enantiomers retain the high-*T* columnar Col_h_* phase down to room temperature. However, as shown by diffuse SAXS, high heat capacity from MDSC, and Mueller matrix circular birefringence and dichroism, locally ordered *Fddd*-like domains form increasingly in the Col_h_* phase on cooling. As suggested by conformational analysis, half of the molecules in these low-stability *Fddd* clusters of pure enantiomers are likely to adopt the intramolecularly less favored conformation for the benefit of better intermolecular packing. There is no evidence of any ordered LC phase with homochiral ribbons, confirming the high energy of such a potential structure previously calculated. Pronounced continuous ordering on cooling is also evident in the Col_h_ phase of the racemate, where it ends with a strong first-order transition to the *Fddd*. Somewhat surprisingly, the *Fddd* phase is absent not only in enantiopure compounds but also in the nonchiral **N–C10**. This absence is attributed to the flexibility of the intramesogen CH_2_CH_2_ linkage making this compound retain the entropically favored Col_h_ phase down to room temperature. Other achiral compounds, without the flexible linkage, do form the *Fddd* phase below the Col_h_.^21,44^

The twisted ribbons in *Fddd*, with a 7.54 nm pitch, consist of stacked rafts, each containing ∼2 side-by-side molecules, the successive rafts rotated by 17°. Regarding the Col_h_ phase, ultrafast flash DSC cooling scans show that its formation from the melt is extremely fast, completing in ∼20 ms. In contrast, the Col_h_-*Fddd* transition is much slower, disappearing apparently completely already at a 30 K/s cooling rate; a possible self-poisoning effect may be responsible, similar to that in the crystallization of stereocomplex of racemic polylactide.^49^ A distinction is highlighted between the spontaneous formation of ordered helices in the *Fddd* (antiferrochiral) and disordered helicity (parachiral) imposed by the chiral groups in isolated 1D columns of the Col_h_ phase in pure enantiomers. A clear effect of phase on UV–vis absorption and fluorescence emission spectra of these D–A fluorophore-containing compounds is observed.

## Supporting Information Available

Materials and methods, additional DSC, microscopy and X-ray diffraction data, structural data, additional conformational analysis data, and synthetic procedures with complete characterization data (PDF)
