# Supplementary material for: Enantiomers Self-Sort into Separate Counter-Twisted Ribbons of the Fddd Liquid Crystal—Antiferrochirality and Parachirality
Source: J Am Chem Soc. 2023 Jul 31;145(31):17443–60. doi: 10.1021/jacs.3c06164 (PMC10416214; doi:10.1021/jacs.3c06164)
Supplement: Supplementary file 1 — ja3c06164_si_001.pdf [file ja3c06164_si_001.pdf]

Supporting Infomation

Enantiomers Self-Sort into Separate Counter-Twisted Ribbons of the *Fddd* Liquid Crystal - Antiferrochirality and Parachirality

Yan Wang[1, ♣], Ya-Xin Li[2, ♣], Liliana Cseh[3], Yong-Xuan Chen[4], Shu-Gui Yang[1], Xiangbing Zeng[5], Feng Liu[1]*, Wenbing Hu[4], and Goran Ungar[1,5]*

1. State Key Laboratory for Mechanical Behaviour of Materials, Shaanxi International Research Center for Soft Matter, Xi'an Jiaotong University, Xi'an 710049, P. R. China
2. School of Chemistry and Chemical Engineering, Henan University of Technology, Zhengzhou 450001, China.
3. Romanian Academy, Coriolan Dragulescu Institute of Chemistry, Timisoara 300223, Romania
4. State Key Laboratory of Coordinate Chemistry, School of Chemistry and Chemical Engineering, Nanjing University, Nanjing 210093, China
5. Department of Materials Science and Engineering, University of Sheffield, Sheffield S1 3JD, UK.

♣ These two authors contributed equally.

Contents

1. Materials and methods 3
2. DSC thermograms 5
3. Polarized optical microscopy 6
4. Additional X-ray data 7
5. Number of molecules per column stratum 13
6. Additional results of conformational energy 14
7. Synthetic Procedures and Characterization Data 14
  7.1 Synthesis of compound A 16
  7.2 Synthesis of compound B 16
  7.3 Synthesis of compound C 16
  7.4 Synthesis of compound D 17
  7.5 Synthesis of compound E 17
  7.6 Synthesis of compound F-C10 17
  7.7 Synthesis of compound G-C10 18
  7.8 Synthesis of compound F-C12 18
  7.9 Synthesis of compound G-C12 18
  7.10 Synthesis of compound L-C10 19
  7.11 Synthesis of compound L-C12 19
  7.12 Synthesis of compound R3-C10 20
  7.13 Synthesis of compound R3-C12 20
  7.14 Synthesis of compound S3-C10 20
  7.15 Synthesis of compound N3-C10 21
  7.16 Synthesis of compound *S*-C10 21
  7.17 Synthesis of compound *R*-C10 26
  7.18 Synthesis of compound *R*-C12 30
  7.19 Synthesis of compound N-C10 32
8. References 34

1. Materials and methods

Synthesis. All chemicals and commercially available reagents were used as purchased without further purification. Dry solvents were taken from a solvent purification system. Glassware used for water-free reactions was dried for 24 h at 120 °C before use. All reactions were performed under an atmosphere of nitrogen unless stated otherwise. Columns were packed with silica gel (300–400 mesh) as the stationary phase.

Chemical analysis. ^{1}H and ^{13}C NMR and 2D NMR spectra were recorded on a Brüker DMX-600 (^{1}H: 600 MHz; ^{13}C: 150 MHz) spectrometer at 298 K. The ^{1}H and ^{13}C NMR chemical shifts are reported relative to residual solvent signals. Coupling constants (J) are denoted in Hz and chemical shifts (δ) in ppm. Multiplicities are denoted as follows: br = broad, s = singlet, d = doublet, m = multiplet, t = triplet. High-resolution mass spectral (HRMS) data were obtained on an electrospray (ESI) mass time-of-flight spectrometer (Waters i-Class VION IMS QToF). Elemental analysis was done on Elementar Vario EL cube/Vario OXY cube element analyzer.

Conventional differential scanning calorimetry (DSC) thermograms were recorded on a TA DSC250 calorimeter, purged with nitrogen. Heating and cooling rate was 3 K/min unless stated otherwise. Temperature, thermal resistance of the cell and enthalpy calibration were performed using Indium and distilled water. Peak transition temperatures are quoted, corrected for thermal lag. Temperature calibration for cooling runs was performed by linear extrapolation of temperature error to negative heating rates.

Modulated DSC (MDSC) experiments were performed on the same instrument. Linear heating/cooling rate was 0.04 K min^{-1}, modulation amplitude 0.07 K and modulation period 20-60 s. Heat capacity was calibrated with a sapphire standard.

Flash DSC 1 (Mettler-Toledo) instrument was used for very high heating and cooling rates. The instrument was equipped with a UFS1 chip sensor as well as a TC 100 intracooler (Huber) that set the sample-support temperature at -90 ^{o}C. The cell compartment was purged with Nitrogen at a rate of 50 mL min^{-1}. UFS1 sensor was conditioned according to the standard protocol. The temperature was calibrated by the melting temperature of a standard Indium sample. Samples were prepared by using a scalpel to cut thin slices with smooth top and bottom surfaces under optical microscope. The lateral sizes of the thin sections were reduced to 50-100 μm. The sample section was then transferred to the center area of the chip sensor by utilizing a string of hair.

For **polarized optical microscopy** (POM) an Olympus BX51 microscope was used equipped with a Linkam LTS420E hot stage and a T95-HS controller. The samples were heated to the isotropic melt between glass slides and cooled to desired temperature at a rate of 1 K/min. **Birefringence** was measured with a Berek compensator. Sample thickness was determined by measuring the total area of a known sample weight.

UV-vis absorption spectra were measured on a Lambda 950 absorption spectrophotometer. Solid samples were melted between quartz glass windows. The sample were heated to isotropic melt and held to stabilize, then cooling at a rate of 2 K/min. A Specac heated cell and controller were used.

Fluorescence emission spectra were recorded on an Edinburgh FLS9 spectrophotometer. Solid samples were held between quartz glass windows and temperature controlled as above.

Chiro-optical measurements were carried out at Beamline B23 of Diamond Light Source using the Mueller matrix method[S1] which is capable of separating the effects of linear birefringence and dichroism from those of CD and optical activity. The method uses four photoelastic modulators, with the ability to measure simultaneously all the Mueller elements while avoiding errors associated with mechanical or optical rotation.[S2] The synchrotron-generated light beam was reflected vertically which allowed it to pass through the sample film between quartz glass windows to lie horizontally to avoid flow of the liquid. The sample sandwich was held in a Linkam hot-stage.

Synchrotron X-ray diffraction. **Powder** small/wide-angle X-ray scattering (SAXS/WAXS) experiments were conducted at beamline BL16B1 of Shanghai Synchrotron Radiation Facility, China, and beamline I22 of Diamond Light Source, U.K. Two Pilatus 2M detectors (Dectris) were used simultaneously at Diamond, one for SAXS and another for WAXS. X-ray energy was 12.4 keV. At both facilities the samples in 1mm glass capillaries were held in a modified Linkam hot stage. During the experiment the capillary was rotated by an electromotor at ca 200 rpm to improve signal averaging. **Grazing incidence** small/wide angle X-ray scattering experiments (GISAXS/GIWAXS) were conducted at beamline BM28 of European Synchrotron Radiation Facility, France, and I16 of Diamond Light Source, U.K. 2D diffraction patterns were collected using a MAR165 CCD camera at B28 and a Pilatus 2M detector at I16. A home-made hot stage controlled by a Eurotherm was used. Samples were spin coated onto silicon substrates. n-tetracontane was used to calibrate the sample to detector distance (SDD) as well as the temperature, using the known melting point of the standard. Separate **off-line SAXS/WAXS** experiments were carried out on a SAXSpoint 2.0 instrument from Anton Paar, equipped with a 50 kV/1 mA sealed-tube microfocus generator, multilayer focusing mirrors and an Eiger R 1M decector (Dectris). Powder samples were held in 1mm glass capillaries in an Anton Paar Heated Sampler 2.0, all in vacuum. Sample temperature was calibrated by several standard substances, and SDD by silver behenate. Fibrefix software was used for general data processing.

Electron density maps were calculated by inverse Fourier transformation using standard procedure as described in International Tables for Crystallography. Integral intensities of powder SAXS peaks were measured using Gaussian peak fitting. GISAXS intensities were used to help resolve overlapping peaks.

Calculation of **conformational potential energy** was performed using the ForcitePlus module in Materials Studio (Accelrys). Molecular segments of interest were geometry optimized before each 10° rotational increment around bonds 1, 2 and 3, marked in Figures 11a,b and S15. The molecule’s energy was calculated after each rotation. The energy is the sum of valence energy (e.g. bond, torsion, stretch-stretch, torsion-stretch) and non-bonded interaction energy (van der Waals and electrostatic).

2. DSC thermograms

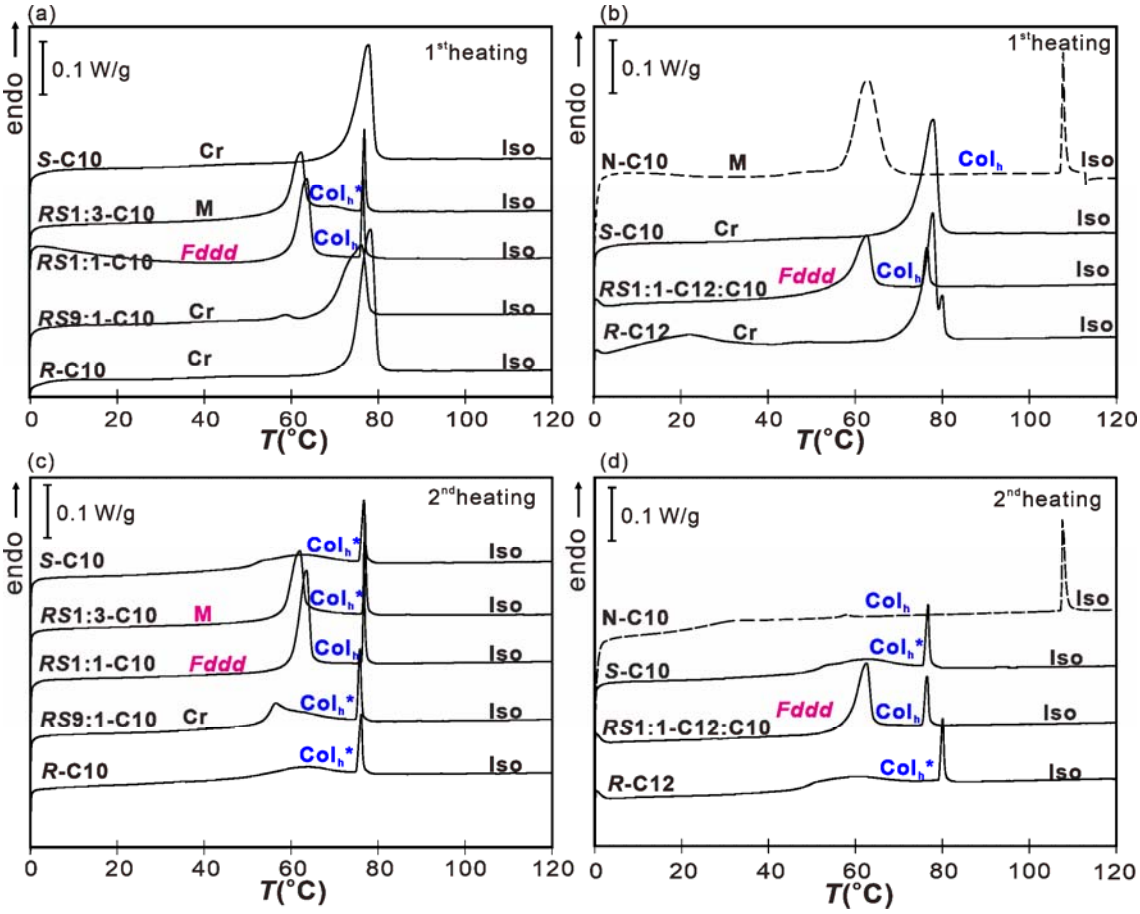

Figure S1. First (a,b) and second (c,d) DSC heating traces of compounds and mixtures: (a,c) ***R*-C10**, ***RS*9:1-C10**, ***RS*1:1-C10**, ***RS*1:3-C10** and ***S*-C10**, and (b,d) ***R*-C12**, ***RS*1:1-C12:C10**, ***S*-C10**, and **N-C10**. All scan rates 3 K min^{-1}.

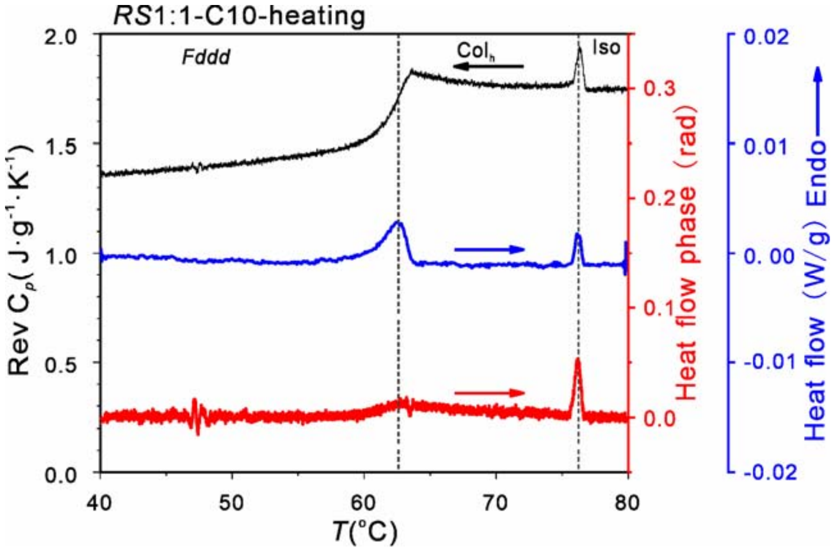

Figure S2. MDSC of ***RS*1:1-C10** recorded in heating. Compare with Figure 7c. Linear heating rate 0.04 K/min, amplitude 0.07 K, period 20 s.

3. Polarized optical microscopy

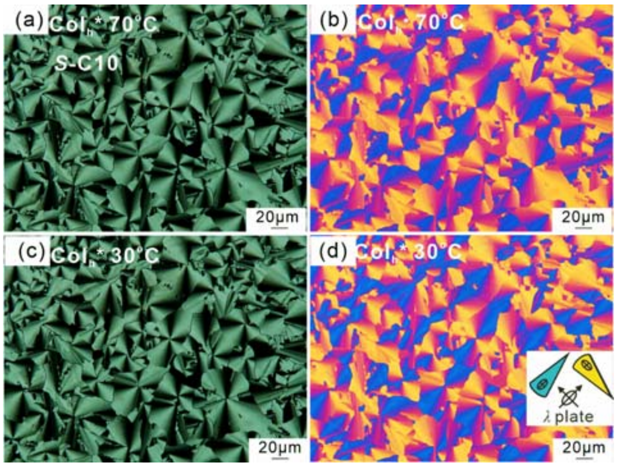

Figure S3. Texture of compound ***S*-C10** observed between crossed polarizers after cooling from isotropic state at 5 K/min to (a,b) 70 °C and (c,d) 30 °C. (a,c) without and (b,d) with lambda plate.

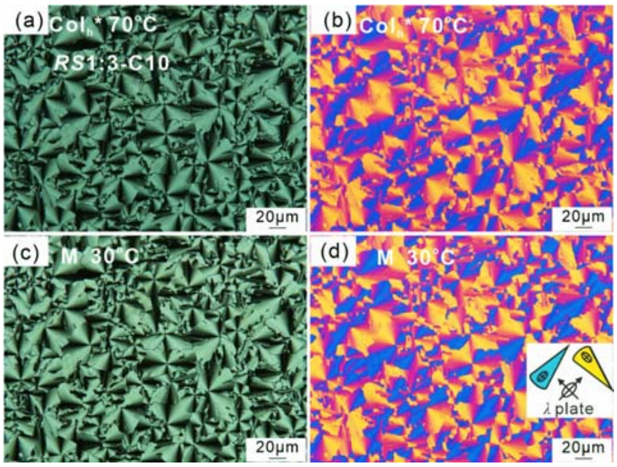

Figure S4. Same as Figure S3 but for ***RS*-1:3-C10**. Cooling rate 1 K/min.

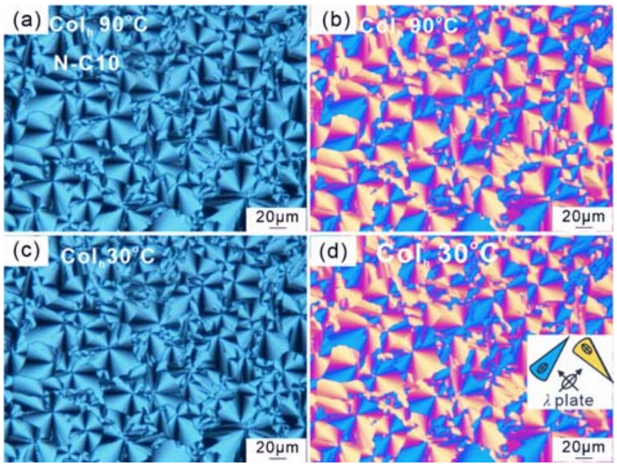

Figure S5. Same as Figure S4 but for compound **N-C10**. (a,b) 90 °C, (c,d) 30 °C.

4. Additional X-ray data

S-C10

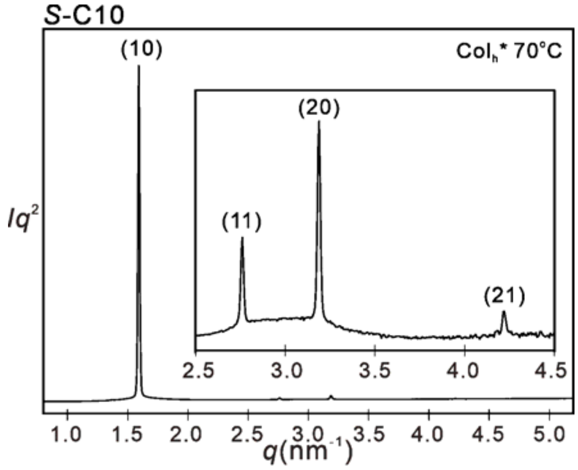

Figure S6. SAXS diffractogram of ***S*-C10** at 70°C. Col$_h$* phase. Intensity scale is expanded in the inset.

Table S1: Indices, experimental and calculated *d*-spacings, intensities, lattice parameters and structure factor phases of the columnar phase with ***p6mm*** symmetry in ***S*-C10** recorded at 70 °C. In this and subsequent tables all intensities are Lorentz and multiplicity corrected.

(*hk*)	$d_{obs.}$ (nm)	$d_{cal.}$ (nm)	intensity	phase
(10)	3.95	3.95	100	0
(11)	2.28	2.28	0.4	π
(20)	1.97	1.97	1.2	π
(21)	1.49	1.49	0.1	π
a_{col^*} = 4.56 nm				

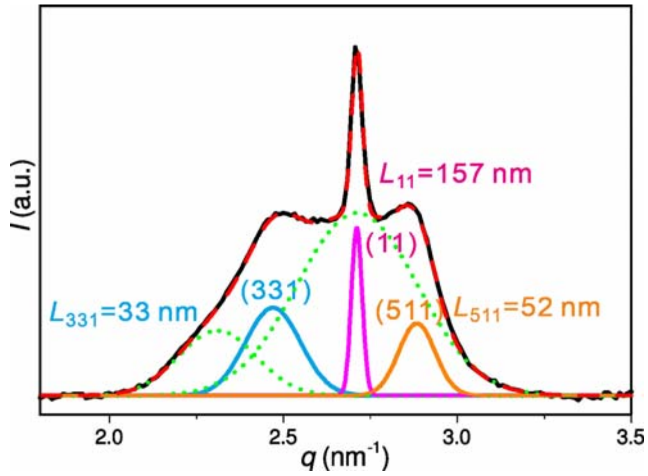

Figure S7. Radial scan of GISAXS of ***S*-C10** around the small *Fddd*-like clusters in Figure 5e (dashed line). The fitted peaks are from both *Fddd* clusters ((331) and (511)) and Col$_h$ phases (11), shown by full lines. The dotted green lines are components representing the diffuse background. Coherence lengths *L* are indicated.

***RS*1:1-C10**

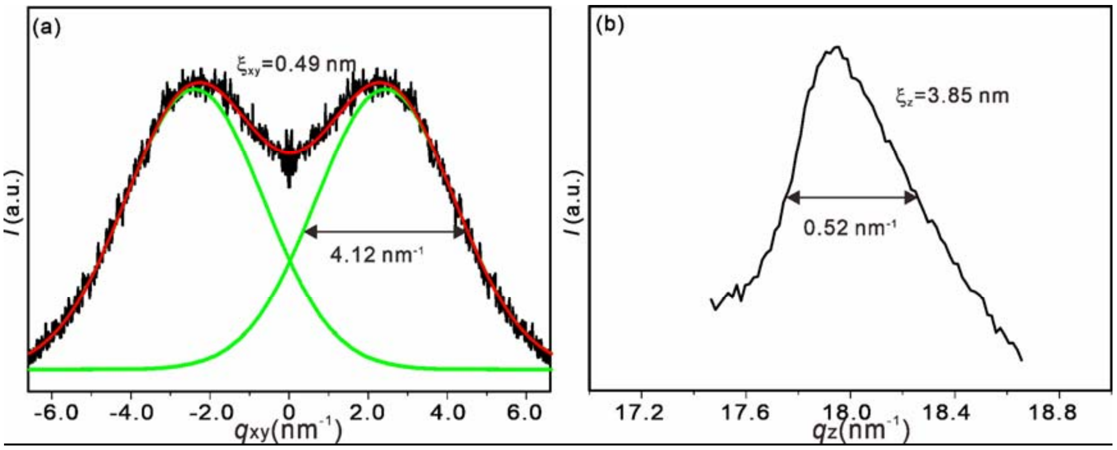

Figure S8. q_{xy} (vertical) and q_z (horizontal) scans of the 3.5 nm streak in GIWAXS of *Fddd* phase of ***RS*1:1-C10** in Figure 5b; in (a) the peak is reflected across the horizon to facilitate curve resolution.

Table S2: Indices, experimental, calculated *d*-spacings, intensities, phases and lattice parameters of diffraction peaks of ***RS*1:1-C10** recorded by powder SAXS in the ***Fddd*** phase at 40 °C.

(*hkl*)	$d_{obs.}$- spacings (nm)	$d_{cal.}$- spacings (nm)	intensity	phase
(400)<br>(220)	4.01	4.01	100.0<br>93.0	π<br>π
(511)<br>(131)	2.36	2.36	2.6<br>0.3	π<br>π
(620)	2.32	2.32	4.3	0
(440)<br>(800)	2.01	2.01	0.4<br>0.8	/
(731)<br>(151)	1.65	1.65	0.2<br>0.2	/
(911)<br>(351)	1.59	1.59	0.5<br>0.5	/
(840)<br>(260)<br>(10 2 0)	1.52	1.52	1.2<br>1.2<br>1.2	/
a = 16.04 nm, *b* = 9.26 nm, *c* = 3.77 nm				

***R*-C12**

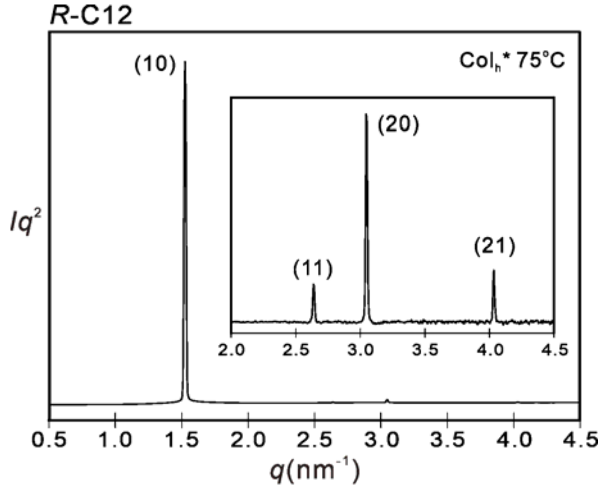

Figure S9. SAXS diffractogram of ***R*-C12** in the Col$_h$* phase at 75°C. Intensity scale is expanded in the inset.

Table S3: Indices, experimental and calculated *d*-spacings, relative integrated intensities and unit cell parameters of the columnar phase with ***p*6*mm*** symmetry of ***R*-C12** recorded at 75 °C.

(*hk*)	$d_{obs.}$- spacings (nm)	$d_{calc.}$- spacings (nm)	intensity	phase
(10)	4.13	4.13	100	0
(11)	2.38	2.38	0.2	π
(20)	2.06	2.06	0.8	π
(21)	1.56	1.56	0.1	π
a_{col*} = 4.76nm				

***RS*1:1- C12:C10**

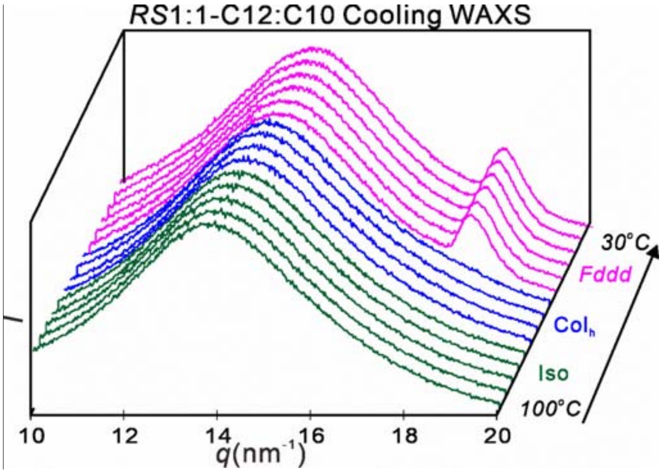

Figure S10. WAXS diffractograms of ***RS*1:1-C12:C10** recorded during cooling from 100 to 30 °C using SAXSPoint 2.0 instrument.

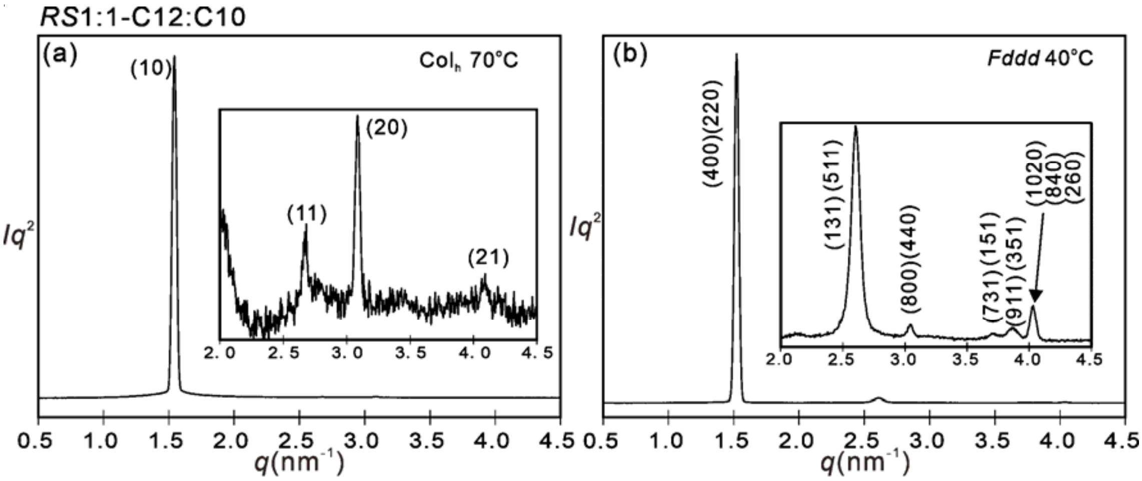

Figure S11. SAXS diffractograms of ***RS*1:1-C12:C10** recorded at indicated temperatures on cooling.

Table S4: Indices, experimental and calculated *d*-spacings, intensities and parameters of the columnar phase with ***p6mm*** symmetry of ***RS*1:1- C12:C10** at 70 °C.

(*hk*)	$d_{obs.}$- spacings (nm)	$d_{calc.}$- spacings (nm)	intensity	phase
(10)	4.07	4.07	100	0
(11)	2.35	2.35	0.1	π
(20)	2.04	2.04	0.3	π
(21)	1.54	1.54	0.1	π
a_{col} = 4.70nm				

Table S5. Indices, experimental, calculated *d*-spacings, intensities, phases and lattice parameters of diffraction peaks of ***RS*1:1-C12:C10** recorded by powder SAXS in the ***Fddd*** phase at 40 °C.

(*hkl*)	$d_{obs.}$- spacings (nm)	$d_{calc.}$- spacings (nm)	intensity	phase
(400)<br>(220)	4.13	4.13	100.0<br>93.0	/
(511)<br>(131)	2.41	2.41	1.7<br>0.3	/
(440)<br>(800)	2.07	2.07	0.1<br>0.1	/
(731)<br>(151)	1.69	1.69	0.1<br>0.1	/
(911)<br>(351)	1.62	1.63	0.1<br>0.1	/
(840)<br>(260)<br>(10 2 0)	1.56	1.56	0.1<br>0.1<br>0.1	/
a = 16.52, *b* = 9.54m, *c* = 3.78m				

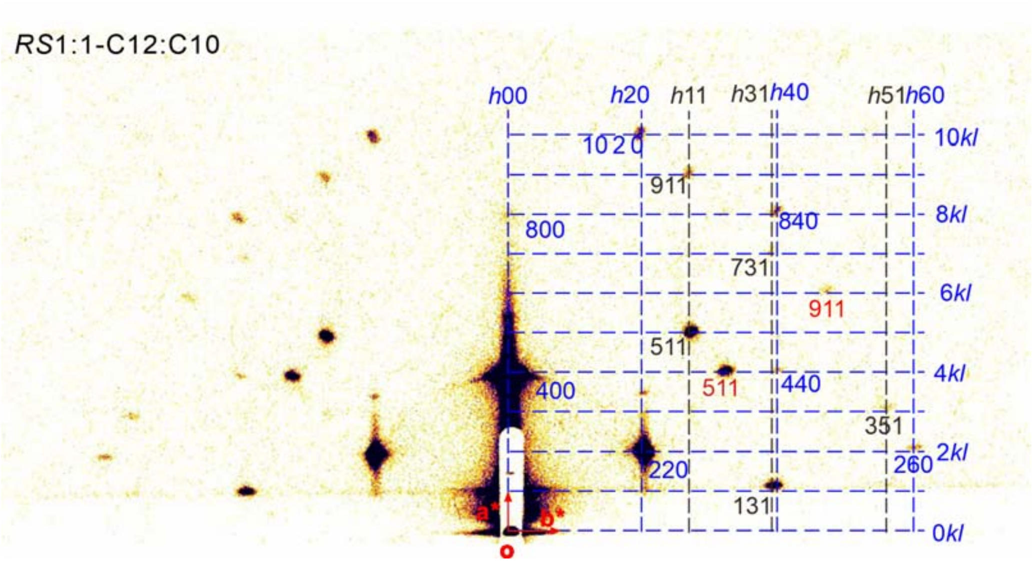

Figure S12. GISAXS diffractograms of ***RS*1:1- C12:C10** recorded at 40°C in the cooling scan.

N-C10

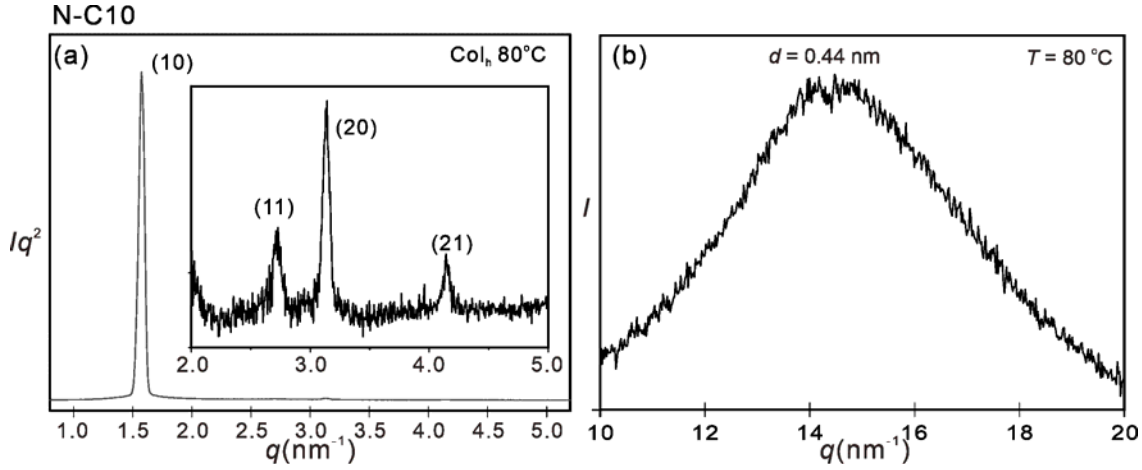

Figure S13. (a) SAXS and (b) WAXS diffractograms of **N-C10** in the Col_h phase at 80°C on cooling.

Table **S6**: Indices, experimental and calculated *d*-spacings, intensities and parameters of the columnar phase with ***p*6*mm*** symmetry of **N-C10** at 80 °C.

(*hk*)	$d_{obs.}$- spacings (nm)	$d_{calc.}$- spacings (nm)	intensity	phase
(10)	3.98	3.98	100	0
(11)	2.30	2.30	0.13	π
(20)	2.00	2.00	0.43	π
(21)	1.51	1.51	0.05	π
a_{col} = 4.60nm				

5. Number of molecules per column stratum

Table S7 Calculation of number of molecules per column stratum (a stratum is a section of the column one molecule thick).

Compound	Phase	Lattice parameters (nm)	V_{mol} (nm^3)[a]	Estimated or observed cell height (nm)[b]	$V_{stratum}$ (nm^3)[c]	μ^d
***S*-C10**	Col_h*	a_{Col*} = 4.56	2.80	0.46	8.28	3.0
	Fddd	a_{Orth}= 16.24<br>b_{Orth} = 9.37<br>c_{Orth} = 3.15		0.35	6.65	2.4
***R*-C10**	Col_h*	a_{Col*} = 4.55		0.47	8.42	3.0
***R*-C12**	Col_h*	a_{Col*} = 4.76	3.09	0.47	9.22	3.0
**N-C10**	Col_h	a_{Col} = 4.60	2.93	0.48	8.25	3.0
***RS*1:1-C10**	Col_h	a_{Col} = 4.54	2.80	0.47	8.39	3.0
	Fddd	a_{Orth}= 16.04<br>b_{Orth} = 9.26<br>c_{Orth} = 3.77		0.35	6.50	2.3
***RS*9:1-C10**	Col_h*	a_{Col*} = 4.56	2.80	0.46	8.30	3.0
***RS*1:1-C12:C10**	Col_h	a_{Col} = 4.70	2.94	0.45	8.61	3.0
	Fddd	a_{Orth}= 16.52<br>b_{Orth} = 9.54<br>c_{Orth} = 3.78		0.35	6.89	2.3

a: The volume of molecule is calculated by the density method, $V_{mol} = M/\rho N_A$, where ρ is density, M is molecular mass and N_A is Avogadro's number. Based on data on similar materials, ρ = 0.95 g cm^{-3} is assumed. For the mixtures, V_{mol} is the averaged volume of the two compounds.

b: The estimated height of Col phase is the average intermolecular distance along the colum axis, within 0.46±0.01nm. The observed height of *Fddd* phase is determined from the position of the meridional streak in GIWAXS.

c: Volume of a column stratum. For *Fddd* phase: $V_{strat} = abh/8$; For Col phase: $V_{strat} = a^2h \sin 60°$

d: Number of molecules per column stratum μ is calculated from $\mu = V_{strat}/V_{mol}$.

6. Additional results of conformational energy

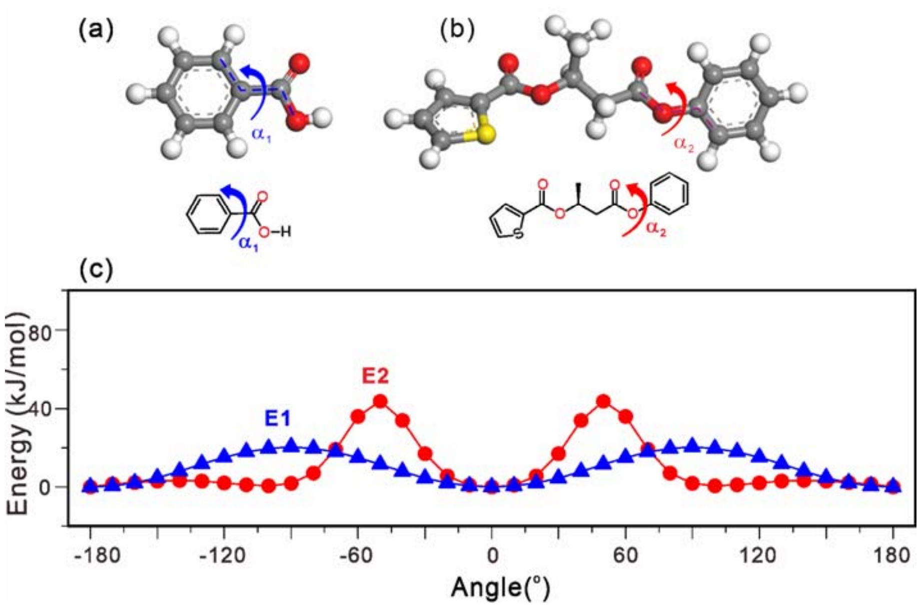

Figure S14. (a, b) Geometry-optimized structure of a molecular segment of ***S*-C10** containing (a) bond 1 and (b) bonds 2 and 3. (c) Torsion angles α_1 and α_2 were varied in steps of 10° and potential energies $E1(\alpha_1)$ (green) and $E2(\alpha_2)$ (orange) of the segments were calculated and plotted in the diagram.

7. Synthetic Procedures and Characterization Data

Abbreviations:

MeOH **-** methanol

THF- tetrahydrofuran

DCM - dichloromethane

AcOH - acetic acid

PE - petroleum eter

EA - ethyl acetate

DMAP - 4-dimethylaminopyridine

EDC·HCl - 1-ethyl-3-(3-dimethylaminopropyl)carbodiimide hydrochloride

MTBE – methy tert-butyl ether

r.t . - room temperature

h – hour

aq - aqueous

SGCC- silica gel column chromatography

3,4,5-tris(dodecyloxy)benzoic acid [S3] was prepared according to the procedure in the literature.[S4,]

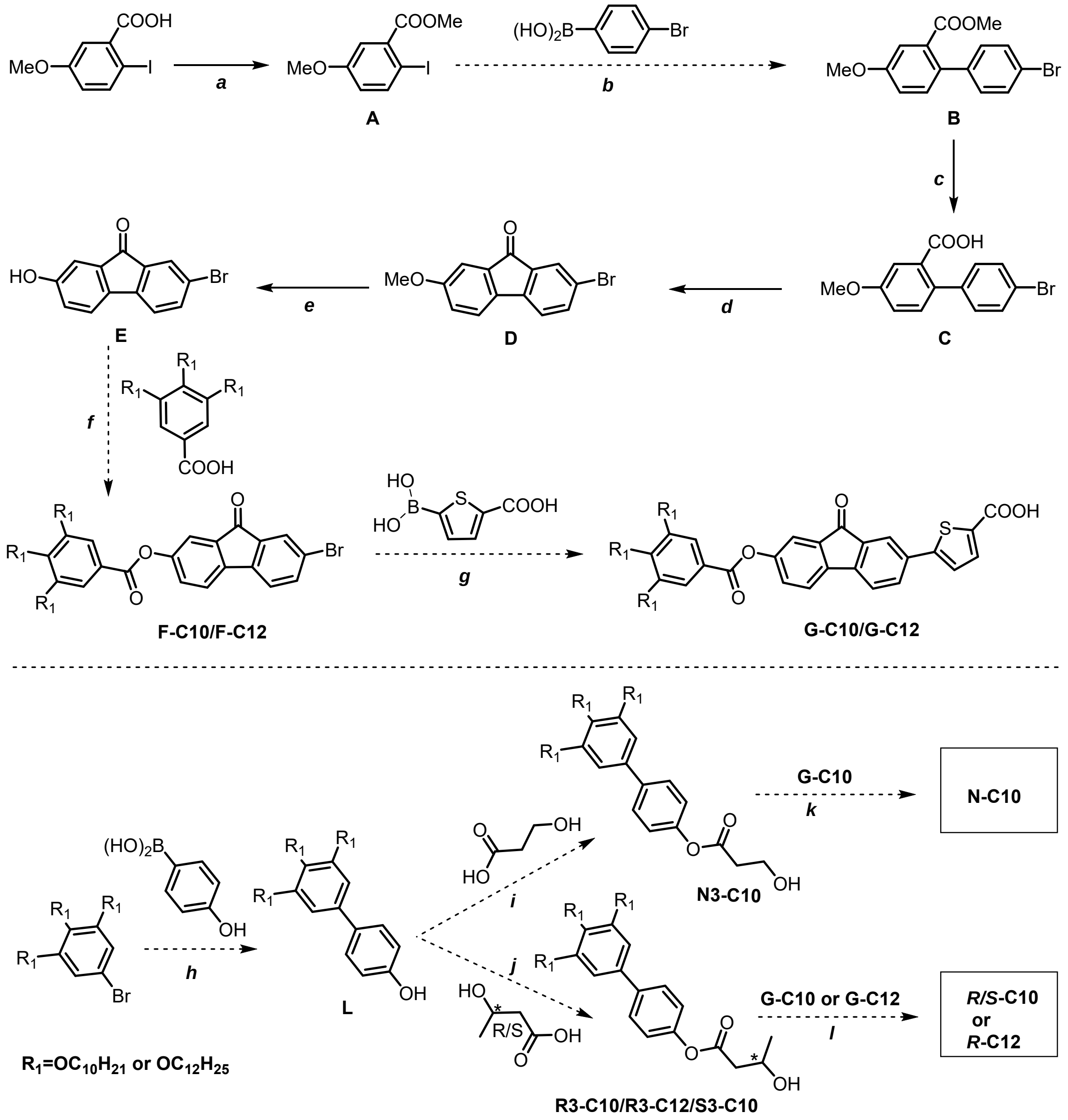

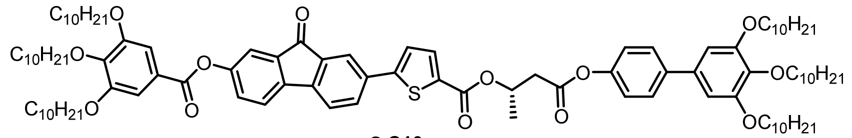

***S*-C10**

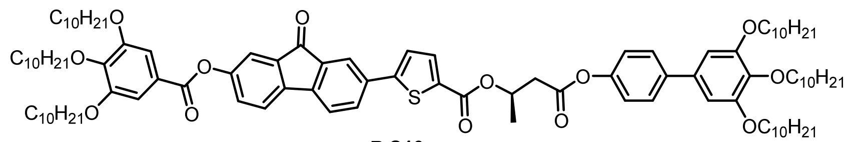

***R*-C10**

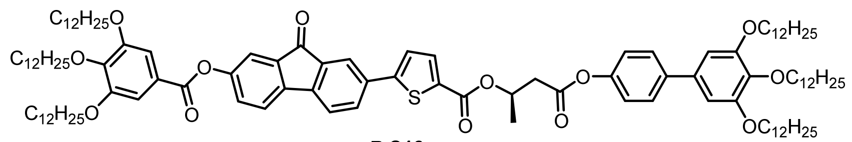

***R*-C12**

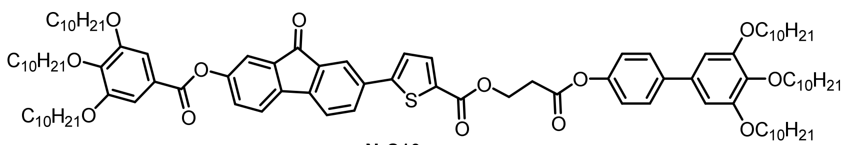

N-C10

Scheme S1. Synthesis of ***R/S*-Cn and N-Cn**. Reagents and conditions: (a) H_2SO_4, MeOH, 80 °C; (b) $Pd(PPh_3)_2Cl_2$, THF, 2M Na_2CO_3. 80 °C; (c) THF, MeOH, KOH, 80 °C; (d) $SOCl_2$, $AlCl_3$, DCM, r.t.; (e) HBr, AcOH, 120 °C; (f)(i)(j) EDC·HCl, DMAP, DCM, r.t.; (g) $Pd(PPh_3)_2Cl_2$,THF, 2M Na_2CO_3, 80 °C; (h) $Pd(PPh_3)_4$, THF, K_2CO_3, H_2O, 80 °C; (k)(l) $SOCl_2$, pyridine, DCM, r.t.

7.1 Synthesis of compound A

To a solution of *2*-iodo-*5*-methoxybenzoic acid **1** (11.12 g, 40 mmol) in MeOH (100 ml) was added conc. H_2SO_4 (5 ml) at 0 °C. The mixture was allowed to warm up to r.t., followed by reflux for 3 h. The solvent was removed in vacuum. The solid was dissolved in DCM (100 ml) and the organic phase was washed with saturated $NaHCO_3$ (2×20 ml), water (1×20 ml) and brine (20 ml), respectively. The organic phase was dried over Na_2SO_4, filtered and concentrated under vacuum. The crude products were purified by SGCC (PE/EA = 200:1) and the compound **A** was obtained as a colorless oil liquid (11.45 g, 98%). **^{1}H NMR** (600 MHz, $CDCl_3$) δ 8.03 – 7.61 (m, 1H), 7.46 – 7.09 (m, 1H), 6.86 – 6.62 (m, 1H), 3.95 – 3.88 (m, 3H), 3.81 – 3.75 (m, 3H). **^{13}C NMR** (151 MHz, $CDCl_3$) δ 166.62 (s), 159.41 (s), 141.79 (s), 135.71 (s), 119.29 (s), 116.39 (s), 82.40 (s), 55.52 (s), 52.49 (s). **ESI-HR-MS**: *m/z* for $C_9H_{10}IO_3^+$ [**A**+H$^+$] calc.: 292.9669, found: 292.9674.

7.2 Synthesis of compound B

$Pd(PPh_3)_2Cl_2$ (175 mg, 0.25 mol) catalyst was added to a mixture of 4-bromophenylboronic acid (1.51 g, 7.5 mmol), methyl 2-iodo-5-methoxybenzoate (1.46 g, 5 mmol), 2M Na_2CO_3 solution (6.2 ml) and THF (25 ml), under nitrogen. The mixture was heated at 80 °C for 24 h. After cooling to r.t., the reaction mixture was poured into water and extracted with EA (3X25 ml). The combined organic layers were dried over anhydrous Na_2SO_4, filtered and the solvent was evaporated. The crude product was purified by SGCC (PE/EA = 50:1) to afford the compound **B** as a light yellow oil liquid (1.28 g, 80%). **^{1}H NMR** (600 MHz, $CDCl_3$) δ 7.49 (d, *J* = 8.2 Hz, 2H), 7.37 – 7.35 (m, 1H), 7.24 (d, *J* = 8.5 Hz, 1H), 7.14 (d, *J* = 8.3 Hz, 2H), 7.06 (dd, *J* = 8.5, 2.7 Hz, 1H), 3.87 (s, 3H), 3.66 (s, 3H). **^{13}C NMR** (151 MHz, $CDCl_3$) δ 168.53 (s), 158.84 (s), 140.06 (s), 133.80 (s), 131.81 (s), 131.37 (s), 131.09 (s), 130.13 (s), 121.12 (s), 117.65 (s), 114.72 (s), 55.57 (s), 52.11 (s). **ESI-HR-MS**: *m/z* for $C_{15}H_{13}BrO_3Na^+$, [**B**+Na$^+$] calc.: 342.9940, found: 342.9937.

7.3 Synthesis of compound C

A mixture of compound **B** (6.423 g, 20 mmol), THF (40 ml), MeOH (200 ml) and NaOH solution (4 g in 15 ml H_2O) as refluxed overnight. The mixture was treated with 4M HCl until pH =2-3. The white precipitate was isolated and washed with MeOH and water. The product was recrystallized from anhydrous ethyl alcohol. The compound **C** was obtained as white powder (5.87 g, 96%).**^{1}H NMR** (600 MHz, $CDCl_3$) δ 7.49 (dd, *J* = 5.6, 2.3 Hz, 3H), 7.23 (d, *J* = 8.4 Hz, 1H), 7.17 (d, *J* = 8.1 Hz, 2H), 7.11 (dd, *J* = 8.4, 2.4 Hz, 1H), 3.88 (s,3H). **^{13}C NMR** (151 MHz, $CDCl_3$) δ 172.37 (s), 158.85 (s), 139.84 (s), 134.82 (s), 132.33 (s), 131.14 (s), 130.35 (s), 129.69 (s), 121.32 (s), 118.71 (s), 115.41 (s), 55.64 (s). **ESI-HR-MS**: *m/z* for $C_{14}H_{11}BrO_3K^+$, [C+K$^+$], calc.: 344.9523, found: 344.9524.

7.4 Synthesis of compound D

The compound **C** (620 mg, 2.02 mmol) and $SOCl_2$ (15 ml) was stirred overnight at r.t. The excess of $SOCl_2$ was removed under vacuum and the solid acid chloride was dissolved in anhydrous DCM (10 ml) under nitrogen atmosphere. This solution was cooling at 0ºC and aluminium(III) chloride (0.490 g, 6.06 mmol) was added. After stirring 12 h at r.t. the reaction was quenched with a mixture of ice and cold water (200 ml water + 50 g ice). The compound was extracted with DCM (3×100 ml), The organic phases were wash with water (2x100 ml). The collected organic layers were dried over anhydrous $MgSO_4$, filtered and concentrated. It was obtained the product **D** as orange solid (554.85 mg, 96%). **^{1}H NMR** (600 MHz, CDCl3) δ 7.70 (d, J = 1.7 Hz, 1H), 7.55 (ddd, J = 7.9, 5.6, 1.9 Hz, 1H), 7.38 (dd, J = 8.2, 1.7 Hz, 1H), 7.27 (t, J = 2.5 Hz, 1H), 7.19 (t, J = 2.0 Hz, 1H), 7.03 – 6.95 (m, 1H), 3.86 (dd, J = 8.5, 1.8 Hz, 3H).**^{13}C NMR** (151 MHz, CDCl3) δ 192.40 (s), 161.09 (s), 143.50 (s), 137.21 (s), 136.24 (s), 135.96 (s), 135.51 (s), 127.55 (s), 121.58 (s), 121.54 (s), 120.99 (s), 120.64 (s), 109.62 (s), 55.78 (s). **ESI-HR-MS:** m/z for $C_{14}H_{10}BrO_2^+$, ([M]+H$^+$), calc.: 288.9859, found: 288.9860.

7.5 Synthesis of compound E

A 25-ml round-bottom flask equipped with a stir bar was charged with **D** (607 mg, 2.1 mmol) followed by the addition of AcOH (2.65 ml) and 48% HBr (8.82 ml). The mixture was heated at 120 ºC for 120 h. After cooling to r.t., the reaction mixture was poured into water (30 ml) and extracted with MTBE (3×15 ml). The combined organic layers were dried over anhydrous $MgSO_4$, filtered and evaporated under vacuum to afford the product **E** as a dark-red solid (554.6 mg, 96%). **^{1}H NMR** (600 MHz, DMSO) δ 8.74 (s, 1H), 7.73 (dd, *J* = 7.9, 1.9 Hz, 1H), 7.62 (d, *J* = 1.9 Hz, 1H), 7.61 – 7.55 (m, 2H), 6.97 (s, 2H).**^{13}C NMR** (151 MHz, DMSO) δ 192.29 (s), 159.65 (s), 144.18 (s), 137.95 (s), 135.75 (s), 135.31 (s), 134.43 (s), 126.90 (s), 123.27 (s), 122.41 (s), 121.82 (s), 120.87 (s), 111.64 (s). **ESI-HR-MS:** m/z for $C_{13}H_8BrO_2$+, ([M]+H$^+$), calc.: 274.9702, found: 274.9699.

7.6 Synthesis of compound F-C10

A mixture of 3,4,5-tris(decyloxy)benzoic acid (8 g, 13.55 mmol), **E** compound (3.7 g, 13.55 mmol), EDC·HCl (3.9 g, 20.3 mmol) and DMAP (622 mg, 5.42 mmol) in DCM (68 ml) was stirred for 16 h at r.t. Extra solvent – DCM (100 ml) was added and the mixture was washed with water (3 ×50 ml). The collected organic phase was dried over $MgSO_4$ and filtered. The solvent was evaporated under vacuum to give the crude compound, which was purified by SGCC (PE/EA = 50:1) to afford the compound **F** as a yellow solid (9.18 g, 80%). **^{1}H NMR** (600 MHz, $CDCl_3$), δ 7.72 (s, 1H), 7.58 (d, *J* = 7.8 Hz, 1H), 7.52 – 7.46 (m, 2H), 7.39 (s, 2H), 7.36 – 7.29 (m, 2H), 4.06 (dt, *J* = 17.4, 6.4 Hz, 6H), 1.88 – 1.81 (m, 4H), 1.80 – 1.72 (m, 2H), 1.49 (dt, *J* = 14.8, 7.3 Hz, 6H), 1.39 – 1.21 (m, 36H), 0.88 (q, *J* = 6.6 Hz, 9H). **^{13}C NMR** (151 MHz, $CDCl_3$) δ 190.14 (s), 163.66 (s), 151.99 (s), 151.11 (s), 142.27 (s), 141.51 (s), 139.92 (s), 136.32 (s), 134.98 (s), 134.15 (s), 126.98 (s), 126.70 (s), 122.18 (s), 121.83 (s), 120.68 (s), 120.25 (s), 117.59 (s), 107.57 (s), 72.59 (s), 68.25 (s), 30.93 (s), 30.90 (s), 30.48 (s), 29.34 (s), 29.11 (s), 28.72 (s), 28.66 (s), 28.62 (s), 28.57 (s), 28.55 (s), 28.38 (s), 28.34 (s), 28.28 (s), 25.07 (s), 25.04 (s), 21.69 (s), 21.67 (s), 13.10 (s). **ESI-HR-MS**: *m/z* for $C_{50}H_{72}BrO_6^+$, ([M]+H$^+$), calc.: 847.4507, found: 847.4485.

7.7 Synthesis of compound G-C10

A mixture of 7-bromo-9-oxo-9H-fluoren-2-yl 3,4,5-tris(decyloxy)benzoate (848 mg, 1 mmol), 2-carboxythiophene-5-boronic acid (260 mg, 1 mmol), $Pd(PPh_3)_2Cl_2$ (175 mg), 2 M Na_2CO_3 solution (2.0 ml) and THF (10 ml) was heated at 80 °C for 36 h under nitrogen. After cooling to r.t., the reaction mixture was poured into water and then acidified with 2M HCl to pH = 3, wherein the brown precipitate appeared. The precipitate was isolated and dissolved in chloroform. The organic phase was dried over anhydrous Na_2SO_4, filtered and the solved was evaporated. The crude product was purified by SGCC (PE/EA = 50:1) to afford the compound **G-C10** as orange solid (537.15 mg, 60%). **^{1}H NMR** (600 MHz, $CDCl_3$) δ 7.92 (s, 1H), 7.86 (d, *J* = 3.7 Hz, 1H), 7.77 (d, *J* = 7.6 Hz, 1H), 7.64 – 7.49 (m, 3H), 7.44 – 7.33 (m, 4H), 4.06 (dt, *J* = 12.9, 6.5 Hz, 6H), 1.89 – 1.80 (m, 4H), 1.80 – 1.72 (m, 2H), 1.49 (dt, *J* = 15.3, 7.5 Hz, 6H), 1.40 – 1.23 (m, 36H), 0.90 – 0.86 (m, 9H). **^{13}C NMR** (151 MHz, $CDCl_3$) δ 191.98 (s), 166.42 (s), 164.82 (s), 153.04 (s), 152.22 (s), 151.16 (s), 144.01 (s), 143.33 (s), 141.20 (s), 135.99 (s), 135.86 (s), 135.30 (s), 134.09 (s), 132.41 (s), 131.70 (s), 128.06 (s), 124.45 (s), 123.22 (s), 122.00 (s), 121.51 (s), 121.01 (s), 118.66 (s), 108.63 (s), 73.65 (s), 69.32 (s), 31.97 (s), 31.95 (s), 30.38 (s), 29.79 (s), 29.76 (s), 29.73 (s), 29.69 (s), 29.66 (s), 29.61 (s), 29.43 (s), 29.39 (s), 29.38 (s), 29.32 (s), 26.11 (s), 26.08 (s), 22.73 (s), 22.71 (s), 14.14 (s). **ESI-HR-MS**: *m/z* for $C_{55}H_{74}O_8SNa^+$, ([M]+Na^+), calc.: 917.4997, found: 917.4966.

7.8 Synthesis of compound F-C12

A solution of 3,4,5-tris(dodecyloxy)benzoic acid (9.15 g, 13.55 mmol), **E** compound (3.7 g, 13.55 mmol), EDC·HCl (3.9 g, 20.3 mmol) and DMAP (622 mg, 5.42 mmol) in DCM (68 ml) was stirred for 16 h at r.t. Extra solvent - DCM (100 ml) was added, and the mixture was washed with water (3×50 ml). The organic phase was dried over $MgSO_4$. After filtration, the solvent was removed under vacuum and the crude was purified by SGCC (PE/EA = 50:1) to obtain the compound **F-C12** as a yellow solid (10.74 g, 85%). **^{1}H NMR** (600 MHz, $CDCl_3$) δ 7.78 (s, 1H), 7.63 (d, *J* = 7.9 Hz, 1H), 7.56 (d, *J* = 7.9 Hz, 1H), 7.50 (s, 1H), 7.44 – 7.37 (m, 3H), 7.33 (d, *J* = 8.0 Hz, 1H), 4.06 (dt, *J* = 12.8, 6.4 Hz, 6H), 1.91 – 1.80 (m, 4H), 1.80 – 1.72 (m, 2H), 1.49 (dt, *J* = 15.0, 7.5 Hz, 6H), 1.41 – 1.22 (m, 48H), 0.88 (dd, *J* = 11.1, 6.6 Hz, 9H). **^{13}C NMR** (151 MHz, $CDCl_3$) δ 191.27 (s), 164.73 (s), 153.04 (s), 152.16 (s), 143.32 (s), 142.60 (s), 141.02 (s), 137.39 (s), 136.07 (s), 135.25 (s), 128.04 (s), 127.82 (s), 123.22 (s), 122.89 (s), 121.74 (s), 121.30 (s), 118.68 (s), 108.62 (s), 73.64 (s), 69.31 (s), 31.96 (s), 31.95 (s), 30.37 (s), 29.78 (s), 29.76 (s), 29.75 (s), 29.72 (s), 29.68 (s), 29.66 (s), 29.59 (s), 29.42 (s), 29.39 (s), 29.30 (s), 26.10 (s), 26.07 (s), 22.71 (s), 14.14 (s). **ESI-HR-MS**: *m/z* for $C_{56}H_{84}BrO_6^+$, ([M]+H^+), calc. 931.5446, found: 931.5438.

7.9 Synthesis of compound G-C12

Under N_2 atmosphere, a mixture of 7-bromo-9-oxo-9H-fluoren-2-yl 3,4,5-tris (dodecyloxy)benzoate (931 mg, 1 mmol), 2-carboxythiophene-5-boronic acid (260 mg, 1 mmol), $Pd(PPh_3)_2Cl_2$ (175 mg), 2M Na_2CO_3 solution (2 ml) and THF (10 ml) was heated at 80 °C for 36 h. After cooling to r.t., the reaction mixture was poured into water and then acidified with 2M HCl to pH = 3, wherein the brown precipitate was appeared. The precipitate was isolated and dissolved in chloroform. The organic phase was dried over anhydrous Na_2SO_4, filtered and the solved was evaporated. The crude product was purified by SGCC (PE/EA =

50:1) to afford orange solid (0.54 mg, 55%). **^{1}H NMR** (600 MHz, $CDCl_3$) δ 7.88 (d, *J* = 31.6 Hz, 2H), 7.76 (s, 1H), 7.55 (d, *J* = 25.4 Hz,3H), 7.39 (d, *J* = 14.5 Hz,4H), 4.05 (s, 6H), 1.80 (d, *J* = 41.2 Hz, 6H), 1.49 (s, 6H), 1.31 (d, *J* = 51.4 Hz, 48H), 0.88 (s,9H). **^{13}C NMR** (151 MHz, $CDCl_3$) δ 190.95 (s), 165.57 (s), 163.79 (s), 152.01 (s), 151.16 (s), 150.02 (s), 142.92 (s), 142.28 (s), 140.14 (s), 134.89 (s), 134.79 (s), 134.22 (s), 133.03 (s), 131.30 (s), 130.81 (s), 127.01 (s), 123.37 (s), 122.20 (s), 120.89 (s), 120.47 (s), 119.95 (s), 117.59 (s), 107.59 (s), 72.63 (s), 68.29 (s), 30.93 (s), 29.35 (s), 28.70 (s), 28.65 (s), 28.40 (s), 28.38 (s), 28.30 (s), 25.09 (s), 21.69 (s), 13.12 (s). **ESI-HR-MS**: *m/z* for $C_{61}H_{86}O_8SNa^+$, ([M]+Na^+), calc.1001.5936, found: 1001.5918.

7.10 Synthesis of compound L-C10

A mixture of 5-bromo-1,2,3-tris(decyloxy)benzene (6.26 g, 10 mmol), (4-hydroxyphenyl)boronic acid (1.52 g, 11 mmol) and THF (50 ml) was added tetrakis(triphenylphosphine)palladium (0.115 mg, 0.1 mmol) catalyst under nitrogen atmosphere. 2M Na_2CO_3 solution (10 ml) was added dropwise at this mixture and this was stirred under refluxed for 24 h. After cooling to r.t., the reaction was quenched by the addition of 30% of hydrogen peroxide solution (3 ml). Subsequently, 2M NaOH solution (30 ml) was added and the reaction mixture was extracted with EA. The organic phase was concentrated and purified by SGCC (DCM/PE = 5:1) to afford the compound **L-C10** as a white solid (5.11 g, 80%). **^{1}H NMR** (600 MHz, $CDCl_3$) δ 7.39 (d, *J* = 8.2 Hz,2H), 6.87 (d, *J* = 8.2 Hz, 2H), 6.69 (s, 2H), 5.68 (s, 1H), 4.01 (dd, *J* = 14.3, 6.9 Hz, 6H), 1.80 (ddd, *J* = 21.1, 14.2, 7.0 Hz, 6H), 1.47 (d, *J* = 6.6 Hz, 6H), 1.38 – 1.05 (m, 36H), 0.88 (t, *J* = 6.8 Hz, 9H). **^{13}C NMR** (151 MHz, $CDCl_3$) δ 155.18 (s), 153.09 (s), 136.94 (s), 136.40 (s), 133.76 (s), 128.04 (s), 115.48 (s), 105.37 (s), 73.63 (s), 69.09 (s), 31.83 (s), 31.80 (s), 30.16 (s), 29.63 (s), 29.56 (s), 29.54 (s), 29.49 (s), 29.32 (s), 29.29 (s), 29.25 (s), 26.01 (s), 22.59 (s), 22.57 (s). **ESI-HR-MS**: *m/z* for $C_{42}H_{70}O_4K^+$, ([M]+K^+). calc.:677.4906, found: 677.4905.

7.11 Synthesis of compound L-C12

To a mixture of 5-bromo-1,2,3-tris(dodecyloxy)benzene (7.09 g, 10 mmol), (4-hydroxyphenyl)boronic acid (1. 52 g, 11 mmol) and THF (50 ml) was added tetrakis(triphenylphosphine)palladium (0.115 mg, 0.1 mmol) catalyst under nitrogen. Then 2M Na_2CO_3 solution (10 ml) was added dropwise. The resulting mixture was refluxed 24 h. After cooling to r.t., the reaction was quenched by the addition of 30% hydrogen peroxide solution (3 ml). Subsequently, 2M NaOH (30 ml) and mixture was treated with EA (2X50 ml). The organic phases were separated, dried over anhydrous MgSO4 and concentrated. The crude product was purified by SGCC (DCM/PE = 5:1) to obtain the compound **L-C12** as a white solid (5.79 g, 80%). **^{1}H NMR** (600 MHz, $CDCl_3$) δ 7.41 (d, *J* = 8.5 Hz, 2H), 6.87 (d, *J* = 8.4 Hz,2H), 6.69 (s,2H), 4.86 (s, 1H), 4.00 (dt, *J* = 25.4, 6.5 Hz, 6H), 1.85 – 1.79 (m, 4H), 1.76 (dd, *J* = 14.7, 7.0 Hz, 2H), 1.47 (m, *J* = 13.6, 6.4 Hz, 6H), 1.39 – 1.19 (m, 48H), 0.88 (t, *J* = 6.9 Hz, 9H).**^{13}C NMR** (151 MHz, $CDCl_3$) δ 154.92 (s), 153.27 (s), 137.44 (s), 136.19 (s), 134.25 (s), 128.23 (s), 115.49 (s), 105.59 (s), 73.57 (s), 69.22 (s), 31.94 (s), 31.92 (s), 30.34 (s), 29.75 (s), 29.73 (s), 29.70 (s), 29.65 (s), 29.64 (s), 29.45 (s), 29.42 (s), 29.39 (s), 29.36 (s), 26.14 (s), 26.11 (s), 22.69 (s), 14.11 (s). **ESI-HR-MS**: *m/z* for $C_{48}H_{83}O_4K^+$, ([M]+H^+), calc. 723.6286, found: 723.6277.

7.12 Synthesis of compound R3-C10

The 3',4',5'-tris(decyloxy)-[1,1'-biphenyl]-4-ol (1.92 g, 3 mmol) and (R)-3-hydroxybutanoic acid (312.3 mg, 3 mmol) was placed in 100 ml round-bottom flask and DCM (15 ml) was added. EDC·HCl (863 mg, 4.5 mmol) and DMAP (147 mg, 1.2 mmol) were added at 0 °C and stirred for 20 h at r.t. The resulted mixture was diluted with extra DCM solvent (20 ml), and then successively washed with water (3×20 ml), 10% NaOH solution (10 ml) and water (2×15 ml). The organic phase was dried over anhydrous Na_2SO_4, filtered and concentrated. The crude product was purified by SGCC (DCM/PE = 10:1) to afford pure product **R3-C10** as colorless oil liquid (1.85 g, 85%).^{1}**H NMR** (600 MHz, $CDCl_3$) δ 7.54 (d, *J* = 7.5 Hz, 2H), 7.14 (d, *J* = 7.5 Hz, 2H), 6.72 (s, 2H), 4.35 (d, *J* = 1.7 Hz, 1H), 4.01 (dt, *J* = 11.7, 5.6 Hz, 6H), 2.75 (dt, *J* = 16.6, 12.6 Hz, 2H), 1.97 – 1.67 (m, 6H), 1.49 (d, *J* = 5.8 Hz, 6H), 1.41 – 1.14 (m, 40H), 0.88 (d, *J* = 6.3 Hz, 9H).^{13}**C NMR** (151 MHz, $CDCl_3$) δ 171.49 (s), 153.38 (s), 149.52 (s), 139.57 (s), 138.05 (s), 135.63 (s), 128.12 (s), 121.65 (s), 106.01 (s), 73.58 (s), 69.28 (s), 64.29 (s), 42.98 (s), 31.97 (s), 31.94 (s), 30.39 (s), 29.78 (s), 29.71 (s), 29.68 (s), 29.65 (s), 29.62 (s), 29.47 (s), 29.45 (s), 29.38 (s), 26.17 (s), 26.14 (s), 22.73 (s), 22.71 (s), 22.55 (s), 14.14 (s).**ESI-HR-MS**: *m/z* for $C_{46}H_{76}O_6Na^+$, ([M]+Na$^+$), calc.: 747.5534, found: 66 747.5534.

7.13 Synthesis of compound R3-C12

To the 3',4',5'-tris(dodecyloxy)-[1,1'-biphenyl]-4-ol (2.17 g, 3 mmol) and (R)-3-hydroxybutanoic acid (312.3 mg, 3 mmol) placed in 100 ml round-bottom flask was added DCM (15 ml). EDC·HCl (863 mg, 4.5 mmol) and DMAP (147 mg, 1.2 mmol) were added at 0 °C and stirred for 20 h at r.t. The mixture was diluted with extra solvent, and then successively washed with water (3×20 ml), 10% NaOH solution (10 ml) and water (2×15 ml). The organic phase was dried over anhydrous Na_2SO_4, filtered and concentrated. The crude solid was purified by SGCC (DCM/PE = 10:1) to afford the pure product **R3-C12** as colorless oil liquid (2.06 g, 85%). ^{1}**H NMR** (600 MHz, $CDCl_3$) δ 7.53 (d, *J* = 8.5 Hz, 2H), 7.13 (d, *J* = 8.5 Hz, 2H), 6.71 (s, 2H), 4.51 – 4.23 (m, 1H), 4.00 (dt, *J* = 21.9, 6.5 Hz, 6H), 2.75 (qd, *J* = 16.7, 6.1 Hz, 2H), 1.80 (ddd, *J* = 24.0, 14.4, 7.1 Hz, 6H), 1.52 – 1.44 (m, 6H), 1.39 – 1.23 (m, 52H), 0.88 (t, *J* = 7.0 Hz, 9H). ^{13}**C NMR** (151 MHz, $CDCl_3$) δ 171.50 (s), 153.38 (s), 149.50 (s), 139.58 (s), 138.05 (s), 135.63 (s), 128.12 (s), 121.64 (s), 106.00 (s), 73.58 (s), 69.28 (s), 64.29 (s), 42.96 (s), 31.97 (s), 31.95 (s), 30.39 (s), 29.79 (s), 29.77 (s), 29.73 (s), 29.68 (s), 29.46 (s), 29.45 (s), 29.42 (s), 29.39 (s), 26.17 (s), 26.14 (s), 22.72 (s), 22.54 (s), 14.14 (s). **ESI-HR-MS**: *m/z* for $C_{52}H_{89}O_6^+$, ([M]+H$^+$), calc.: 809.6654, found: 66 809.6632.

7.14 Synthesis of compound S3-C10

To 3',4',5'-tris(decyloxy)-[1,1'-biphenyl]-4-ol (1.92 g, 3 mmol) and (S)-3-hydroxybutanoic acid (312.3 mg, 3 mmol) placed in 100 ml round-bottom flask was added DCM (15 ml), EDC·HCl (863 mg, 4.5 mmol) and DMAP (147 mg, 1.2 mmol) were added at 0 °C and stirred for 20 h at r.t. The mixture was diluted with extra solvent, and then successively washed with water (3×20 ml), 10% NaOH solution (10 ml) and water (2×15 ml). The organic phase was dried over anhydrous Na_2SO4. The solvent was evaporated and the crude product was purified by SGCC (DCM/PE = 10:1) to afford the product **S3-C10** as colorless oil liquid (1.89 g, 87%). ^{1}**H NMR** (600 MHz, $CDCl_3$) δ 7.54 (d, *J* = 7.5 Hz, 2H), 7.14 (d, *J* = 7.5 Hz, 2H), 6.72 (s, 2H), 4.35 (d, *J* = 1.7 Hz, 1H), 4.01 (dt, *J* = 11.7, 5.6 Hz, 6H), 2.75 (dt, *J* = 16.6, 12.6 Hz, 2H), 1.97 – 1.67 (m, 6H), 1.49 (d, *J* = 5.8 Hz, 6H), 1.41 – 1.14 (m, 40H), 0.88 (d, *J* = 6.3 Hz, 9H).^{13}**C NMR** (151 MHz,

$CDCl_3$) δ 171.49 (s), 153.38 (s), 149.52 (s), 139.57 (s), 138.05 (s), 135.63 (s), 128.12 (s), 121.65 (s), 106.01 (s), 73.58 (s), 69.28 (s), 64.29 (s), 42.98 (s), 31.97 (s), 31.94 (s), 30.39 (s), 29.78 (s), 29.71 (s), 29.68 (s), 29.65 (s), 29.62 (s), 29.47 (s), 29.45 (s), 29.38 (s), 26.17 (s), 26.14 (s), 22.73 (s), 22.71 (s), 22.55 (s), 14.14 (s). **ESI-HR-MS**: *m/z* for $C_{46}H_{76}O_6K^+$, ([M]+K$^+$), calc.: 763.5274, found: 66 763.5273.

7.15 Synthesis of compound N3-C10

To the mixture of 3',4',5'-tris(decyloxy)-[1,1'-biphenyl]-4-ol (1.92 g, 3 mmol) and 4-hydroxybutanoic acid (270.2 mg, 3 mmol) in 100 ml round-bottom flask was added DCM (15 ml). EDC·HCl (863 mg, 4.5 mmol) and DMAP (147 mg, 1.2 mmol) were added to this at 0 °C and stirred for 20 h at r.t. The mixture was diluted with extra solvent, and then successively washed with water (3×20 ml), 10% NaOH solution (10 ml) and water (2×15 ml). The organic phase was dried over anhydrous Na_2SO_4. The solvent was evaporated and crude product was purified by SGCC (DCM/PE = 10:1) to afford the product **N3-C10** as colorless oil liquid (1.86 g, 87%). ^{1}H NMR (600 MHz, $CDCl_3$) δ 7.54 (d, *J* = 8.6 Hz, 2H), 7.14 (d, *J* = 8.6 Hz, 2H), 6.71 (s, 2H), 4.03 – 3.96 (m, 8H), 2.87 (t, *J* = 5.6 Hz, 2H), 1.87 – 1.79 (m, 4H), 1.76 (dd, *J* = 14.8, 7.0 Hz, 2H), 1.53 – 1.45 (m, 6H), 1.34 – 1.25 (m, 37H), 0.88 (t, *J* = 7.0 Hz, 9H).^{13}C NMR (151 MHz, $CDCl_3$) δ 171.53 (s), 153.36 (s), 149.55 (s), 139.53 (s), 138.00 (s), 135.63 (s), 128.11 (s), 121.63 (s), 105.98 (s), 73.58 (s), 69.27 (s), 58.17 (s), 36.99 (s), 31.96 (s), 31.92 (s), 30.37 (s), 29.77 (s), 29.70 (s), 29.66 (s), 29.64 (s), 29.61 (s), 29.45 (s), 29.43 (s), 29.37 (s), 26.15 (s), 26.13 (s), 22.72 (s), 22.70 (s), 14.13 (s). **ESI-HR-MS**: *m/z* for $C_{45}H_{74}O_6K^+$, ([M]+K$^+$), calc.: 749.5117, found: 749.5109.

7.16 Synthesis of compound *S*-C10

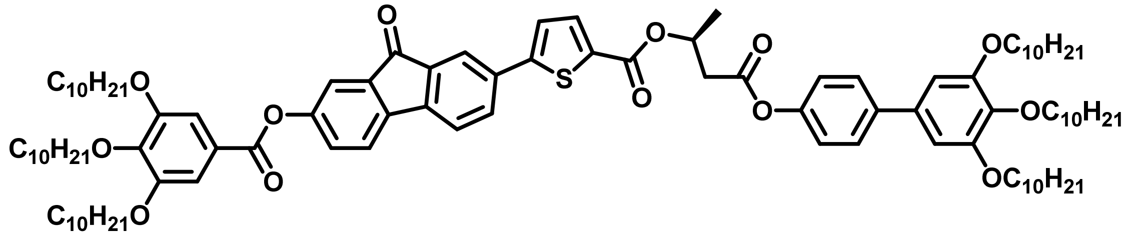

The mixture of acid **G-C10** (895 mg, 1 mmol) and $SOCl_2$ (5 ml) was refluxing for 30 min. The excess of $SOCl_2$ was removed under vacuum, and then dry pyridine (0.5 ml), DCM (5 ml) and 3',4',5'-tris(decyloxy)-[1,1'-biphenyl]-4-yl (S)-3-hydroxybutanoate (725.11 mg, 1 mmol) were added. This new mixture was stirred at r.t. overnight. The extra solvent DCM (50 ml) was added and the organic phase was separated. This was washed with saturated aq NaCl (2×20 ml), dried over anhydrous $MgSO_4$, concentrated under vacuum to afford the crude product. This was purified by SGCC (DCM/PE = 10:1) to obtain ***S*-C10** as a yellow solid (1 g, 65%). **^{1}H NMR** (600 MHz, $CDCl_3$) δ 7.94 (s, 1H), 7.81 (d, *J* = 3.0 Hz, 1H), 7.77 (d, *J* = 7.7 Hz, 1H), 7.60 (d, *J* = 8.0 Hz, 1H), 7.56 (d, *J* = 7.7 Hz, 1H), 7.54 – 7.46 (m, 3H), 7.40 (s, 2H), 7.36 (d, *J* = 7.6 Hz, 2H), 7.12 (d, *J* = 8.0 Hz, 2H), 6.69 (s, 2H), 5.69 – 5.60 (m, 1H), 4.06 (dt, *J* = 12.5, 6.4 Hz, 6H), 3.99 (dt, *J* = 13.2, 6.4 Hz, 6H), 3.06 (dd, *J* = 15.4, 7.6 Hz, 1H), 2.92 (dd, *J* = 15.5, 5.1 Hz, 1H), 1.88 – 1.68 (m, 12H), 1.56 (d, *J* = 6.2 Hz, 3H), 1.52 – 1.42 (m, 12H), 1.39 – 1.17 (m, 72H), 0.88 (d, *J* = 5.5 Hz, 18H). **^{13}C NMR** (151 MHz, CDCl3) δ 192.01 (s), 168.74 (s), 164.75 (s), 161.20 (s), 153.34 (s), 153.05 (s), 152.20 (s), 149.83 (s), 149.65 (s), 143.86 (s), 143.32 (s), 141.23 (s), 139.48 (s), 137.99 (s), 135.87

(s), 135.69 (s), 135.33 (s), 134.66 (s), 134.29 (s), 132.75 (s), 132.33 (s), 128.10 (s), 128.03 (s), 124.24 (s), 123.24 (s), 122.00 (s), 121.62 (s), 121.44 (s), 120.98 (s), 118.64 (s), 108.62 (s), 105.98 (s).73.64 (s), 73.56 (s), 69.31 (s), 69.25 (s), 68.45 (s), 41.11 (s), 31.97 (s), 31.93 (s), 30.38 (s), 29.77 (s), 29.76 (s), 29.70 (s), 29.66 (s), 29.61 (s), 29.59 (s), 29.46 (s), 29.42 (s), 29.38 (s), 29.31 (s), 26.16 (s), 26.13 (s), 26.11 (s), 26.07 (s), 22.73 (s), 22.71 (s), 20.16 (s), 14.14 (s). **ESI-HR-MS**: *m/z* for $C_{101}H_{152}NO_{13}S^+$, ([M]+NH_4^+). calc.1619.09789, found: 1619.09213. **Elemental analysis** for $C_{101}H_{148}O_{13}S$, calc.: C 75.71%, H 9.31%, S 2.00%, found: C 75.57%, H 9.19%, S 1.81%.

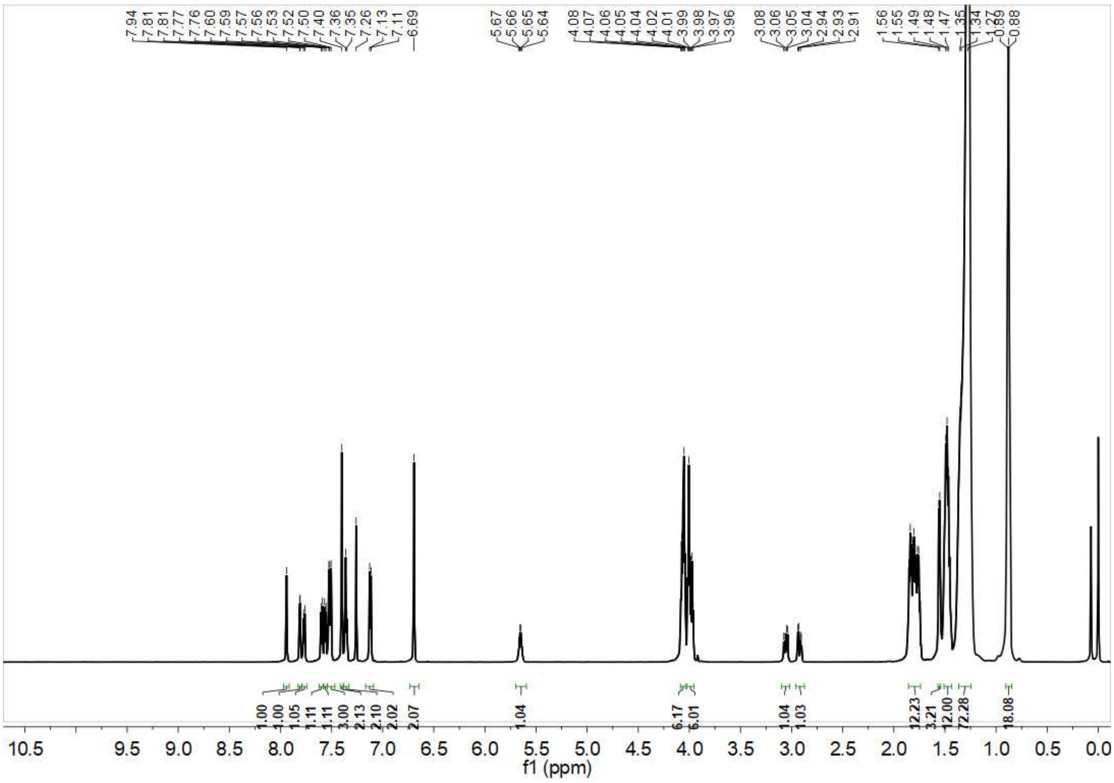

Figure S15. ^{1}H NMR spectrum of ***S*-C10** (600 MHz, $CDCl_3$, 298 K)

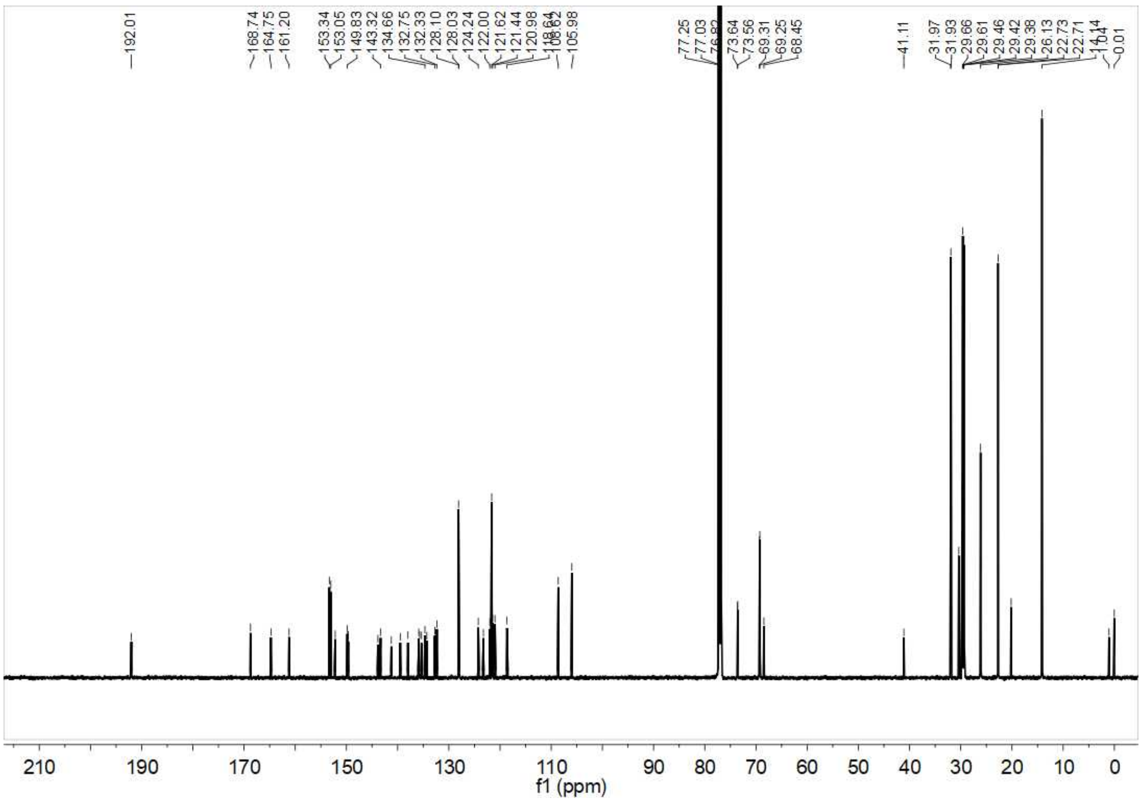

Figure S16. ^{13}C NMR spectrum of ***S*-C10** (150 MHz, $CDCl_3$, 298 K)

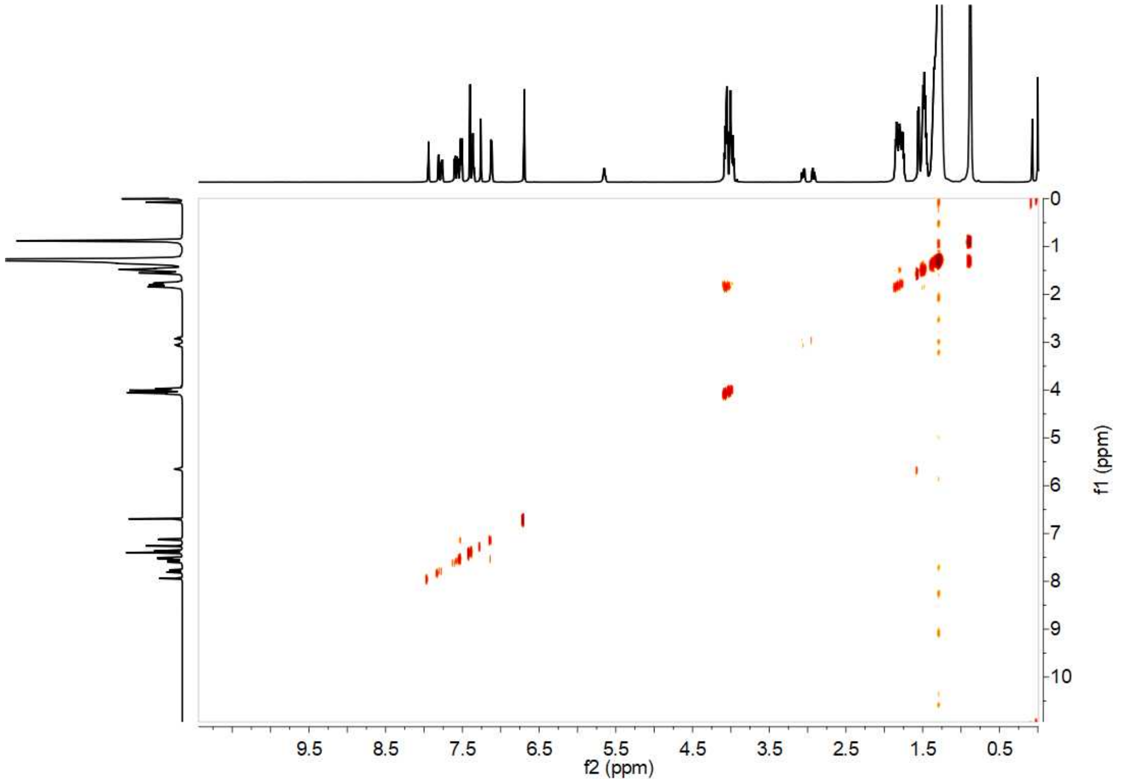

Figure S17. COSY spectrum of ***S*-C10** (150 MHz, $CDCl_3$, 298 K)

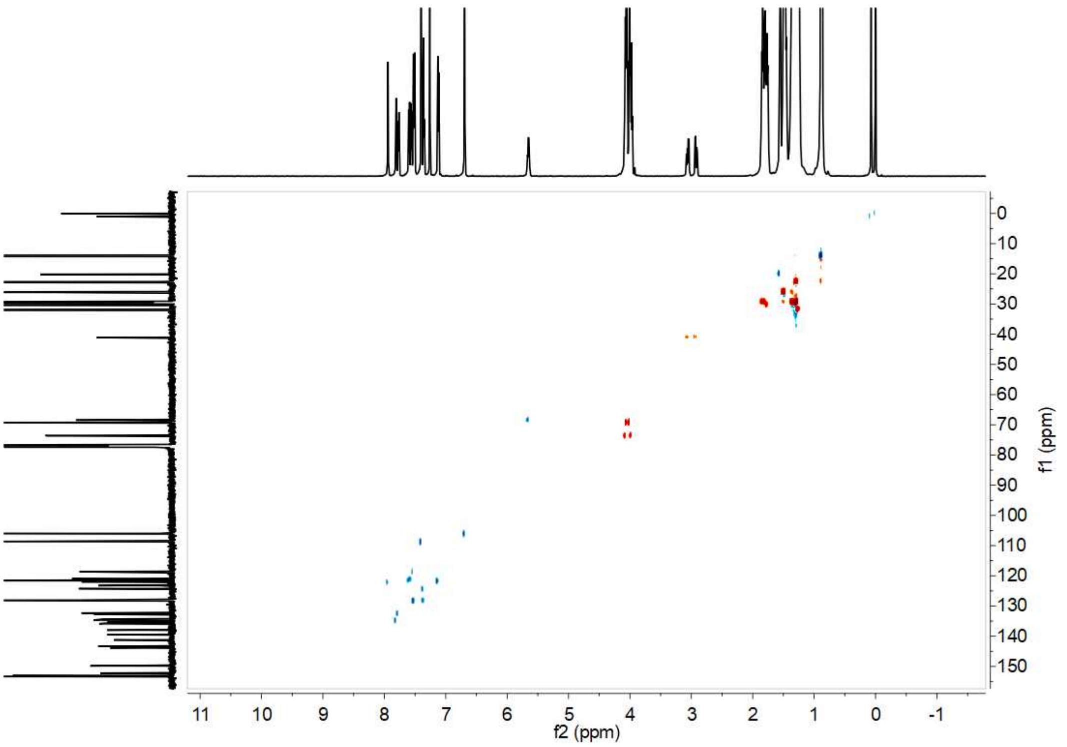

Figure S18. HSQC spectrum of ***S*-C10** (150 MHz, $CDCl_3$, 298 K)

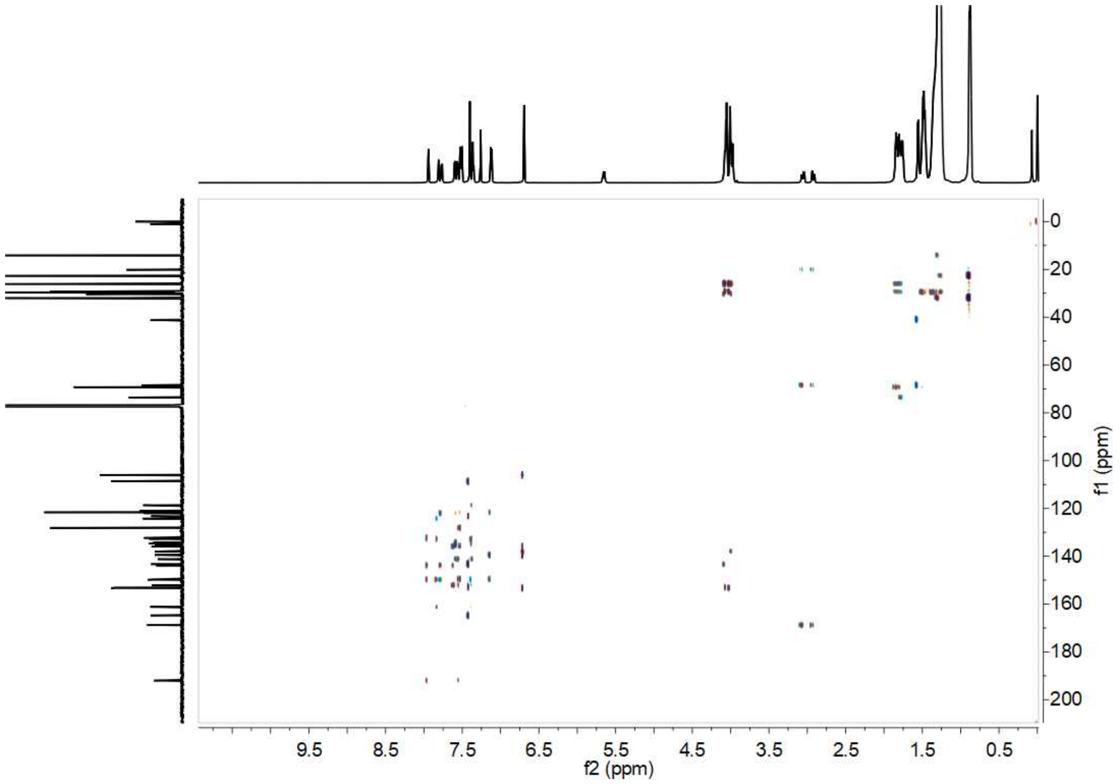

Figure S19. HMBC spectrum of ***S*-C10** (150 MHz, $CDCl_3$, 298 K)

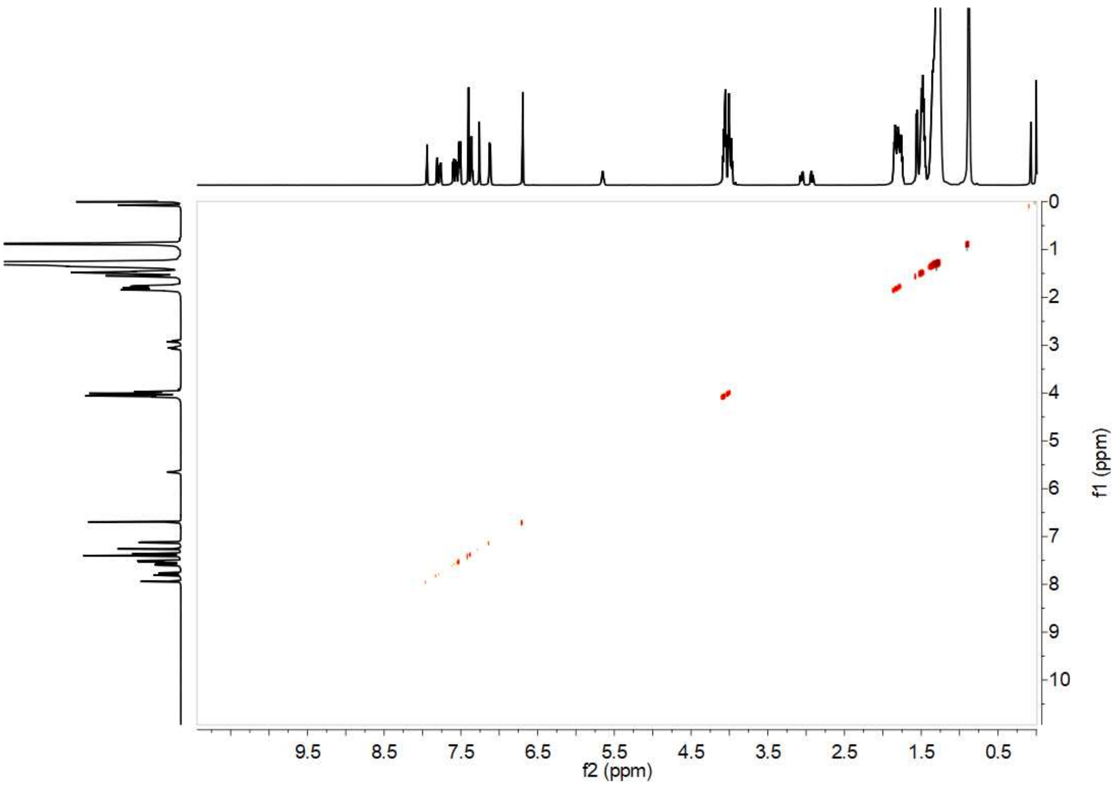

Figure S20. NOSY spectrum of ***S*-C10** (150 MHz, $CDCl_3$, 298 K)

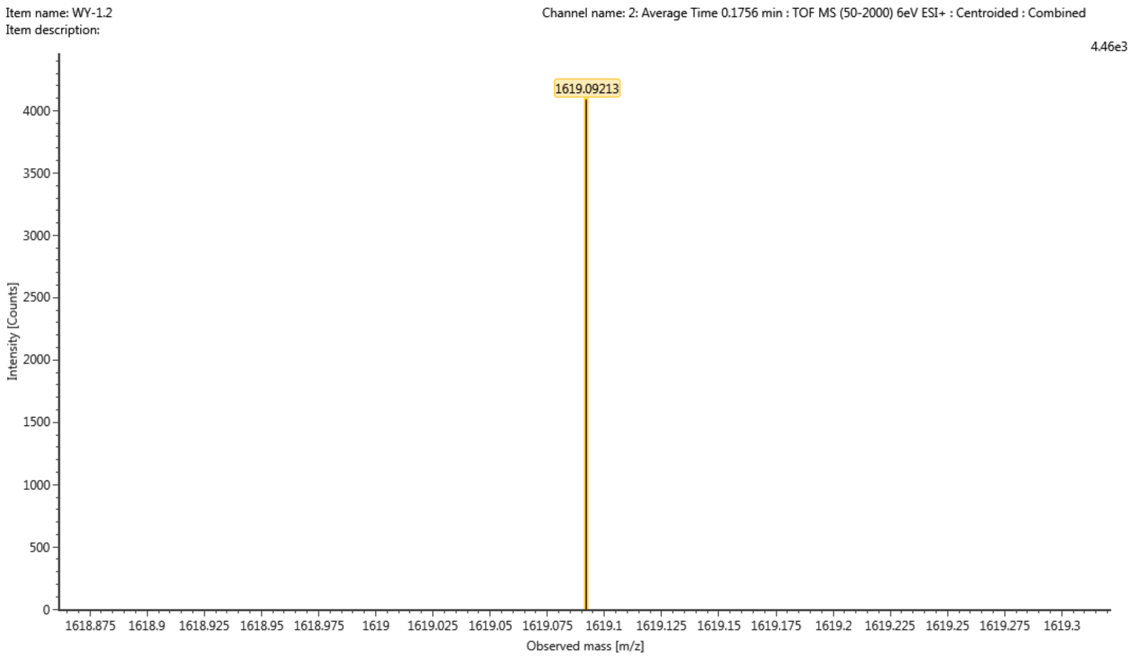

Figure S21. ESI-HR-MS spectrum of ***S*-C10**

7.17 Synthesis of compound *R*-C10

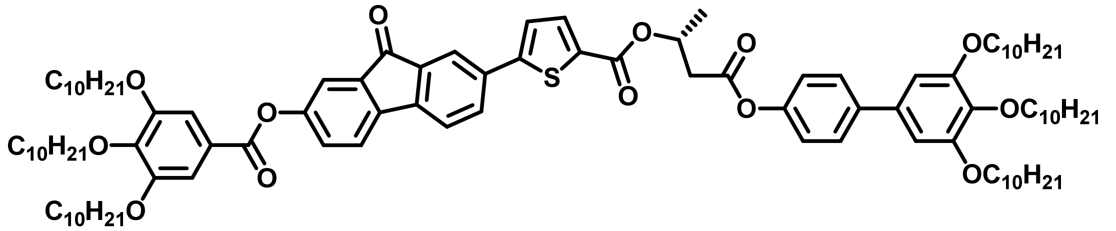

The mixture of acid **G- C10** (895 mg, 1 mmol) and $SOCl_2$ (5 ml) was refluxing for 30 min. The excess of $SOCl_2$ was removed under vacuum and a mixture of 3',4',5'-tris(decyloxy)-[1,1'-biphenyl]-4-yl (R)-3-hydroxybutanoate (725.11 mg, 1 mmol), pyridine (0.5 ml) and DCM (5 ml) was added. The resulting mixture was stirred overnight at r.t. An extra DCM (50 ml) was added and the organic phase was washed with saturated aq NaCl (2×20 ml), dried over anhydrous $MgSO_4$, filtered and the solvent was removed. The crude product was purified by SGCC (DCM/PE = 10:1) to obtain the compound ***R*-C10** as a yellow solid (1 g, 65%). ^{1}**H NMR** (600 MHz, $CDCl_3$) δ 7.94 (d, *J* = 1.4 Hz, 1H), 7.81 (d, *J* = 3.9 Hz, 1H), 7.77 (dd, *J* = 7.8, 1.6 Hz, 1H), 7.60 (d, *J* = 8.0 Hz, 1H), 7.57 (d, *J* = 7.8 Hz, 1H), 7.52 (dd, *J* = 10.3, 5.4 Hz, 3H), 7.40 (d, *J* = 5.3 Hz, 2H), 7.36 (dd, *J* = 9.9, 3.0 Hz, 2H), 7.12 (d, *J* = 8.6 Hz, 2H), 6.69 (s, 2H), 5.81 – 5.53 (m, 1H), 4.06 (dt, *J* = 12.7, 6.5 Hz, 6H), 3.99 (dt, *J* = 19.5, 6.6 Hz, 6H), 3.06 (dd, J = 15.5, 7.7 Hz, 1H), 2.92 (dd, J = 15.5, 5.4 Hz, 1H), 1.88 – 1.73 (m, 12H), 1.60 – 1.53 (m, 3H), 1.53 – 1.42 (m, 12H), 1.40 – 1.18 (m, 72H), 0.95 – 0.81 (m, 18H). ^{13}**C NMR** (151 MHz, CDCl3) δ 192.02 (s), 168.74 (s), 164.75 (s), 161.20 (s), 153.34 (s), 153.04 (s), 152.20 (s), 149.84 (s), 149.64 (s), 143.86 (s), 143.32 (s), 141.24 (s), 139.48 (s), 137.99 (s), 135.87 (s), 135.69 (s), 135.33 (s), 134.66 (s), 134.30 (s), 132.75 (s), 132.34 (s), 128.10 (s), 128.03 (s), 124.25 (s), 123.24 (s), 122.01 (s), 121.62 (s), 121.44 (s), 120.98 (s), 118.64 (s), 108.62 (s), 105.98 (s), 73.65 (s), 73.56 (s), 69.31 (s), 69.25 (s), 68.45 (s), 41.11 (s), 31.96 (s), 31.93 (s), 30.38 (s), 29.77 (s), 29.76 (s), 29.70 (s), 29.66 (s), 29.60 (s), 29.46 (s), 29.43 (s), 29.42 (s), 29.37 (s), 29.31 (s), 26.16 (s), 26.13 (s), 26.10 (s), 26.07 (s), 22.72 (s), 22.70 (s), 20.16 (s), 14.14 (s). **ESI-HR-MS**: *m/z* for $C_{101}H_{148}O_{13}SNa^+$, ([M]+ Na^+); calc.: 1624.05329, found: 1624.05081. **Elemental analysis**: for $C_{101}H_{148}O_{13}S$ calc.: C 75.71%, H 9.31%, S 2.00%, found: C 75.30%, H 9.17%, S 1.79 %.

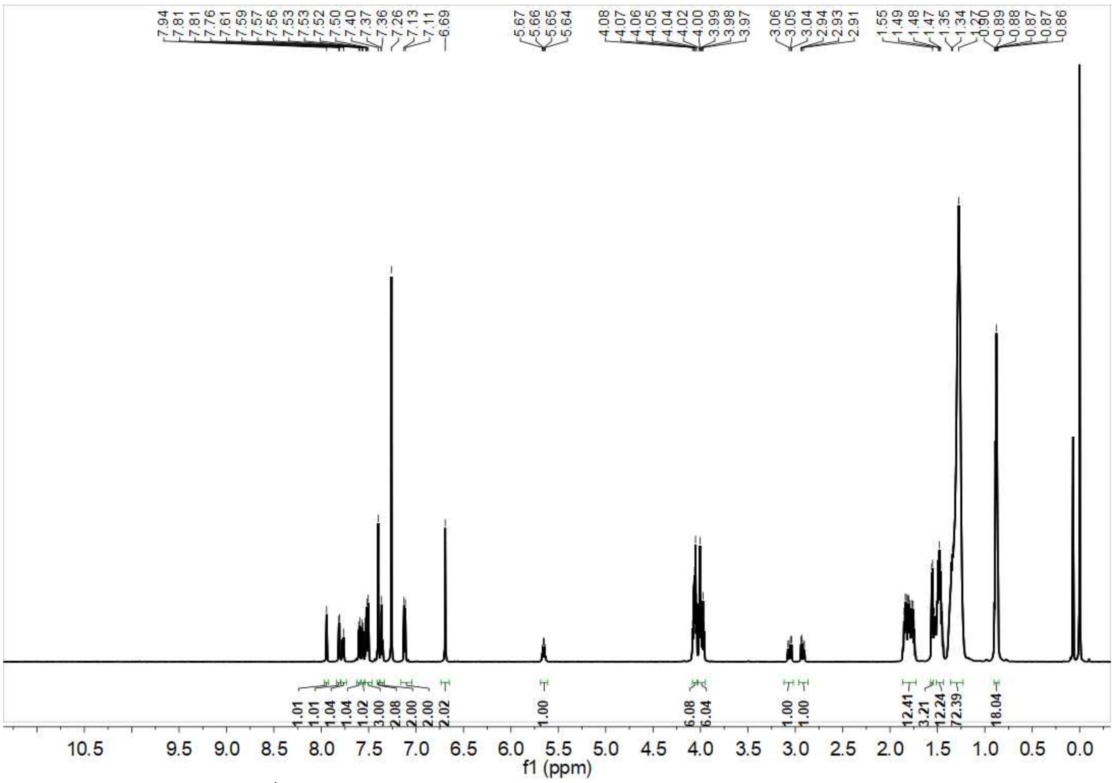

Figure S22. ^{1}H NMR spectrum of ***R*-C10** (600 MHz, $CDCl_3$, 298 K)

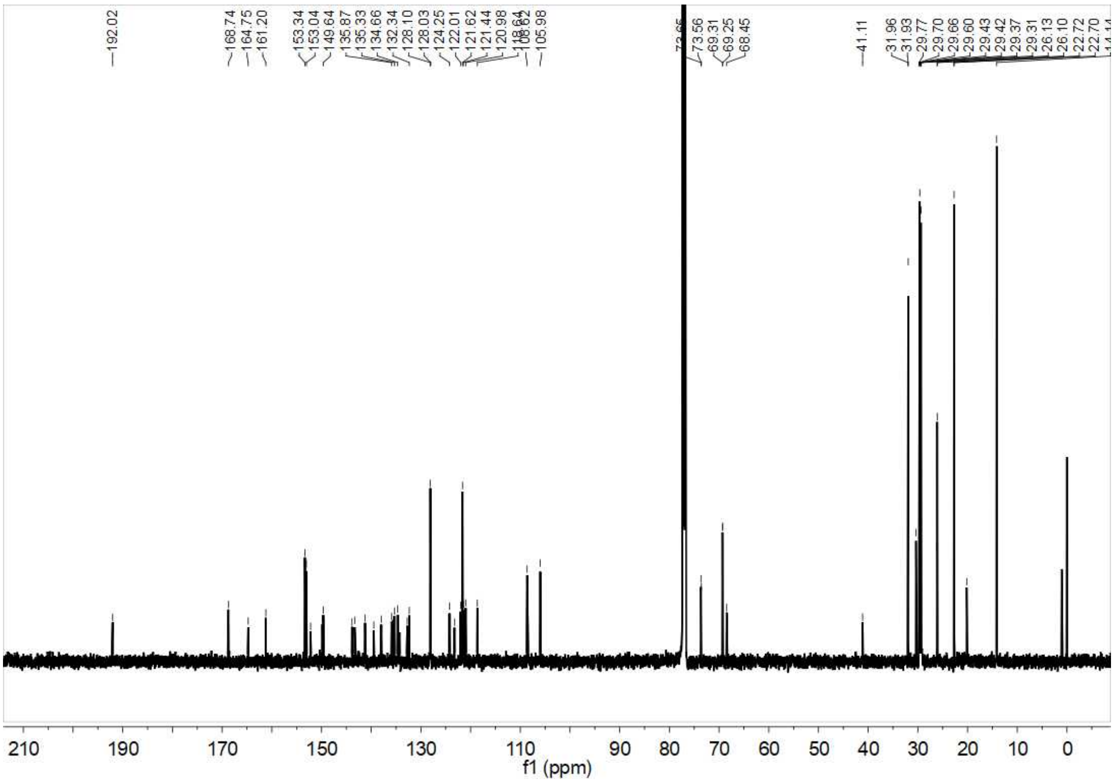

Figure S23. ^{13}C NMR spectrum of ***R*-C10** (150 MHz, $CDCl_3$, 298 K)

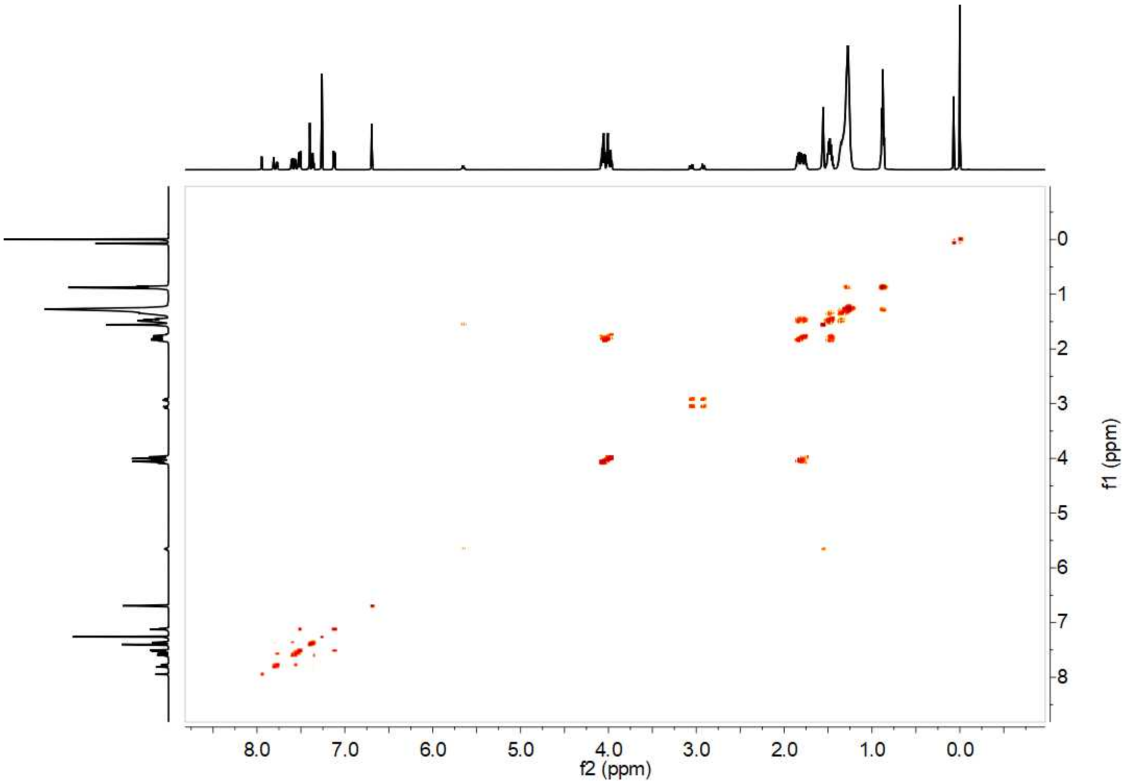

Figure S24. COSY spectrum of ***R*-C10** (150 MHz, $CDCl_3$, 298 K)

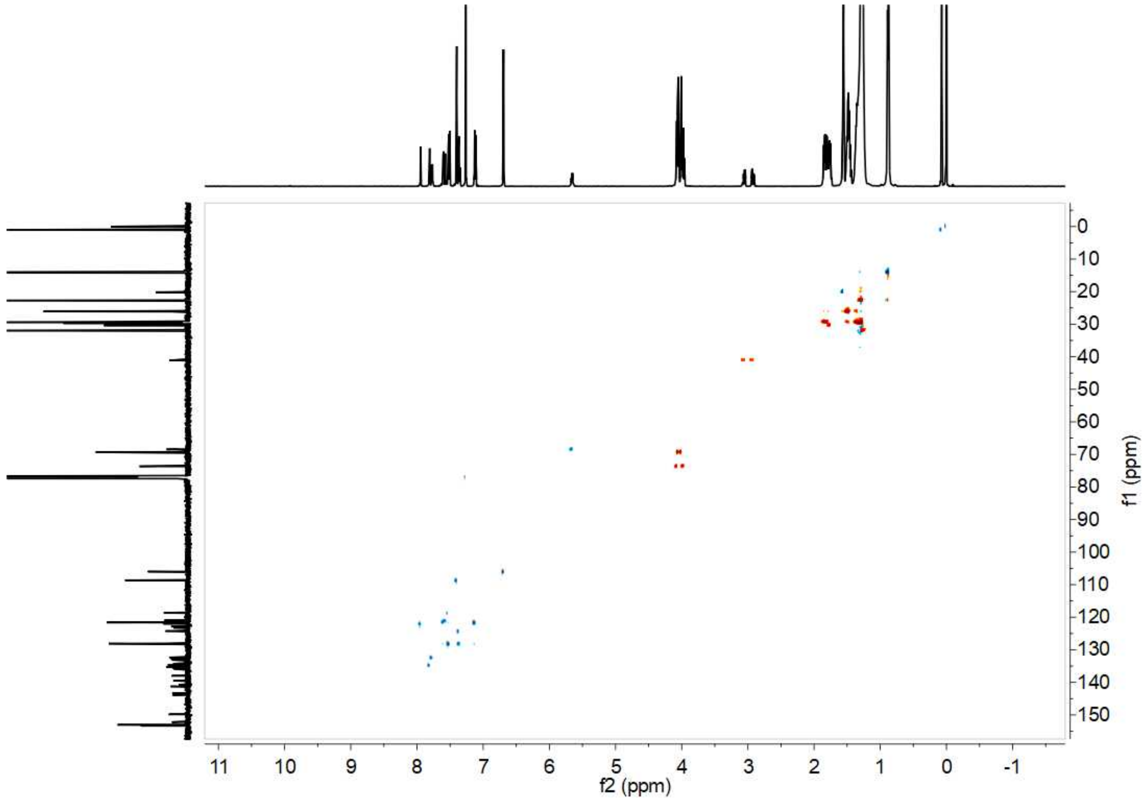

Figure S25. HSQC spectrum (150 MHz, $CDCl_3$, 298 K) recorded for ***R*-C10**

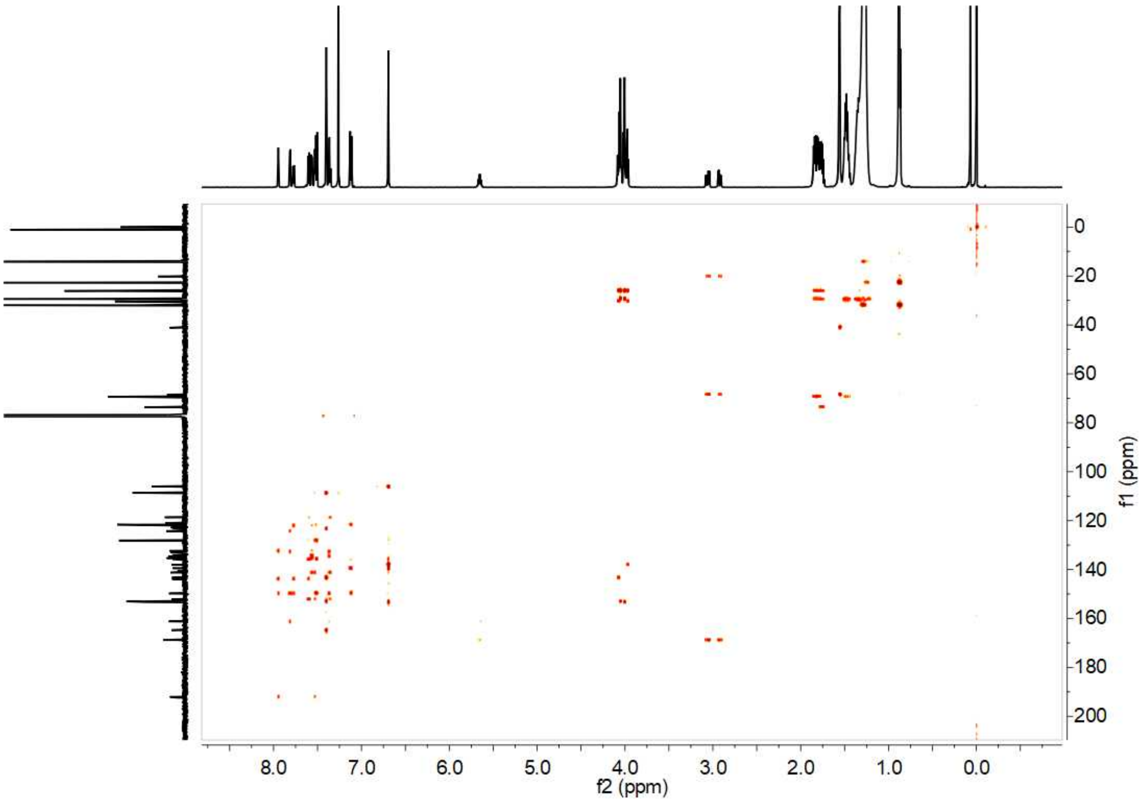

Figure S26. HMBC spectrum of ***R*-C10** (150 MHz, $CDCl_3$, 298 K)

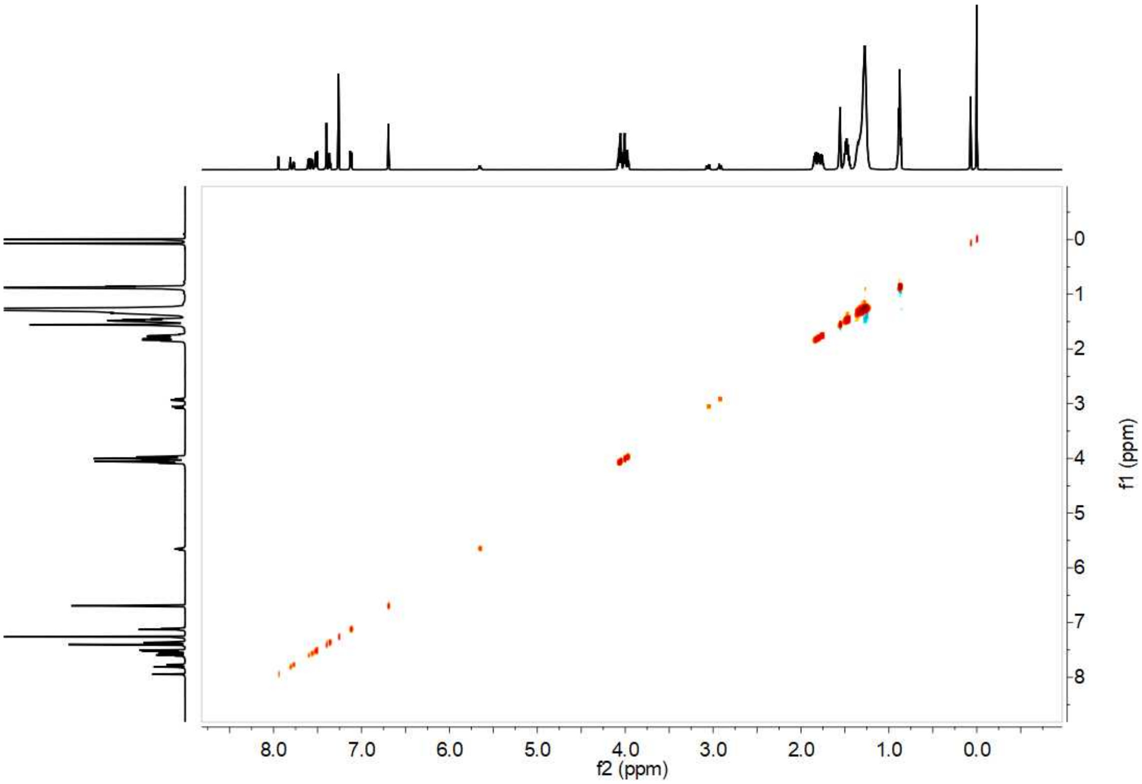

Figure S27. NOSY spectrum of ***R*-C10** (150 MHz, $CDCl_3$, 298 K)

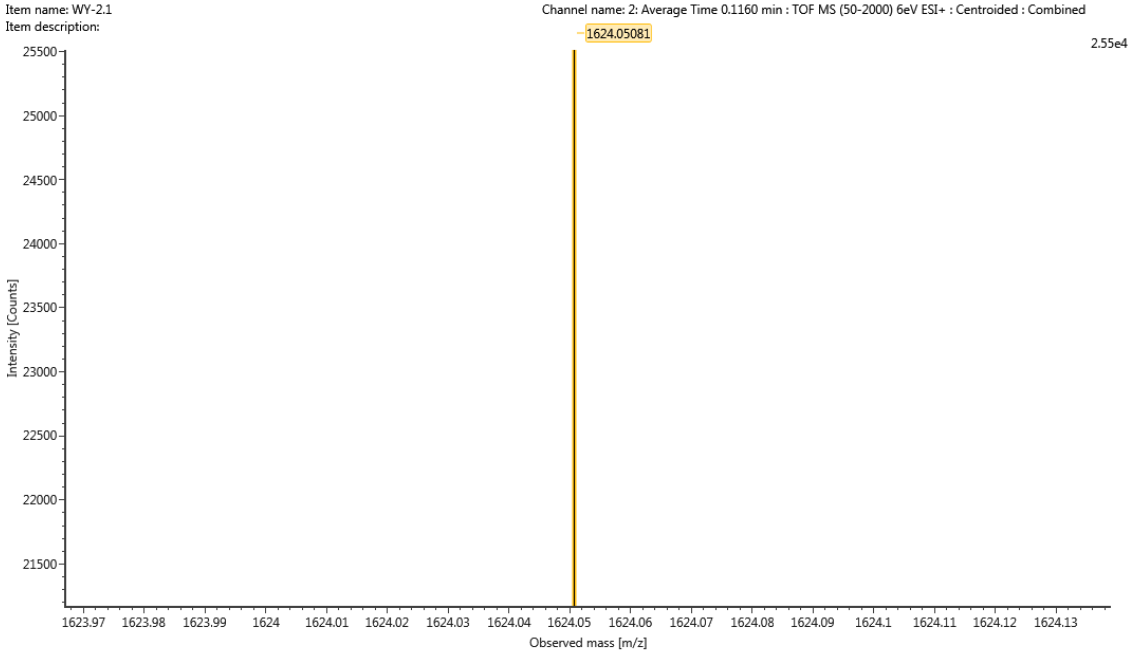

Figure S28. ESI-HR-MS spectrum of ***R*-C10**

7.18 Synthesis of compound *R*-C12

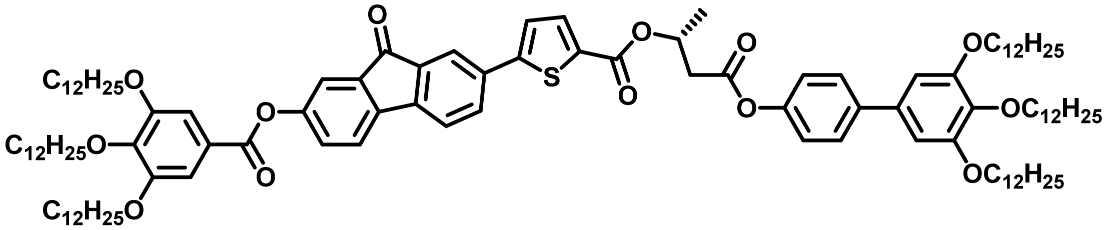

The acid **G-C12** (978.60 mg, 1 mmol) and $SOCl_2$ (5 ml) was refluxing for 30 min. The excess of $SOCl_2$ was removed under vacuum, and the mixture of dry pyridine (0.5 ml), DCM (5 ml) and 3',4',5'-tris(dodecyloxy)-[1,1'-biphenyl]-4-yl (R)-3-hydroxybutanoate (808.6 g, 1 mmol) were added. The resulting mixture was stirred at r.t. overnight. An extra DCM (50 ml) was added and the organic phase was washed with saturated aq NaCl (2 × 20 ml), dried over anhydrous $MgSO_4$, filtered and concentrated. The crude product was purified by SGCC (DCM/PE = 10:1) to obtain the compound ***R*-C12** as a yellow solid (1.15 g, 65%). **^{1}H NMR** (600 MHz, $CDCl_3$) δ 7.94 (s, 1H), 7.81 (d, *J* = 3.9 Hz, 1H), 7.77 (dd, *J* = 7.8, 1.5 Hz, 1H), 7.58 (dd, *J* = 20.3, 7.9 Hz, 2H), 7.52 (dd, *J* = 10.1, 5.3 Hz, 3H), 7.41 (d, *J* = 12.4 Hz, 2H), 7.36 (dd, *J* = 9.6, 3.0 Hz, 2H), 7.12 (d, *J* = 8.6 Hz, 2H), 6.69 (s, 2H), 5.74 – 5.53 (m, 1H), 4.06 (dt, *J* = 12.7, 6.5 Hz, 6H), 3.99 (dt, *J* = 19.3, 6.6 Hz, 6H), 3.06 (dd, *J* = 15.5, 7.6 Hz, 1H), 2.92 (dd, *J* = 15.5, 5.4 Hz, 1H), 1.89 – 1.72 (m, 12H), 1.60 – 1.54 (m, 8H), 1.53 – 1.43 (m, 12H), 1.37 – 1.22 (m, 90H), 0.97 – 0.84 (m, 18H).**^{13}C NMR** (151 MHz, $CDCl_3$) δ 192.02 (s), 168.74 (s), 164.75 (s), 161.20 (s), 153.33 (s), 153.03 (s), 152.19 (s), 149.83 (s), 149.64 (s), 143.85 (s), 143.31 (s), 141.23 (s), 139.47 (s), 137.97 (s), 135.86 (s), 135.69 (s), 135.32 (s), 134.66 (s), 134.29 (s), 132.73 (s), 132.33 (s), 128.10 (s), 128.02 (s), 124.24 (s), 123.24 (s), 122.00 (s), 121.61 (s), 121.44 (s), 120.98 (s), 118.64 (s), 108.62 (s), 105.97 (s), 73.64 (s), 73.56 (s), 69.31 (s), 69.25 (s), 68.45 (s), 41.11 (s), 31.96 (s), 31.94 (s), 31.45 (s), 30.37 (s), 29.77 (s), 29.76 (s), 29.72 (s), 29.67 (s), 29.66 (s), 29.59 (s), 29.59 (s), 29.45 (s), 29.41 (s), 29.38 (s), 29.30 (s), 26.15 (s), 26.13 (s), 26.10 (s), 26.07 (s), 22.71 (s), 20.15 (s), 14.13 (s). **ESI-HR-MS**: *m/z* for $C_{113}H_{172}O_{13}SNa^+$, ([M]+Na$^+$) calc.: 1792.24109, found: 1792.23502. **Elemental analysis**: for $C_{113}H_{172}O_{13}S$, calc.: C 76.65%, H 9.79%, S 1.81%. found: C 76.48%, H 9. 47%, S 1.60%.

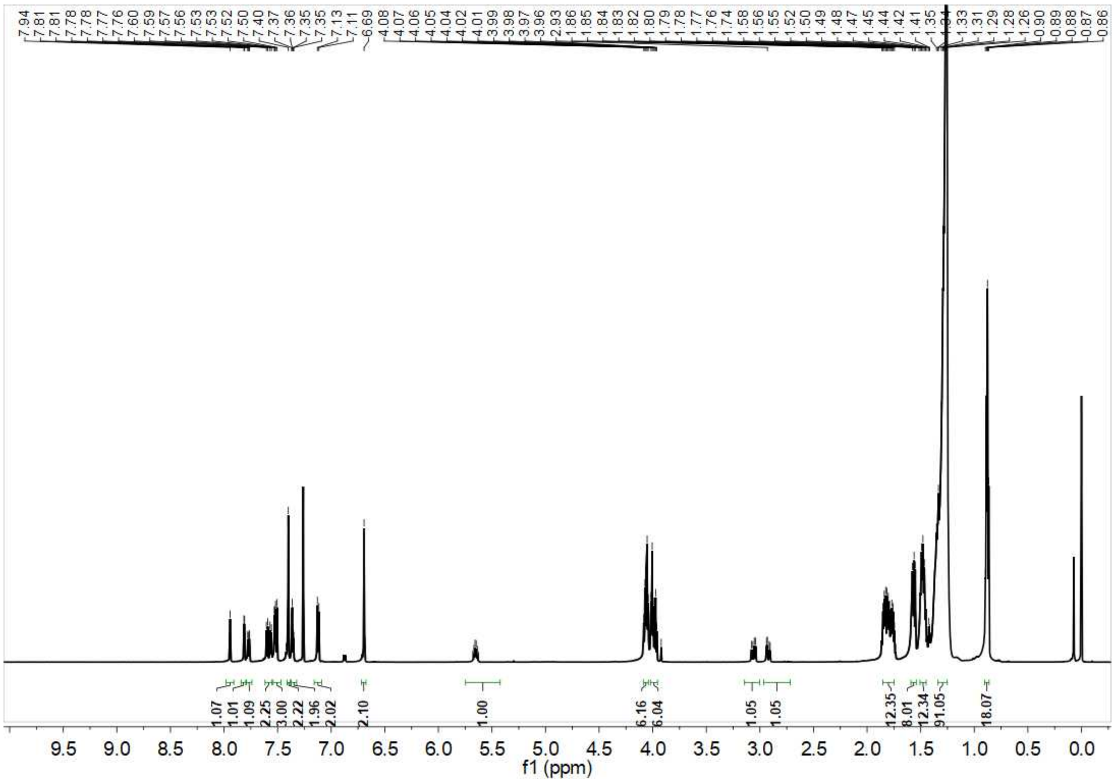

Figure S29. ^{1}H NMR spectrum of ***R*-C12** (600 MHz, $CDCl_3$, 298 K)

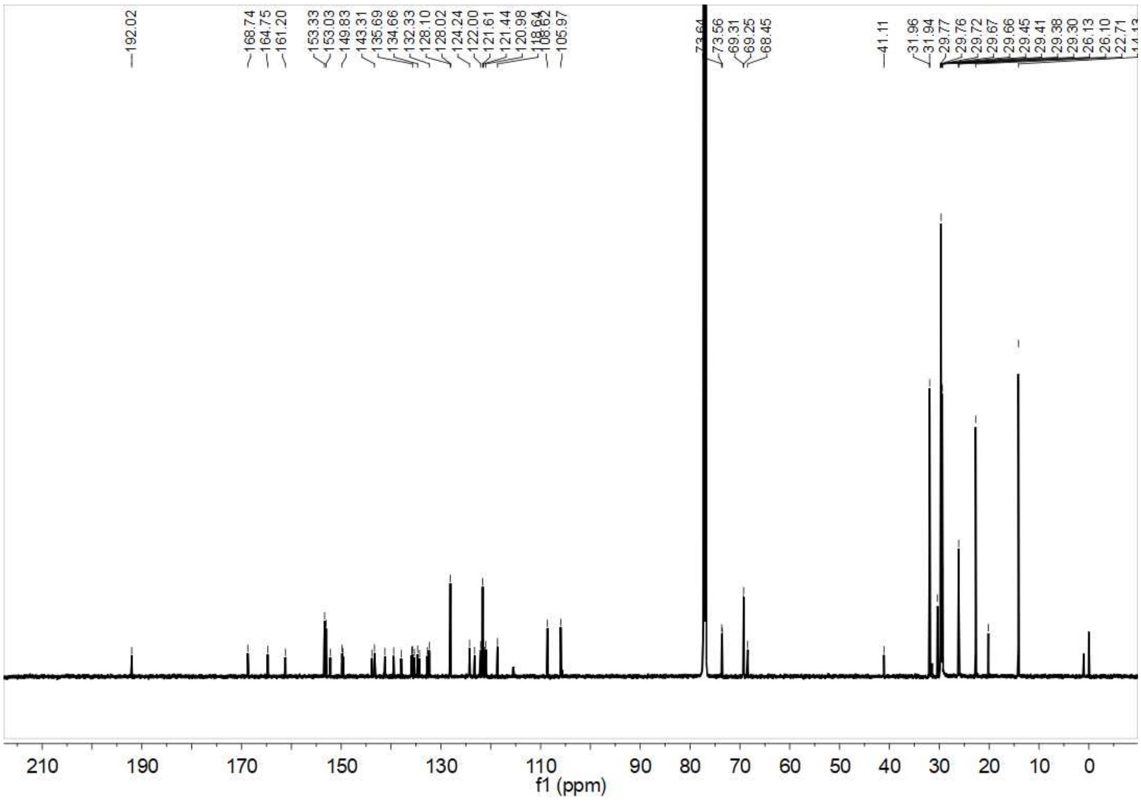

Figure S30. ^{13}C NMR spectrum of ***R*-C12** (150 MHz, $CDCl_3$, 298 K)

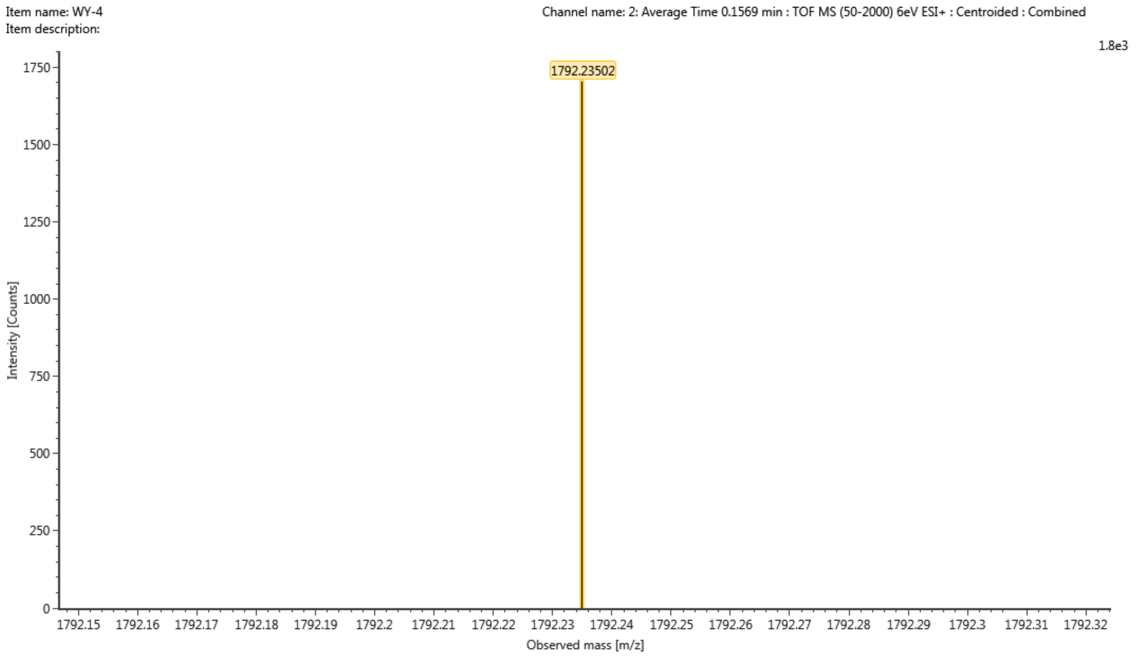

Figure S31. ESI-HR-MS spectrum of ***R*-C12**

7.19 Synthesis of compound N-C10

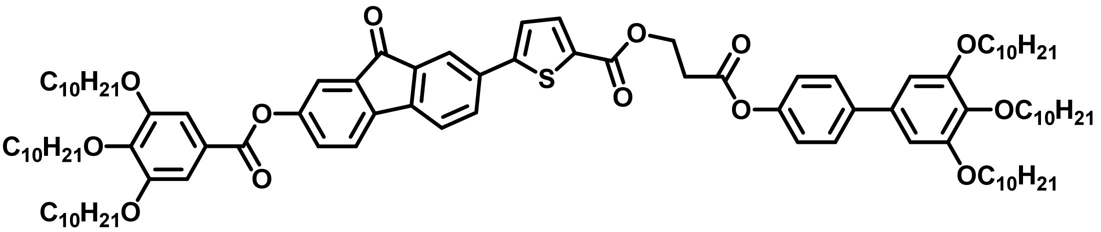

The acid **G-C10** (895mg, 1 mmol) and $SOCl_2$ (5 ml) was refluxing for 30 min. The excess of $SOCl_2$ was removed under vacuum and mixture of dry pyridine (0.5 ml), DCM (5 ml) and 3',4',5'-tris(decyloxy)-[1,1'-biphenyl]-4-yl 3-hydroxypropanoate (710.55 mg, 1 mmol) were added. The resulting mixture was stirred at r.t. overnight. An extra DCM (50 ml) was added and the organic phase was washed with saturated aq NaCl (2 × 20 ml), dried over anhydrous $MgSO_4$, filtered and concentrated. The crude product was purified by SGCC (DCM/PE = 10:1) to obtain the compound **N-C10** as a yellow solid (1 g, 64%). **^{1}H NMR** (600 MHz, $CDCl_3$) δ 7.94 (s, 1H), 7.82 (d, *J* = 3.9 Hz, 1H), 7.77 (d, *J* = 7.8 Hz, 1H), 7.62 – 7.51 (m, 5H), 7.40 (s, 2H), 7.38 – 7.32 (m, 2H), 7.20 – 7.14 (m, 2H), 6.72 (s, 2H), 4.73 (t, *J* = 6.3 Hz, 2H), 4.10 – 3.96 (m, 12H), 3.07 (t, *J* = 6.3 Hz, 2H), 1.83 (dt, *J* = 15.6, 7.3 Hz, 8H), 1.78 – 1.69 (m, 4H), 1.57 (d, *J* = 11.6 Hz, 2H), 1.53 – 1.43 (m, 12H), 1.40 – 1.23 (m, 70H), 0.96 – 0.82 (m, 18H).**^{13}C NMR** (151 MHz, $CDCl_3$) δ 191.97 (s), 169.14 (s), 164.71 (s), 161.65 (s), 153.32 (s), 153.00 (s), 152.17 (s), 150.01 (s), 149.62 (s), 143.87 (s), 143.28 (s), 141.18 (s), 139.50 (s), 137.97 (s), 135.83 (s), 135.62 (s), 135.29 (s), 134.81 (s), 134.18 (s), 132.32 (s), 132.19 (s), 128.09 (s), 128.00 (s), 124.26 (s), 123.20 (s), 121.97 (s), 121.58 (s), 121.42 (s), 120.96 (s), 118.61 (s), 108.58 (s), 105.95 (s), 73.60 (s), 73.53 (s), 69.27 (s), 69.23 (s), 60.47 (s), 34.24 (s), 31.93 (s), 31.89 (s), 30.34 (s), 30.33 (s), 29.74 (s), 29.72 (s), 29.66 (s), 29.63 (s), 29.61 (s), 29.57 (s), 29.55 (s), 29.42 (s), 29.40 (s), 29.38 (s), 29.33 (s), 29.27 (s), 26.12 (s), 26.10 (s), 26.06 (s), 26.03 (s), 22.69 (s), 22.67 (s), 14.10 (s). **ESI-HR-MS**: *m/z* for $C_{100}H_{146}O_{13}SNa^+$, ([M]+$Na^+$), calc.: 1610.03764, found: 1610.03075. **Elemental analysis:** for $C_{100}H_{146}O_{13}S$, calc.: C 75.62%, H 9.27%, S 2.02%, found: C 75.41%, H 9.21%, S 2.29 %.

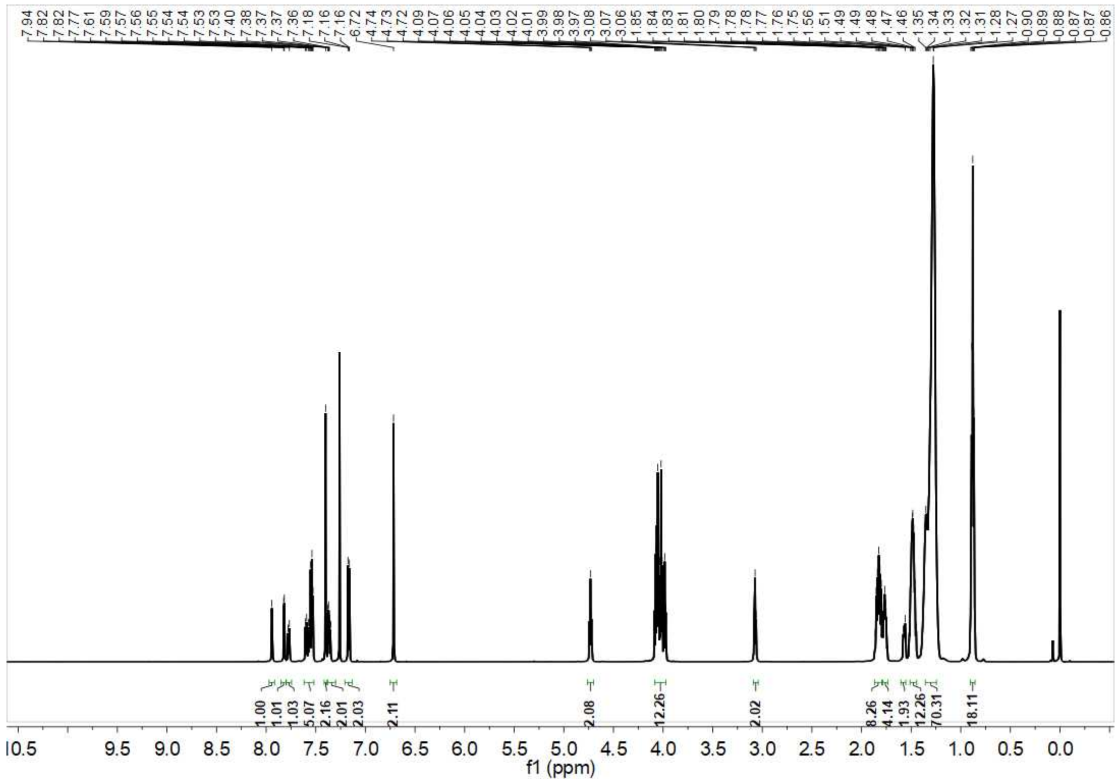

Figure S32. ^{1}H NMR spectrum of **N-C10**(600 MHz, $CDCl_3$, 298 K)

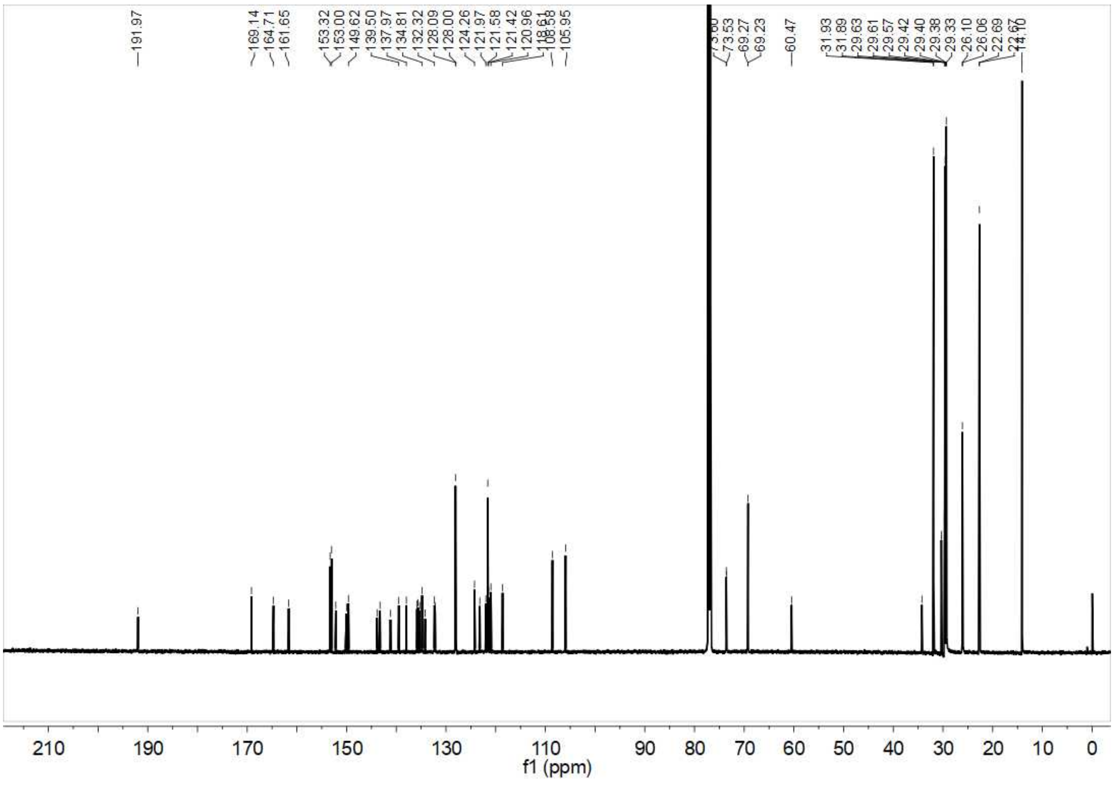

Figure S33. ^{13}C NMR spectrum of **N-C10** (150 MHz, $CDCl_3$, 298 K)

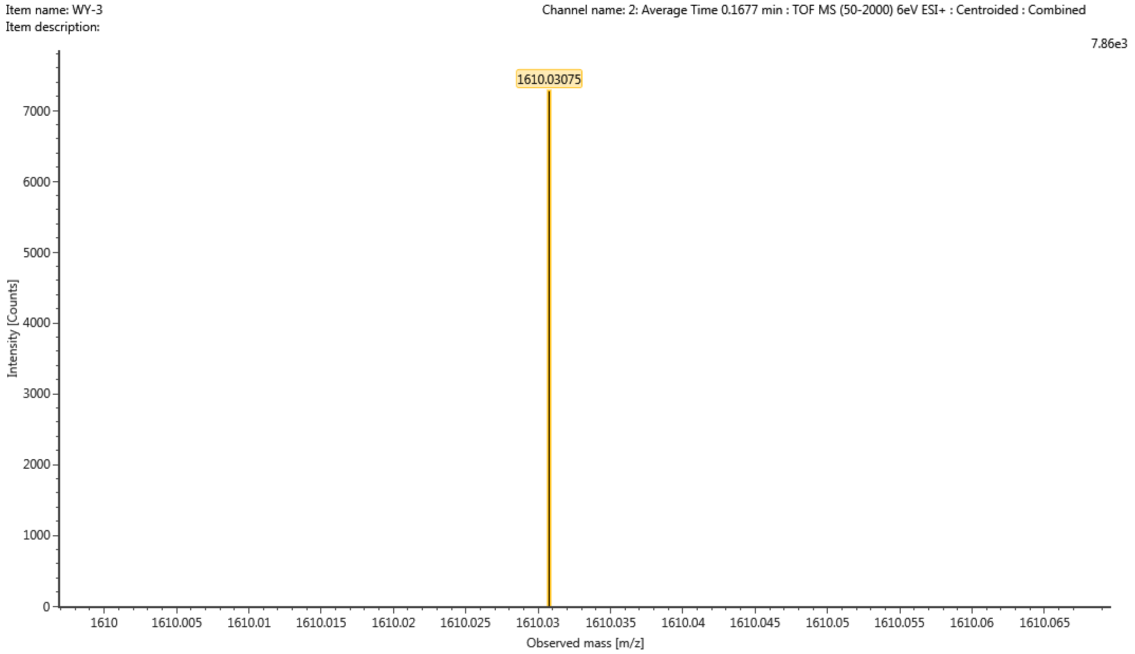

Figure S34. ESI-HR-MS spectrum of **N-C10**

8. References

S1 Kuroda, R.; Berova, N.; Nakanishi, K.; Woody, R. W. "Solid-state CD: application to inorganic and organic chemistry," in Circular dichroism: principles and applications. 2nd ed.; Wiley-VCH, New York, 2000, p. 912.

S2 Arteaga, O.; Freudenthal, J.; Wang, B. L.; Kahr, B. Mueller matrix polarimetry with four photoelastic modulators: theory and calibration. *Appl. Opt.* **2012**, 51, 6805–6817.

S3 Zeng, X.B.; Cseh, L.; Mehl, G.H.; Ungar, G. *J. Mater. Chem.* **2008**, *18*, 2953-2961.

S4 Alam, A.; Tsuboi, S. *Tetrahedron*. **2007**, *63*, 10454-10465.
